# An Inverse QSAR Method Based on a Two-Layered Model and Integer Programming

**DOI:** 10.3390/ijms22062847

**Published:** 2021-03-11

**Authors:** Yu Shi, Jianshen Zhu, Naveed Ahmed Azam, Kazuya Haraguchi, Liang Zhao, Hiroshi Nagamochi, Tatsuya Akutsu

**Affiliations:** 1 Department of Applied Mathematics and Physics, Kyoto University, Kyoto 606-8501, Japan; shi@amp.i.kyoto-u.ac.jp (Y.S.); zhujs@amp.i.kyoto-u.ac.jp (J.Z.); azam@amp.i.kyoto-u.ac.jp (N.A.A.); haraguchi@amp.i.kyoto-u.ac.jp (K.H.); 2 Graduate School of Advanced Integrated Studies in Human Survivability (Shishu-Kan), Kyoto University, Kyoto 606-8306, Japan; liang@gsais.kyoto-u.ac.jp; 3 Bioinformatics Center, Institute for Chemical Research, Kyoto University, Uji 611-0011, Japan; takutsu@kuicr.kyoto-u.ac.jp

**Keywords:** QSAR, molecular design, artificial neural network, mixed integer linear programming, enumeration of graphs, cheminformatics, materials informatics

## Abstract

A novel framework for inverse quantitative structure–activity relationships (inverse QSAR) has recently been proposed and developed using both artificial neural networks and mixed integer linear programming. However, classes of chemical graphs treated by the framework are limited. In order to deal with an arbitrary graph in the framework, we introduce a new model, called a two-layered model, and develop a corresponding method. In this model, each chemical graph is regarded as two parts: the exterior and the interior. The exterior consists of maximal acyclic induced subgraphs with bounded height, the interior is the connected subgraph obtained by ignoring the exterior, and the feature vector consists of the frequency of adjacent atom pairs in the interior and the frequency of chemical acyclic graphs in the exterior. Our method is more flexible than the existing method in the sense that any type of graphs can be inferred. We compared the proposed method with an existing method using several data sets obtained from PubChem database. The new method could infer more general chemical graphs with up to 50 non-hydrogen atoms. The proposed inverse QSAR method can be applied to the inference of more general chemical graphs than before.

## 1. Introduction

Computer-aided design of chemical structures is one of the key topics in chemoinformatics. In particular, extensive studies have been done on inverse quantitative structure–activity relationships (inverse QSAR), which seek chemical structures having desired chemical activities under some constraints. In this framework, chemical compounds are usually represented as vectors of real or integer numbers, which are often called *descriptors* in chemoinformatics and correspond to *feature vectors* in machine learning. Using these chemical descriptors, various heuristic and statistical methods have been developed for inverse QSAR [1,2,3]. In many of such methods, inference or enumeration of graph structures from a given set of descriptors is a crucial subtask. Although various methods have been developed for that purpose [4,5,6,7], enumeration still remains a challenging task because the number of possible chemical graphs is huge, for example, chemical graphs with up to 30 atoms (vertices) C, N, O, and S, may exceed $10^{60}$ [8]. Furthermore, even inference is a challenging task because it is NP-hard (computationally difficult) except for some simple cases [9]. Due to this inherent difficulty, most existing methods for inverse QSAR do not guarantee optimal or exact solutions.

On the other hand, the design of novel graph structures has recently become a hot topic in artificial neural network (ANN) studies, and thus extensive studies have been done for inverse QSAR using ANNs, especially with graph convolutional networks [10]. For example, variational autoencoders [11], recurrent neural networks [12,13], grammar variational autoencoders [14], generative adversarial networks [15], and invertible flow models [16,17] have been applied. Note that QSAR using three-dimensional structures of chemical compounds (3D-QSAR) has also been studied [18]. Particularly, comparative molecular field analysis (CoMFA) has been extensively studied and applied to various molecular design problems [19,20]. In CoMFA, electrostatic potential interaction energies across superimposed molecular structures are used as descriptors and then regression is performed by using the partial least squares (PLS) fitting. Recently, deep neural networks have been applied to 3D-QSAR by combining potential interaction energies with convolutional neural networks [21]. However, in order to apply 3D-QSAR, we need to calculate accurate three-dimensional structures of chemical compounds, which is not a straightforward task.

**Figure 1:**
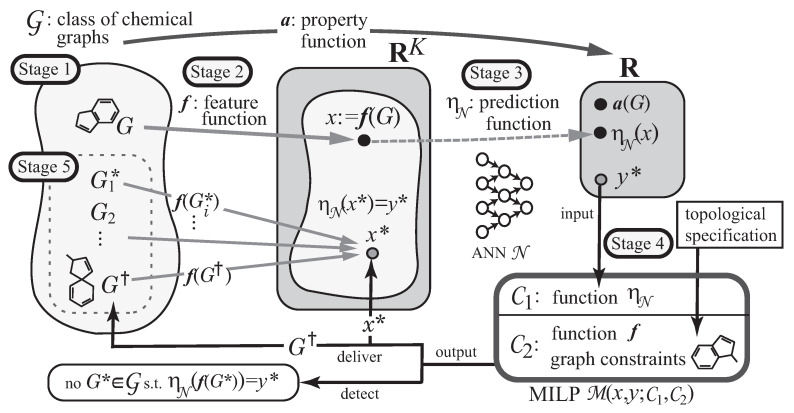
An illustration of a framework for inferring a set of chemical graphs $G^{*}$.

A novel framework for inferring chemical graphs has recently been developed [22,23] based on ANNs and mixed integer linear programming (MILP), as illustrated in Figure 1. It constructs a prediction function in the first phase and infers a chemical graph in the second phase. The first phase of the framework consists of three stages. In Stage 1, we choose a chemical property π and a class G of graphs, where a property function *a* is defined so that a(G) is the value of π in G∈G, and collect a data set $D_{\pi }$ of chemical graphs in G such that a(G) is available. In Stage 2, we introduce a feature function $f:G\to R^{K}$ for a positive integer *K*. In Stage 3, we construct a prediction function $\eta_{N}$ with an ANN N that, given a vector $x\in R^{K}$, returns a value $y=\eta_{N}\left(x\right)\in R$ so that $\eta_{N}\left(f\left(G\right)\right)$ serves as a predicted value to a(G) for each $G\in D_{\pi }$. Given a target chemical value $y^{*}$, the second phase infers chemical graphs $G^{*}$ with $\eta_{N}\left(f\left(G^{*}\right)\right)=y^{*}$ in the next two stages. In Stage 4, we formulate an MILP that simulates the construction of f(G) from *G* and the computation process in the ANN so that given a target value, $y^{*}$, and solve the MILP to infer a chemical graph $G^{†}$ and a feature vector $x^{*}$ such that $f\left(G^{†}\right)=x^{*}$ and $\eta_{N}\left(x^{*}\right)=y^{*}$. In Stage 5, we generate other chemical graphs $G^{*}$ such that $\eta_{N}\left(f\left(G^{*}\right)\right)=y^{*}$ based on the output chemical graph $G^{†}$.

MILP formulations required in Stage 4 have been designed for chemical compounds with cycle index 0 (i.e., acyclic) [23,24], cycle index 1 [25], and cycle index 2 [26]. In particular, Azam et al. [24] introduced a restricted class of acyclic graphs that is characterize by an integer ρ, called a “branch-parameter” such that the restricted class still covers most of the acyclic chemical compounds in the database.

Recently, Akutsu and Nagamochi [27] extended the idea to define a restricted class of cyclic graphs, called “ρ-lean cyclic graphs”, that covers most of the cyclic chemical compounds in the database. Based on this, they also defined a set of rules for specifying several topological substructures of a target chemical graph in a flexible way in Stage 4 before we solve an MILP. The method has been implemented by Zhu et al. [28], and computational results showed that chemical graphs with around up to 50 non-hydrogen atoms can be inferred. Although the method can infer the class of ρ-lean cyclic graphs and specify topological structures of the cyclic part, we still need to introduce a new model to deal with an arbitrary graph and to include a prescribed structure in the acyclic part of a target chemical graph.

**Figure 2:**
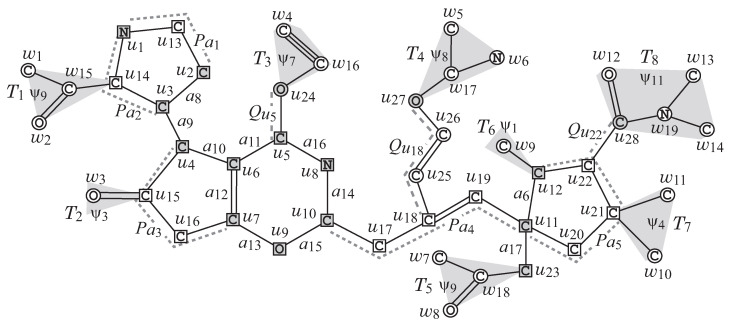
An illustration of a chemical graph *G*, where for ρ=2, the exterior-vertices are $w_{1},w_{2},\ldots ,w_{19}$ and the interior-vertices are $u_{1},u_{2},\ldots ,u_{28}$.

In this paper, we introduce a new model, called a *two-layered model*, for representing the feature of a chemical graph in order to deal with an arbitrary graph in the framework. In the two-layered model, a chemical graph *G* with a parameter ρ≥1 is regarded as two parts: the exterior and the interior. The exterior consists of maximal acyclic induced subgraphs with height at most ρ and the interior is the connected subgraph obtained by ignoring the exterior. We define a feature vector f(G) of a chemical graph *G* to be the frequency of adjacent atom pairs in the interior and the frequency of chemical acyclic graphs in the exterior. Figure 2 illustrates an example of a chemical graph *G*. For a branch-parameter ρ=2, the interior of the chemical graph *G* in Figure 2 is obtained by removing the set of vertices with degree 1 ρ=2 times, i.e., first remove the set $V_{1}=\left\{w_{1},w_{2},\ldots ,w_{14}\right\}$ of vertices of degree 1 in *G*, and then remove the set $V_{2}=\left\{w_{15},w_{16},\ldots ,w_{19}\right\}$ of vertices of degree 1 in $G-V_{1}$, where the removed vertices become the exterior-vertices of *G* and there are eight rooted trees $T_{1},T_{2},\ldots ,T_{8}$ in the exterior of *G*.

**Figure 3:**
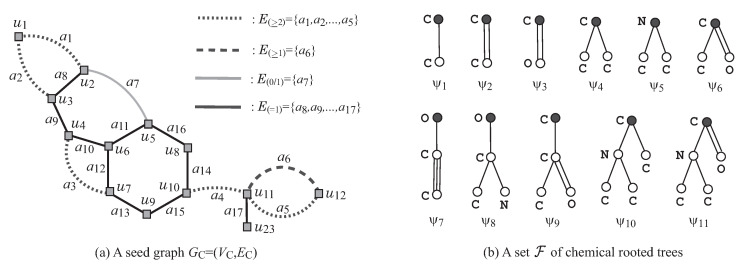
(**a**) An illustration of a seed graph $G_{C}$ where the vertices in $V_{C}$ are depicted with gray squares, the edges in $E_{\left(\geq 2\right)}$ are depicted with dotted lines, the edges in $E_{\left(\geq 1\right)}$ are depicted with dashed lines, the edges in $E_{\left(0/1\right)}$ are depicted with gray bold lines, and the edges in $E_{\left(=1\right)}$ are depicted with black solid lines. (**b**) A set $F=\left\{\psi_{1},\psi_{2},\ldots ,\psi_{11}\right\}\subseteq F\left(D_{\pi }\right)$ of 11 chemical rooted trees $\psi_{i},i\in \left[1,11\right]$, where the root of each tree is depicted with a black circle.

We also introduce a new set of rules for specifying topological substructures of a target chemical graph *G* to be inferred so that a prescribed structure can be included in both of the acyclic and cyclic parts of *G*. The set of rules contains (i) a *seed graph* $G_{C}$ as an abstract form of a target chemical graph *G*; (ii) a set F of chemical rooted trees as candidates for trees in the exterior of *G*; and (iii) lower and upper bounds on the number of components in a target chemical graph such as chemical elements, double/triple bounds and the interior-vertices in *G*. Figure 3a,b illustrates examples of a seed graph $G_{C}$ and a set F of chemical rooted trees, respectively. Given a seed graph $G_{C}$, the interior of a target chemical graph *G* is constructed from $G_{C}$ by replacing some edges a=uv with paths $P_{a}$ between the end-vertices *u* and *v*, and by attaching new paths $Q_{v}$ to some vertices *v*. For example, the chemical graph *G* in Figure 2 is constructed from the seed graph $G_{C}$ in Figure 3a as follows. First replace five edges $a_{1}=u_{1}u_{2},a_{2}=u_{1}u_{3},a_{3}=u_{4}u_{7},a_{4}=u_{10}u_{11}$ and $a_{5}=u_{11}u_{12}$ in $G_{C}$ with new paths $P_{a_{1}}=\left(u_{1},u_{13},u_{2}\right)$, $P_{a_{2}}=\left(u_{1},u_{14},u_{3}\right)$, $P_{a_{3}}=\left(u_{4},u_{15},u_{16},u_{7}\right)$, $P_{a_{4}}=\left(u_{10},u_{17},u_{18},u_{19},u_{11}\right)$ and $P_{a_{5}}=\left(u_{11},u_{20},u_{21},u_{22},u_{12}\right)$, respectively, to obtain the subgraph $G_{1}$ of *G* that consists of vertices depicted with squares. Next, attach to this graph $G_{1}$ three new paths, $Q_{u_{5}}=\left(u_{5},u_{24}\right)$, $Q_{u_{18}}=\left(u_{18},u_{25},u_{26},u_{27}\right)$, and $Q_{u_{22}}=\left(u_{22},u_{28}\right)$, to obtain the interior of *G* in Figure 2. Finally, the chemical graph *G* in Figure 2 is obtained by attaching eight trees $T_{1},T_{2},\ldots ,T_{8}$ selected from the set F and assigning chemical elements and bond-multiplicities in the interior. The frequency of chemical elements and the graph size are controlled with lower and upper bounds on the components in a target chemical graph *G*. See Section 2.2 for more details on the specification.

We implemented the two-layered model and the results of computational experiments suggest that the proposed method can infer chemical graphs with around up to 50 non-hydrogen atoms.

The paper is organized as follows. Section 2.1 introduces some notions on graphs, a modeling of chemical compounds, and a choice of descriptors. Section 2.2 introduces a method of specifying topological substructures of target chemical graphs in Stage 4. Section 3 reports the results on some computational experiments conducted for chemical properties such as octanol/water partition coefficient, boiling point, melting point, flash point, lipophylicity, and solubility. Section 4 makes some concluding remarks. An MILP formulation used in Stage 4 and a review of the dynamic programming algorithm for generating isomers in Stage 5 can be found in Supplementary Materials. The proposed method/system is available at GitHub https://github.com/ku-dml/mol-infer.

## 2. Materials and Methods

This section presents mathematical details of our developed method. Readers not interested in mathematical details can skip this section.

### 2.1. Preliminary

This section introduces some notions and terminology on graphs, a modeling of chemical compounds, and our choice of descriptors.

Let R, Z and $Z_{+}$ denote the sets of reals, integers and non-negative integers, respectively. For two integers *a* and *b*, let [a,b] denote the set of integers *i* with a≤i≤b.

**Graphs.** Given a graph *G*, let V(G) and E(G) denote the sets of vertices and edges, respectively. For a subset $V^{\prime }\subseteq V\left(G\right)$ (resp., $E^{\prime }\subseteq E\left(G\right))$ of a graph *G*, let $G-V^{\prime }$ (resp., $G-E^{\prime }$) denote the graph obtained from *G* by removing the vertices in $V^{\prime }$ (resp., the edges in $E^{\prime }$), where we remove all edges incident to a vertex in $V^{\prime }$ in $G-V^{\prime }$. The *rank* r(G) of a graph *G* is defined to be the minimum |F| of an edge subset F⊆E(G) such that G−F contains no cycle. A path with two end-vertices *u* and *v* is called a *u,v-path*. An edge $e=u_{1}u_{2}$ in a connected graph *G* is called a *bridge* if the graph G−e obtained from *G* by removing edge *e* is not connected, i.e., G−e consists of two connected graphs $G_{i}$ containing vertex $u_{i}$, i=1,2. For a cyclic graph *G*, an edge *e* is called a *core-edge* if it is in a cycle of *G* or is a bridge $e=u_{1}u_{2}$ such that each of the connected graphs $G_{i}$, i=1,2 of G−e contains a cycle. A vertex incident to a core-edge is called a *core-vertex* of *G*.

A vertex designated in a graph *G* is called a *root*. In this paper, we designated at most two vertices as roots, and denote by Rt(G) the set of roots of *G*. We call a graph *G* *rooted* (resp., *bi-rooted*) if |Rt(G)|=1 (resp., |Rt(G)|=2), where we call *Gunrooted* if Rt(G)=∅.

For a graph *G*, possibly with roots, a *leaf-vertex* is defined to be a non-root vertex v∈V(G)\Rt(G) with degree 1, call the edge uv incident to a leaf vertex *v* a *leaf-edge*, and denote $V_{\mathrm{leaf}}\left(G\right)$ and $E_{\mathrm{leaf}}\left(G\right)$ the sets of leaf-vertices and leaf-edges in *G*, respectively. For a graph or a rooted graph *G*, we define graphs $G_{i},i\in Z_{+}$ obtained from *G* by removing the set of leaf-vertices *i* times so that

$$G_{0}:=G;\;\;G_{i+1}:=G_{i}-V_{\mathrm{leaf}}\left(G_{i}\right),$$

where we call a vertex $v\in V_{\mathrm{leaf}}\left(G_{k}\right)$ a *leaf k-branch* and we say that a vertex $v\in V_{\mathrm{leaf}}\left(G_{k}\right)$ has height *height* ht(v)=k in *G*. The *height* ht(T) of a rooted tree *T* is defined to be the maximum of ht(v) of a vertex v∈V(T). For an integer k≥0, we call a rooted tree *T* *k-lean* if *T* has at most one leaf *k*-branch. For an unrooted cyclic graph *G*, we regard the set of non-core-edges in *G* induces a collection T of trees each of which is rooted at a core-vertex, where we call *G k-lean* if each of the rooted trees in T is *k*-lean. Nearly 97% of cyclic chemical compounds with up to 100 non-hydrogen atoms in PubChem are 2-lean [24]. 

**Two-layered Model.** Let *G* be an unrooted graph. For an integer ρ≥0, which we call a *branch-parameter*, a *two-layered model* of *G* is a partition of *G* into an “interior” and an “exterior” in the following way. We call a vertex v∈V(G) (resp., an edge e∈E(G)) of *G* an *exterior-vertex* (resp., *exterior-edge*) if ht(v)<ρ (resp., *e* is incident to an exterior-vertex) and denote the sets of exterior-vertices and exterior-edges by $V^{\mathrm{ex}}\left(G\right)$ and $E^{\mathrm{ex}}\left(G\right)$, respectively and denote $V^{\mathrm{int}}\left(G\right)=V\left(G\right)\backslash V^{\mathrm{ex}}\left(G\right)$ and $E^{\mathrm{int}}\left(G\right)=E\left(G\right)\backslash E^{\mathrm{ex}}\left(G\right)$, respectively. We call a vertex in $V^{\mathrm{int}}\left(G\right)$ (resp., an edge in $E^{\mathrm{int}}\left(G\right)$) an *interior-vertex* (resp., *interior-edge*). The set $E^{\mathrm{ex}}\left(G\right)$ of exterior-edges forms a collection of connected graphs each of which is regarded as a rooted tree *T* rooted at the vertex v∈V(T) with the maximum ht(v), where we call *T* a *ρ-fringe-tree* (or a fringe-tree). Let $T^{\mathrm{ex}}\left(G\right)$ denote the set of fringe-trees in *G*. The *interior* of *G* is defined to be the subgraph $\left(V^{\mathrm{int}}\left(G\right),E^{\mathrm{int}}\left(G\right)\right)$ of *G*. Note that every core-vertex (resp., core-edge) in *G* is an interior-vertex (resp., interior-edge) of *G*. Figure 2 illustrates an example of a graph *G*, such that $V^{\mathrm{int}}=\left\{u_{1},u_{2},\ldots ,u_{28}\right\}$, $V^{\mathrm{ex}}=\left\{w_{1},w_{2},\ldots ,w_{19}\right\}$ and $T^{\mathrm{ex}}\left(G\right)=\left\{T_{1},T_{2},\ldots ,T_{8}\right\}$ for a branch-parameter ρ=2.

#### 2.1.1. Modeling of Chemical Compounds

To represent a chemical compound, we assume that each chemical element a has a unique valence val(a)∈[1,4] and we use a hydrogen-suppressed model, because hydrogen atoms can be added at the final stage under the assumption. In the hydrogen-suppressed model, a chemical compound *C* is represented by a tuple G=(H,α,β) of a simple, connected undirected graph *H* and functions α:V(H)→Λ and β:E(H)→[1,3], where Λ is a set of non-hydrogen chemical elements such as C (carbon), O (oxygen), N (nitrogen), and so on. The set of atoms and the set of bonds in the compound *C* are represented by the vertex set V(H) and the edge set E(H), respectively. The chemical element assigned to a vertex v∈V(H) is represented by α(v) and the bond-multiplicity between two adjacent vertices u,v∈V(H) is represented by β(e) of the edge e=uv∈E(H). We say that two tuples $(H_{i},\alpha_{i},\beta_{i}),i=1,2$ are *isomorphic* if they admit an isomorphism ϕ, i.e., a bijection $\phi :V\left(H_{1}\right)\to V\left(H_{2}\right)$ such that $uv\in E\left(H_{1}\right),\alpha_{1}\left(u\right)=a,\alpha_{1}\left(v\right)=b,\beta_{1}\left(uv\right)=m$ ↔ $\phi \left(u\right)\phi \left(v\right)\in E\left(H_{2}\right),\alpha_{2}\left(\phi \left(u\right)\right)=a,\alpha_{2}\left(\phi \left(v\right)\right)=b,\beta_{2}\left(\phi \left(u\right)\phi \left(v\right)\right)=m$. When $H_{i}$ is rooted at a vertex $r_{i},i=1,2$, $(H_{i},\alpha_{i},\beta_{i}),i=1,2$ are *rooted-isomorphic* (r-isomorphic) if they admit an isomorphism ϕ such that $\phi \left(r_{1}\right)=r_{2}$. Chemical rooted trees $T_{1}$ and $T_{5}$ in Figure 2 are r-isomorphic.

Associated with the two functions α and β in a tuple G=(H,α,β), we introduce the following functions: $\beta_{G}:V\left(H\right)\to \left[0,12\right]$, ac:V(E)→Λ×Λ×[1,3], cs:V(E)→Λ×[1,4], and ec:V(E)→(Λ×[1,4])×(Λ×[1,4])×[1,3].

For a notational convenience, we use a function $\beta_{G}:V\left(H\right)\to \left[0,4\right]$ such that $\beta_{G}\left(u\right)$ means the sum of bond-multiplicities of edges incident to a vertex *u*, i.e.,

$$\beta_{G}\left(u\right)≜\sum_{uv\in E(H)}\beta \left(uv\right)\;\mathrm{for}\;\mathrm{each}\;\mathrm{vertex}\;u\in V(H).$$

A *chemical graph* *G* is defined to be a tuple (H,α,β) such that the valence condition at each vertex v∈V(H) is satisfied, i.e.,

$$\beta_{G}\left(v\right)\leq \mathrm{val}\left(\alpha \left(v\right)\right),$$

where we define the *hydro-degree* $\deg_{\mathrm{hyd}}\left(v\right)$ of a vertex *v* to be $\mathrm{val}\left(\alpha \left(v\right)\right)-\beta_{G}\left(v\right)$.

Figure 2 illustrates an example of a chemical graph G=(H,α,β).

To represent a feature of an edge e=uv∈E(H) such that α(u)=a, α(v)=b and β(e)=m in a chemical graph G=(H,α,β), we use a tuple (a,b,m)∈Λ×Λ×[1,3], which we call the *adjacency-configuration* ac(e) of the edge *e*. We introduce a total order < over the elements in Λ to distinguish with (a,b,m) and (b,a,m) (a≠b) notationally. For a tuple ν=(a,b,m), let $\bar{\nu }$ denote the tuple (b,a,m).

To represent a feature of a vertex v∈V(H) with α(v)=a that has *d* atoms in its neighbor in a chemical graph G=(H,α,β), we use a pair (a,d)∈Λ×[1,4], which we call the *chemical symbol* cs(v) of the vertex *v*. We treat (a,d) as a single symbol ad, and define $\Lambda_{\mathrm{dg}}$ to be the set of all chemical symbols μ=ad∈Λ×[1,4].

To represent a feature of an edge e=uv∈E(H) such that cs(u)=μ, cs(v)=ξ and β(e)=m in a chemical graph G=(H,α,β), we use a tuple $\left(\mu ,\xi ,m\right)\in \Lambda_{\mathrm{dg}}\times \Lambda_{\mathrm{dg}}\times \left[1,3\right]$, which we call the *edge-configuration* ec(e) of the edge *e*. We introduce a total order < over the elements in $\Lambda_{\mathrm{dg}}$ to distinguish with (μ,ξ,m) and (ξ,μ,m) (μ≠ξ) notationally. For a tuple γ=(μ,ξ,m), let $\bar{\gamma }$ denote the tuple (ξ,μ,m).

To represent a feature of the exterior of a chemical graph G=(H,α,β), a ρ-fringe-tree in $T^{\mathrm{ex}}\left(G\right)$ is called a *fringe-configuration* in the exterior.

#### 2.1.2. Introducing Descriptors of Feature Vectors

This section introduces descriptors to define our feature vectors. Let π be a chemical property for which we will construct a prediction function $\eta_{N}$ from a feature vector f(G) of a chemical graph to a predicted value y∈R for the chemical property of *G*.

We first choose a set Λ of non-hydrogen chemical elements and then collect a data set $D_{\pi }$ of chemical compounds *C* whose chemical elements belong to Λ∪{H}, where we regard $D_{\pi }$ as a set of chemical graphs that represent the chemical compounds *C* in $D_{\pi }$. To define the interior/exterior of chemical graphs $G\in D_{\pi }$, we next choose a branch-parameter ρ, where we recommend ρ=2.

Let $\Lambda^{\mathrm{int}}\left(D_{\pi }\right)$ (resp., $\Lambda^{\mathrm{ex}}\left(D_{\pi }\right)$) denote the set of chemical elements used in the set of interior-vertices (resp., exterior-vertices) over all chemical graphs $G\in D_{\pi }$, and $\Gamma^{\mathrm{int}}\left(D_{\pi }\right)$ denote the set of edge-configurations used in the set of interior-edges over all chemical graphs $G\in D_{\pi }$. Let $F(D_{\pi })$ denote the set of chemical rooted trees ψ r-isomorphic to a ρ-fringe-tree $T\in T^{\mathrm{ex}}\left(G\right)$ over all chemical graphs $G\in D_{\pi }$.

We define an integer encoding of a finite set *A* of elements to be a bijection σ:A→[1,|A|], where we denote by [A] the set [1,|A|] of integers. Introduce an integer coding of each of the sets $\Lambda^{\mathrm{int}}\left(D_{\pi }\right)$, $\Lambda^{\mathrm{ex}}\left(D_{\pi }\right)$, $\Gamma^{\mathrm{int}}\left(D_{\pi }\right)$ and $F(D_{\pi })$. Let ${\left[a\right]}^{\mathrm{int}}$ (resp., ${\left[a\right]}^{\mathrm{ex}}$) denote the coded integer of an element $a\in \Lambda^{\mathrm{int}}\left(D_{\pi }\right)$ (resp., $a\in \Lambda^{\mathrm{ex}}\left(D_{\pi }\right)$), [γ] denote the coded integer of an element γ in $\Gamma^{\mathrm{int}}\left(D_{\pi }\right)$ and [ψ] denote an element ψ in $F(D_{\pi })$.

For each chemical element a∈Λ, let mass(a) and val(a) denote the mass and valence of a, respectively. In our model, we use integers ${\mathrm{mass}}^{*}\left(a\right)=\left\lfloor 10\cdot \mathrm{mass}\left(a\right)\right\rfloor$, a∈Λ.

We define the *feature vector* f(G) of a chemical graph $G=\left(H,\alpha ,\beta \right)\in D_{\pi }$ to be a vector that consists of the following non-negative integer descriptors ${\mathrm{dcp}}_{i}\left(G\right)$, i∈[1,K], where $K=17+|\Lambda^{\mathrm{int}}\left(D_{\pi }\right)\left|+\right|\Lambda^{\mathrm{ex}}\left(D_{\pi }\right)\left|+\right|\Gamma^{\mathrm{int}}\left(D_{\pi }\right)|+|F\left(D_{\pi }\right)|$.

1. ${\mathrm{dcp}}_{1}\left(G\right)$: the number n(G)=|V(G)| of vertices in *G*.
2. ${\mathrm{dcp}}_{2}\left(G\right)$: the number $|V^{\mathrm{int}}\left(G\right)|$ of interior-vertices in *G*.
3. ${\mathrm{dcp}}_{3}\left(G\right)$: the average $\bar{\mathrm{ms}}\left(G\right)$ of mass${}^{*}$ over all non-hydrogen atoms in *G*, i.e., $\bar{\mathrm{ms}}\left(G\right)≜\sum_{v\in V(G)}{\mathrm{mass}}^{*}\left(\alpha \left(v\right)\right)/n\left(G\right)$.
4. ${\mathrm{dcp}}_{i}\left(G\right)$, i=3+d,d∈[1,4]: the number ${\mathrm{dg}}_{d}\left(G\right)$ of interior-vertices of degree *d* in *G*.
5. ${\mathrm{dcp}}_{i}\left(G\right)$, i=7+d,d∈[1,4]: the number ${\mathrm{dg}}_{d}^{\mathrm{int}}\left(G\right)$ of interior-vertices of interior-degree $\deg_{\left(V^{\mathrm{int}},E^{\mathrm{int}}\right)}\left(v\right)=d$ in the interior $\left(V^{\mathrm{int}},E^{\mathrm{int}}\right)$ of *G*.
6. ${\mathrm{dcp}}_{i}\left(G\right)$, i=11+d,d∈[0,3]: the number ${\mathrm{hydg}}_{d}\left(G\right)$ of vertices in *G* of hydro-degree $\deg_{\mathrm{hyd}}\left(v\right)=d$.
7. ${\mathrm{dcp}}_{i}\left(G\right)$, i=15+m, m∈[2,3]: the number ${\mathrm{bd}}_{m}^{\mathrm{int}}\left(G\right)$ of interior-edges with bond multiplicity *m* in *G*, i.e., ${\mathrm{bd}}_{m}^{\mathrm{int}}\left(G\right)≜\left\{e\in E^{\mathrm{int}}|\beta \left(e\right)=m\right\}$.
8. ${\mathrm{dcp}}_{i}\left(G\right)$, $i=17+{\left[a\right]}^{\mathrm{int}}$, $a\in \Lambda^{\mathrm{int}}\left(D_{\pi }\right)$: the frequency ${\mathrm{na}}_{a}^{\mathrm{int}}\left(G\right)$ of chemical element a in the set of interior-vertices in *G*.
9. ${\mathrm{dcp}}_{i}\left(G\right)$, $i=17+|\Lambda^{\mathrm{int}}\left(D_{\pi }\right)|+{\left[a\right]}^{\mathrm{ex}}$, $a\in \Lambda^{\mathrm{ex}}\left(D_{\pi }\right)$: the frequency ${\mathrm{na}}_{a}^{\mathrm{ex}}\left(G\right)$ of chemical element a in the set of exterior-vertices in *G*.
10. ${\mathrm{dcp}}_{i}\left(G\right)$, $i=17+|\Lambda^{\mathrm{int}}\left(D_{\pi }\right)\left|+\right|\Lambda^{\mathrm{ex}}\left(D_{\pi }\right)|+\left[\gamma \right]$, $\gamma \in \Gamma^{\mathrm{int}}\left(D_{\pi }\right)$: the frequency ${\mathrm{ec}}_{\gamma }\left(G\right)$ of edge-configuration γ in the set of interior-edges $e\in E^{\mathrm{int}}$ in *G*.
11. ${\mathrm{dcp}}_{i}\left(G\right)$, $i=17+|\Lambda^{\mathrm{int}}\left(D_{\pi }\right)\left|+\right|\Lambda^{\mathrm{ex}}\left(D_{\pi }\right)\left|+\right|\Gamma^{\mathrm{int}}\left(D_{\pi }\right)|+\left[\psi \right]$, $\psi \in F(D_{\pi })$: the frequency ${\mathrm{fc}}_{\psi }\left(G\right)$ of fringe-configuration ψ in the set of ρ-fringe-trees in *G*.

### 2.2. Specifying Target Chemical Graphs

Given a prediction function $\eta_{N}$ and a target value $y^{*}\in R$, we call a chemical graph $G^{*}$ such that $\eta_{N}\left(x^{*}\right)=y^{*}$ for the feature vector $x^{*}=f\left(G^{*}\right)$ a *target chemical graph*. This section presents a set of rules for specifying topological substructure of a target chemical graph in a flexible way in Stage 4.

We first describe how to reduce a chemical graph G=(H,α,β) into an abstract form based on which our specification rules will be defined. To illustrate the reduction process, we use the chemical graph G=(H,α,β) in Figure 2.

**Figure 4:**
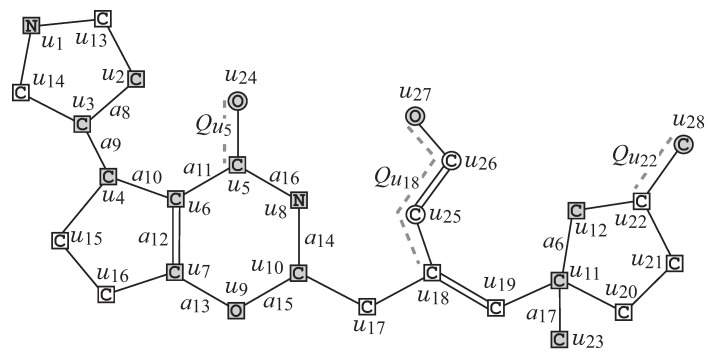
The interior $H^{\mathrm{int}}$ of chemical graph *G* with ρ=2 in Figure 2.

**Figure 5:**
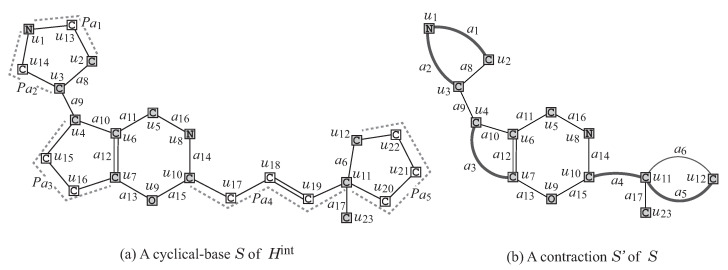
(**a**) A cyclical-base $S=H^{\mathrm{int}}-{⋃}_{u\in \{u_{5},u_{18},u_{22}\}}\left(V\left(Q_{u}\right)\backslash \left\{u\right\}\right)$ of the interior $H^{\mathrm{int}}$ in Figure 4; (**b**) A contraction $S^{\prime }$ of *S* for a pure path set $P=\{P_{a_{1}},P_{a_{2}},\ldots ,P_{a_{5}}\}$ in (**a**), where a new edge obtained by contracting a pure path is depicted with a thick line.

R1 **Removal of all ρ-fringe-trees:** The interior $H^{\mathrm{int}}=\left(V^{\mathrm{int}}\left(H\right),E^{\mathrm{int}}\left(H\right)\right)$ of *G* is obtained by removing the non-root vertices of each ρ-fringe-trees $T\in T^{\mathrm{ex}}\left(G\right)$. Figure 4 illustrates the interior $H^{\mathrm{int}}$ of chemical graph *G* with ρ=2 in Figure 2.
R2 **Removal of some leaf paths:** We call a u,v-path *Q* in $H^{\mathrm{int}}$ a *leaf path* if vertex *v* is a leaf-vertex of $H^{\mathrm{int}}$ and the degree of each internal vertex of *Q* in $H^{\mathrm{int}}$ is 2, where we regard that *Q* is rooted at vertex *u*. A connected subgraph *S* of the interior $H^{\mathrm{int}}$ of *G* is called a *cyclical-base* if *S* is obtained from *H* by removing the vertices in $V\left(Q_{u}\right)\backslash \left\{u\right\},u\in X$ for a subset *X* of interior-vertices and a set $\left\{Q_{u}|u\in X\right\}$ of leaf u,v-paths $Q_{u}$ such that no two paths $Q_{u}$ and $Q_{u^{\prime }}$ share a vertex. Figure 5a illustrates a cyclical-base $S=H^{\mathrm{int}}-{⋃}_{u\in X}\left(V\left(Q_{u}\right)\backslash \left\{u\right\}\right)$ of the interior $H^{\mathrm{int}}$ for a set $\left\{Q_{u_{5}}=\left(u_{5},u_{24}\right),Q_{u_{18}}=\left(u_{18},u_{25},u_{26},u_{27}\right),Q_{u_{22}}=\left(u_{22},u_{28}\right)\right\}$ of leaf paths in Figure 4.
R3 **Contraction of some pure paths:** A path in *S* is called *pure* if each internal vertex of the path is of degree 2. Choose a set P of several pure paths in *S* so that no two paths share vertices except for their end-vertices. A graph $S^{\prime }$ is called a *contraction* of a graph *S* (with respect to P) if $S^{\prime }$ is obtained from *S* by replacing each pure u,v-path with a single edge a=uv, where $S^{\prime }$ may contain multiple edges between the same pair of adjacent vertices. Figure 5b illustrates a contraction $S^{\prime }$ obtained from the chemical graph *S* by contracting each uv-path $P_{a}\in P$ into a new edge a=uv, where $a_{1}=u_{1}u_{2},a_{2}=u_{1}u_{3},a_{3}=u_{4}u_{7},a_{4}=u_{10}u_{11}$, and $a_{5}=u_{11}u_{12}$, and $P=\{P_{a_{1}}=\left(u_{1},u_{13},u_{2}\right),P_{a_{2}}=\left(u_{1},u_{14},u_{3}\right),P_{a_{3}}=\left(u_{4},u_{15},u_{16},u_{7}\right),P_{a_{4}}=\left(u_{10},u_{17},u_{18},u_{19},u_{11}\right),P_{a_{5}}=\left(u_{11},u_{20},u_{21},u_{22},u_{12}\right)\}$ of pure paths in Figure 5a.

We will define a set of rules so that a chemical graph can be obtained from a graph (called a seed graph in the next section) by applying processes R3 to R1 in a reverse way. We specify topological substructures of a target chemical graph with a tuple $\left(G_{C},\sigma_{\mathrm{int}},\sigma_{\mathrm{ce}}\right)$ called a *target specification* defined under the set of the following rules.

Seed Graphs

A *seed graph* $G_{C}=\left(V_{C},E_{C}\right)$ is defined to be a graph (possibly with multiple edges) such that the edge set $E_{C}$ consists of four sets $E_{\left(\geq 2\right)}$, $E_{\left(\geq 1\right)}$, $E_{\left(0/1\right)}$, and $E_{\left(=1\right)}$, where each of them can be empty. A seed graph plays a role of the most abstract form $S^{\prime }$ in R3. Figure 3a illustrates an example of a seed graph, where $V_{C}=\left\{u_{1},u_{2},\ldots ,u_{12}\right\}$, $E_{\left(\geq 2\right)}=\left\{a_{1},a_{2},\ldots ,a_{5}\right\}$, $E_{\left(\geq 1\right)}=\left\{a_{6}\right\}$, $E_{\left(0/1\right)}=\left\{a_{7}\right\}$, and $E_{\left(=1\right)}=\left\{a_{8},a_{9},\ldots ,a_{17}\right\}$.

A *subdivision* *S* of $G_{C}$ is a graph constructed from a seed graph $G_{C}$ according to the following rules:

- Each edge $e=uv\in E_{\left(\geq 2\right)}$ is replaced with a u,v-path $P_{e}$ of length at least 2;
- Each edge $e=uv\in E_{\left(\geq 1\right)}$ is replaced with a u,v-path $P_{e}$ of length at least 1 (equivalently *e* is directly used or replaced with a u,v-path $P_{e}$ of length at least 2);
- Each edge $e\in E_{\left(0/1\right)}$ is either used or discarded; and
- Each edge $e\in E_{\left(=1\right)}$ is always used directly.

We allow a possible elimination of edges in $E_{\left(0/1\right)}$ as an optional rule in constructing a target chemical graph from a seed graph, even though such an operation has not been included in the process R3. A subdivision *S* plays a role of a cyclical-base in R2. A target chemical graph G=(H,α,β) will contain *S* as a subgraph of the interior $H^{\mathrm{int}}$ of *G*.

Interior-Specification

A graph $H^{*}$ that serves as the interior $H^{\mathrm{int}}$ of a target chemical graph *G* will be constructed as follows. First, construct a subdivision *S* of a seed graph $G_{C}$ by replacing each edge edge $e=uu^{\prime }\in E_{\left(\geq 2\right)}\cup E_{\left(\geq 1\right)}$ with a pure $u,u^{\prime }$-path $P_{e}$. Next, construct a supergraph $H^{*}$ of *S* by attaching a leaf path $Q_{v}$ at each vertex $v\in V_{C}$ or at an internal vertex $v\in V\left(P_{e}\right)\backslash \left\{u,u^{\prime }\right\}$ of each pure $u,u^{\prime }$-path $P_{e}$ for some edge $e=uu^{\prime }\in E_{\left(\geq 2\right)}\cup E_{\left(\geq 1\right)}$, where possibly $Q_{v}=v,E\left(Q_{v}\right)=\emptyset$ (i.e., we do not attach any new edges to *v*). We introduce the following rules for specifying the size of $H^{*}$, the length $\left|E(P_{e})\right|$ of a pure path $P_{e}$, the length $\left|E(Q_{v})\right|$ of a leaf path $Q_{v}$, the number of leaf paths $Q_{v}$, and a bond-multiplicity of each interior-edge, where we call the set of prescribed constants an *interior-specification* $\sigma_{\mathrm{int}}$:

- Lower and upper bounds $n_{\mathrm{LB}}^{\mathrm{int}},n_{\mathrm{UB}}^{\mathrm{int}}\in Z_{+}$ on the number of interior-vertices of a target chemical graph *G*.
- For each edge $e=uu^{\prime }\in E_{\left(\geq 2\right)}\cup E_{\left(\geq 1\right)}$,
  a lower bound $\ell_{\mathrm{LB}}\left(e\right)$ and an upper bound $\ell_{\mathrm{UB}}\left(e\right)$ on the length $\left|E(P_{e})\right|$ of a pure $u,u^{\prime }$-path $P_{e}$. (For a notational convenience, set $\ell_{\mathrm{LB}}\left(e\right):=0$, $\ell_{\mathrm{UB}}\left(e\right):=1$, $e\in E_{\left(0/1\right)}$ and $\ell_{\mathrm{LB}}\left(e\right):=1$, $\ell_{\mathrm{UB}}\left(e\right):=1$, $e\in E_{\left(=1\right)}$. )
  a lower bound ${\mathrm{bl}}_{\mathrm{LB}}\left(e\right)$ and an upper bound ${\mathrm{bl}}_{\mathrm{UB}}\left(e\right)$ on the number of leaf paths $Q_{v}$ attached to at internal vertices *v* of a pure $u,u^{\prime }$-path $P_{e}$.
  a lower bound ${\mathrm{ch}}_{\mathrm{LB}}\left(e\right)$ and an upper bound ${\mathrm{ch}}_{\mathrm{UB}}\left(e\right)$ on the maximum length $\left|E(Q_{v})\right|$ of a leaf path $Q_{v}$ attached at an internal vertex $v\in V\left(P_{e}\right)\backslash \left\{u,u^{\prime }\right\}$ of a pure $u,u^{\prime }$-path $P_{e}$.
- For each vertex $v\in V_{C}$,
  a lower bound ${\mathrm{ch}}_{\mathrm{LB}}\left(e\right)$ and an upper bound ${\mathrm{ch}}_{\mathrm{UB}}\left(e\right)$ on the number of leaf paths $Q_{v}$ attached to *v*, where $0\leq {\mathrm{ch}}_{\mathrm{LB}}\left(e\right)\leq {\mathrm{ch}}_{\mathrm{UB}}\left(e\right)\leq 1$.
  a lower bound ${\mathrm{ch}}_{\mathrm{LB}}\left(v\right)$ and an upper bound ${\mathrm{ch}}_{\mathrm{UB}}\left(v\right)$ on the length $\left|E(Q_{v})\right|$ of a leaf path $Q_{v}$ attached to *v*.
- For each edge $e=uu^{\prime }\in E_{C}$, a lower bound ${\mathrm{bd}}_{m,\mathrm{LB}}\left(e\right)$ and an upper bound ${\mathrm{bd}}_{m,\mathrm{UB}}\left(e\right)$ on the number of edges with bond-multiplicity m∈[2,3] in $u,u^{\prime }$-path $P_{e}$, where we regard $P_{e}$, $e\in E_{\left(0/1\right)}\cup E_{\left(=1\right)}$ as single edge *e*.

We call a graph $H^{*}$ that satisfies an interior-specification $\sigma_{\mathrm{int}}$ a *$\sigma_{\mathrm{int}}$-extension of $G_{C}$*, where the bond-multiplicity of each edge has been determined.

Table 1 shows an example of an interior-specification $\sigma_{\mathrm{int}}$ to the seed graph $G_{C}$ in Figure 3.

**Figure 6:**
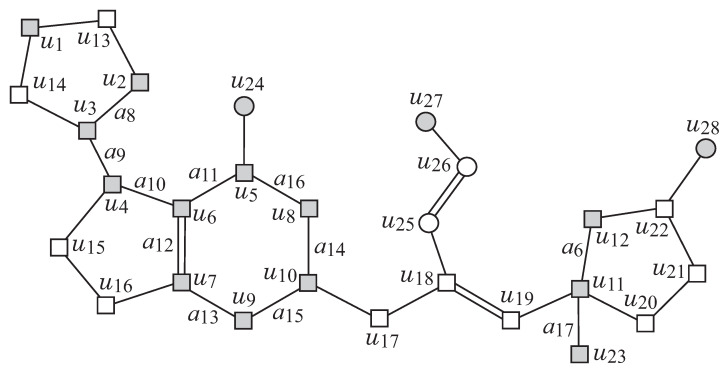
An illustration of a graph $H^{*}$ that is obtained from the seed graph $G_{C}$ in Figure 3 under the interior-specification $\sigma_{\mathrm{int}}$ in Table 1, where the vertices newly introduced by pure paths $P_{a_{i}}$ and leaf paths $Q_{v_{i}}$ are depicted with white squares and circles, respectively.

Figure 6 illustrates an example of an $\sigma_{\mathrm{int}}$-extension $H^{*}$ of seed graph $G_{C}$ in Figure 3 under the interior-specification $\sigma_{\mathrm{int}}$ in Table 1.

Chemical-Specification

Let $H^{*}$ be a graph that serves as the interior $H^{\mathrm{int}}$ of a target chemical graph *G*, where the bond-multiplicity of each edge in $H^{*}$ has be determined. Finally, we introduce a set of rules for constructing a target chemical graph *G* from $H^{*}$ by choosing a chemical element a∈Λ and assigning a ρ-fringe-tree ψ to each interior-vertex $v\in V^{\mathrm{int}}$. We introduce the following rules for specifying the size of *G*, a set of chemical rooted trees that are allowed to use as ρ-fringe-trees and lower and upper bounds on the frequency of a chemical element, a chemical symbol, and an edge-configuration, where we call the set of prescribed constants a *chemical specification* $\sigma_{\mathrm{ce}}$:

- Lower and upper bounds $n_{\mathrm{LB}},n^{*}\in Z_{+}$ on the number of vertices in *G*, where $n_{\mathrm{LB}}^{\mathrm{int}}\leq n_{\mathrm{LB}}\leq n^{*}$.
- Subsets $F\left(v\right)\subseteq F\left(D_{\pi }\right),v\in V_{C}$ and $F_{E}\subseteq F\left(D_{\pi }\right)$ of chemical rooted trees with height at most ρ, where we require that every ρ-fringe-tree $T_{v}$ rooted at a vertex $v\in V_{C}$ (resp., at an internal vertex *v* not in $V_{C}$) in *G* belongs to F(v) (resp., $F_{E}$). Let $F^{*}:=F_{E}\cup {⋃}_{v\in V_{C}}F\left(v\right)$ and $\Lambda^{\mathrm{ex}}$ denote the set of chemical elements assigned to non-root vertices over all chemical rooted trees in $F^{*}$.
- A subset $\Lambda^{\mathrm{int}}\subseteq \Lambda^{\mathrm{int}}\left(D_{\pi }\right)$, where we require that every chemical element α(v) assigned to an interior-vertex *v* in *G* belongs to $\Lambda^{\mathrm{int}}$. Let $\Lambda :=\Lambda^{\mathrm{int}}\cup \Lambda^{\mathrm{ex}}$ and ${\mathrm{na}}_{a}\left(G\right)$ (resp., ${\mathrm{na}}_{a}^{\mathrm{int}}\left(G\right)$ and ${\mathrm{na}}_{a}^{\mathrm{ex}}\left(G\right)$) denote the number of vertices (resp., interior-vertices and exterior-vertices) *v* such that α(v)=a in *G*.
- A set $\Lambda_{\mathrm{dg}}^{\mathrm{int}}\subseteq \Lambda \times \left[1,4\right]$ of chemical symbols and a set $\Gamma^{\mathrm{int}}\subseteq \Gamma^{\mathrm{int}}\left(D_{\pi }\right)$ of edge-configurations (μ,ξ,m) with μ≤ξ, where we require that the edge-configuration ec(e) of an interior-edge *e* in *G* belongs to $\Gamma^{\mathrm{int}}$. We do not distinguish (μ,ξ,m) and (ξ,μ,m).
- Define $\Gamma_{\mathrm{ac}}^{\mathrm{int}}$ to be the set of adjacency-configurations such that $\Gamma_{\mathrm{ac}}^{\mathrm{int}}:=\left\{\left(a,b,m\right)|\left(ad,bd^{\prime },m\right)\in \Gamma^{\mathrm{int}}\right\}$. Let ${\mathrm{ac}}_{\nu }^{\mathrm{int}}\left(G\right),\nu \in \Gamma_{\mathrm{ac}}^{\mathrm{int}}$ denote the number of interior-edges *e* such that ac(e)=ν in *G*.
- Subsets $\Lambda^{*}\left(v\right)\subseteq \left\{a\in \Lambda^{\mathrm{int}}|\mathrm{val}\left(a\right)\geq 2\right\}$, $v\in V_{C}$, we require that every chemical element α(v) assigned to a vertex $v\in V_{C}$ in the seed graph belongs to $\Lambda^{*}\left(v\right)$.
- Lower and upper bound functions ${\mathrm{na}}_{\mathrm{LB}},{\mathrm{na}}_{\mathrm{UB}}:\Lambda \to \left[1,n^{*}\right]$ and ${\mathrm{na}}_{\mathrm{LB}}^{\mathrm{int}},{\mathrm{na}}_{\mathrm{UB}}^{\mathrm{int}}:\Lambda^{t}\to \left[1,n^{*}\right]$ on the number of interior-vertices *v* such that α(v)=a in *G*.
- Lower and upper bound functions ${\mathrm{ns}}_{\mathrm{LB}}^{\mathrm{int}},{\mathrm{ns}}_{\mathrm{UB}}^{\mathrm{int}}:\Lambda_{\mathrm{dg}}^{\mathrm{int}}\to \left[1,n^{*}\right]$ on the number of interior-vertices *v* such that cs(v)=μ in *G*.
- Lower and upper bound functions ${\mathrm{ac}}_{\mathrm{LB}}^{\mathrm{int}},{\mathrm{ac}}_{\mathrm{UB}}^{\mathrm{int}}:\Gamma_{\mathrm{ac}}^{\mathrm{int}}\to Z_{+}$ on the number of interior-edges *e* such that ac(e)=ν in *G*.
- Lower and upper bound functions ${\mathrm{ec}}_{\mathrm{LB}}^{\mathrm{int}},{\mathrm{ec}}_{\mathrm{UB}}^{\mathrm{int}}:\Gamma^{\mathrm{int}}\to Z_{+}$ on the number of interior-edges *e* such that ec(e)=γ in *G*.

We call a chemical graph *G* that satisfies a chemical specification $\sigma_{\mathrm{ce}}$ a *$\left(\sigma_{\mathrm{int}},\sigma_{\mathrm{ce}}\right)$-extension of $G_{C}$*, and denote by $G(G_{C},\sigma_{\mathrm{int}},\sigma_{\mathrm{ce}})$ the set of all $\left(\sigma_{\mathrm{int}},\sigma_{\mathrm{ce}}\right)$-extensions of $G_{C}$.

Table 2 shows an example of a chemical-specification $\sigma_{\mathrm{ce}}$ to the seed graph $G_{C}$ in Figure 3.

Figure 2 illustrates an example of a $\left(\sigma_{\mathrm{int}},\sigma_{\mathrm{ce}}\right)$-extension of $G_{C}$ obtained from the $\sigma_{\mathrm{int}}$-extension $H^{*}$ in Figure 6 under the chemical-specification $\sigma_{\mathrm{ce}}$ in Table 2.

Our specification of topological substructures is similar to that proposed by Akutsu and Nagamochi [27], wherein a target chemical graph is restricted to ρ-lean cyclic graphs and prescribed substructures cannot be specified in the acyclic part. In our new method, a chemical graph with any structure can be handled and substructures in the acyclic part can be fixed.

### 2.3. Examples of Specification

We here present some cases where a target specification $\left(G_{C},\sigma_{\mathrm{int}},\sigma_{\mathrm{ce}}\right)$ can be chosen based on a set $G^{*}$ of given chemical graphs with a similar structure so that $G^{*}$ becomes a subset of $G(G_{C},\sigma_{\mathrm{int}},\sigma_{\mathrm{ce}})$. In such a case, every target chemical graph in $G(G_{C},\sigma_{\mathrm{int}},\sigma_{\mathrm{ce}})$ possesses a common structure over the given set $G^{*}$.

**Figure 7:**
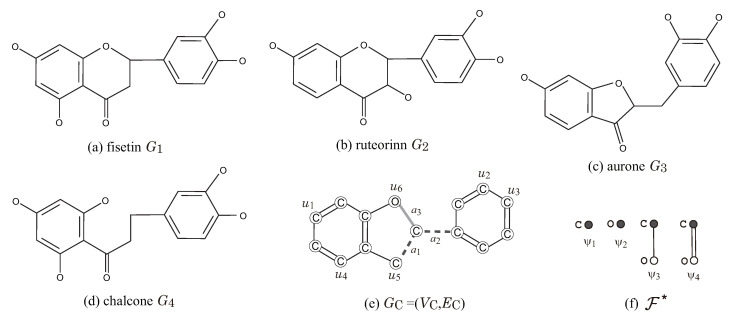
Illustration of a set $G^{*}=\left\{G_{1},G_{2},G_{3},G_{4}\right\}$ of four flavonoids, a seed graph $G_{C}$, and a set $F^{*}=\left\{\psi_{1},\psi_{2},\psi_{3},\psi_{4}\right\}$ of chemical rooted trees for ρ=2: (**a**) fisetin $G_{1}$; (**b**) ruteorinn $G_{2}$; (**c**) aurone $G_{3}$; (**d**) chalcone $G_{4}$; (**e**) $G_{C}=\left(V_{C},E_{C}\right)$; (**f**) $F^{*}=F_{E}\cup {⋃}_{v\in V_{C}}F\left(v\right)$.

Figure 7 illustrates a set $G^{*}$ of four flavonoids and a seed graph $G_{C}$ for ρ=2 so that $G^{*}\subseteq G\left(G_{C},\sigma_{\mathrm{int}},\sigma_{\mathrm{ce}}\right)$ for a choice of an interior-specification $\sigma_{\mathrm{int}}$ and a chemical-specification $\sigma_{\mathrm{ce}}$. Let Λ:={C,O}. In the seed graph $G_{C}=\left(V_{C},E_{C}\right)$, we set $E_{\left(\geq 1\right)}:=\left\{a_{1},a_{2}\right\}$, $E_{\left(0/1\right)}:=\left\{a_{3}\right\}$, and $E_{\left(=1\right)}:=E_{C}\backslash \left\{a_{1},a_{2},a_{3}\right\}$ and predetermine the chemical element α(u) for each vertex $u\in V_{C}$ and the bond-multiplicity β(e) for each edge $e\in E_{\left(=1\right)}$ as in Figure 7e, i.e., $\Lambda^{*}\left(u\right):=\left\{a\right\}$ for a=α(u) and ${\mathrm{bd}}_{m,\mathrm{LB}}\left(e\right):=1$ for m=β(e). Figure 7f illustrates a set $F^{*}$ of chemical rooted trees for the 2-fringe-trees in a target chemical graph. For vertices in $G_{C}$, we set ${\mathrm{ch}}_{\mathrm{UB}}\left(u\right):=0,u\in V_{C}$, $F\left(u_{i}\right):=\left\{\psi_{3}\right\},i\in \left[1,3\right]$, $F\left(u_{4}\right):=\left\{\psi_{1},\psi_{3}\right\}$, $F\left(u_{5}\right):=\left\{\psi_{4}\right\}$, $F\left(u_{6}\right):=\left\{\psi_{2}\right\}$, and $F\left(u\right):=\left\{\psi_{1}\right\},u\in V_{C}\backslash \left\{u_{1},u_{2},\ldots ,u_{6}\right\}$. For edges $a_{i}\in E_{\left(\geq 1\right)},i=1,2$, we set $\ell_{\mathrm{UB}}\left(a_{i}\right):=2,{\mathrm{ch}}_{\mathrm{UB}}\left(a_{i}\right):=0$ and $F_{E}:=\left\{\psi_{1},\psi_{2}\right\}$, where a pure path $P_{a_{i}}$ may be introduced in a target chemical graph. We see that every given chemical graph $G_{i}\in G^{*}$ belongs to $G(G_{C},\sigma_{\mathrm{int}},\sigma_{\mathrm{ce}})$ by setting the other specification in $\sigma_{\mathrm{int}}$ and $\sigma_{\mathrm{ce}}$ adequately.

**Figure 8:**
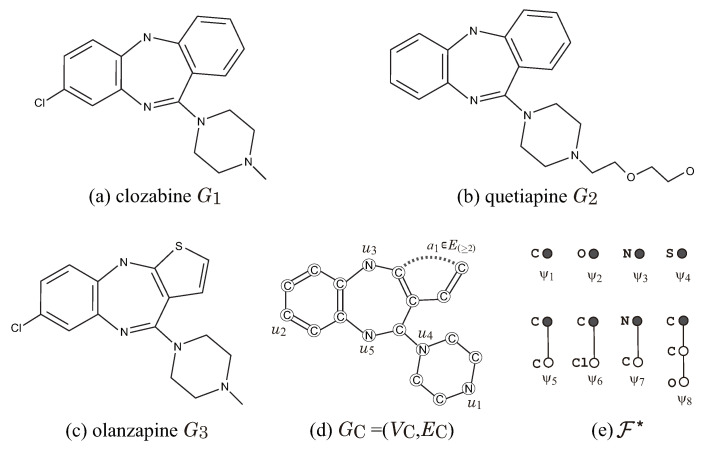
Illustration of a set $G^{*}=\left\{G_{1},G_{2},G_{3}\right\}$ of three dibenzodiazepine atypical antipsychotics, a seed graph $G_{C}$ and a set $F^{*}=\left\{\psi_{1},\psi_{2},\ldots ,\psi_{8}\right\}$ of chemical rooted trees for ρ=2: (**a**) clozabine $G_{1}$; (**b**) quetiapine $G_{2}$; (**c**) olanzapine $G_{3}$; (**d**) $G_{C}=\left(V_{C},E_{C}\right)$; (**e**) $F^{*}=F_{E}\cup {⋃}_{v\in V_{C}}F\left(v\right)$.

Figure 8 illustrates a set $G^{*}$ of three dibenzodiazepine atypical antipsychotics, and a seed graph $G_{C}$ for ρ=2 so that $G^{*}\subseteq G\left(G_{C},\sigma_{\mathrm{int}},\sigma_{\mathrm{ce}}\right)$ for a choice of an interior-specification $\sigma_{\mathrm{int}}$ and a chemical-specification $\sigma_{\mathrm{ce}}$. Let Λ:={C,O,N,S,Cl}. In the seed graph $G_{C}=\left(V_{C},E_{C}\right)$, we set $E_{\left(\geq 2\right)}:=\left\{a_{1}\right\}$ and $E_{\left(=1\right)}:=E_{C}\backslash \left\{a_{1}\right\}$ and predetermine the chemical element α(u) for each vertex $u\in V_{C}$ and the bond-multiplicity β(e) for each edge $e\in E_{\left(=1\right)}$ as in Figure 8d. Figure 8e illustrates a set $F^{*}$ of chemical rooted trees for the 2-fringe-trees in a target chemical graph. For vertices in $G_{C}$, we set ${\mathrm{ch}}_{\mathrm{UB}}\left(u\right):=0,u\in V_{C}\backslash \left\{u_{2}\right\}$, ${\mathrm{ch}}_{\mathrm{LB}}\left(u_{2}\right):=0$, ${\mathrm{ch}}_{\mathrm{UB}}\left(u_{2}\right):=4$, $F\left(u_{1}\right):=\left\{\psi_{3},\psi_{7}\right\}$, $F\left(u_{2}\right):=\left\{\psi_{1},\psi_{6}\right\}$, $F\left(u_{i}\right):=\left\{\psi_{3}\right\},i\in \left[3,5\right]$, and $F\left(u\right):=\left\{\psi_{1}\right\},u\in V_{C}\backslash \left\{u_{1},u_{2},\ldots ,u_{6}\right\}$, where a leaf path $Q_{u_{2}}$ may be introduced in a target chemical graph. For edge $a_{1}\in E_{\left(\geq 2\right)}$, we set $\ell_{\mathrm{UB}}\left(a_{1}\right):=3,{\mathrm{ch}}_{\mathrm{UB}}\left(a_{1}\right):=0$ and $F_{E}:=\left\{\psi_{1},\psi_{2},\psi_{4},\psi_{8}\right\}$. We see that every given chemical graph $G_{i}\in G^{*}$ belongs to $G(G_{C},\sigma_{\mathrm{int}},\sigma_{\mathrm{ce}})$ by setting the other specification in $\sigma_{\mathrm{int}}$ and $\sigma_{\mathrm{ce}}$ adequately.

## 3. Results

We implemented our method of Stages 1 to 5 for inferring chemical graphs under a given target specification and conducted experiments to evaluate the computational efficiency. We executed the experiments on a PC with Processor: 3.0 GHz Core i7-9700 (3.0 GHz) Memory: 16 GB RAM DDR4. We used ChemDoodle version 10.2.0 for constructing 2D drawings of chemical graphs.

To conduct experiments for Stages 1 to 5, we selected six chemical properties π: octanol/water partition coefficient (K_OW_), boiling point (B_P_), melting point (M_P_), flash point (closed cup) (F_P_), lipophylicity (L_P_), solubility (S_L_) provided by HSDB from PubChem [29] for K_OW_, B_P_, M_P_, and F_P_, figshare [30] for L_P_ and MoleculeNet [31] for S_L_. 

**Results on Phase 1.**

We implemented Stages 1, 2, and 3 in Phase 1 as follows.

**Stage 1.** We set a graph class G to be the set of all chemical graphs with any graph structure, and set a branch-parameter ρ to be 2. For each property π ∈ {K_OW_, B_P_, M_P_, F_P_, L_P_, S_L_}, we first select a set Λ of chemical elements and then collect a data set $D_{\pi }$ on chemical graphs over the set Λ of chemical elements. To construct the data set $D_{\pi }$, we eliminated chemical compounds that have at most three carbon atoms or contain a charged element such as $N^{+}$ or an element a∈Λ whose valence is different from our setting of valence function val.

Table 3 shows the size and range of data sets that we prepared for each chemical property in Stage 1, where we denote the following:

Λ: the set of selected chemical elements (hydrogen atoms are added at the final stage);
$|D_{\pi }|$: the size of data set $D_{\pi }$ over Λ for property π;
$|\Gamma^{\mathrm{int}}\left(D_{\pi }\right)|$: the number of different edge-configurations of interior-edges over the compounds in $D_{\pi }$;
$\left|F(D_{\pi })\right|$: the number of non-isomorphic chemical rooted trees in the set of all 2-fringe-trees in the compounds in $D_{\pi }$;
$\left[\underline{n},\bar{n}\right]$: the minimum and maximum values of n(G) over the compounds *G* in $D_{\pi }$; and
$\left[\underline{a},\bar{a}\right]$: the minimum and maximum values of a(G) in π over compounds *G* in $D_{\pi }$.

**Stage 2.** We used the new feature function that consists of the descriptors such as fringe-configuration defined in Section 2.1 and let $f_{\mathrm{fc}}$ denote the feature function.

**Stage 3.** Let $\eta :R^{K}\to R$ be a prediction function to a property function a:D→R with a feature function $f:D\to R^{K}$ for a data set *D* of chemical graphs. We define the coefficient of determination $R^{2}\left(f,\eta ,D\right)$ of a prediction function η over a data set *D* to be

$$R^{2}\left(f,\eta ,D\right)≜1-\frac{\sum_{G\in D}{\left(a\left(G\right)-\eta \left(f\left(G\right)\right)\right)}^{2}}{\sum_{G\in D}{\left(a\left(G\right)-\tilde{a}\right)}^{2}}for\tilde{a}=\frac{1}{\left|D\right|}\sum_{G\in D}a\left(G\right).$$

To conduct an experiment in Stage 3, we first constructed ten architectures $A_{j}$, j∈[1,10] with one or two hidden layers. For each pair $\left(\pi ,A_{j}\right)$ of a property π ∈ {K_OW_, B_P_, M_P_, F_P_, L_P_, S_L_}, and an architecture $A_{j}$, j∈[1,10], we constructed five prediction functions in order to evaluate the performance with cross-validation as follows. Partition data set $D_{\pi }$ into five subsets $D_{\pi }^{\left(i\right)}$, i∈[1,5] randomly and for each set $D_{\pi }\backslash D_{\pi }^{\left(i\right)}$ construct an ANN N(j,i) and its prediction function $\eta_{N(j,i)}$ using the feature function $f_{\mathrm{fc}}$. We used scikit-learn version 0.23.2 with Python 3.8.5, MLPRegressor and ReLU activation function to construct each ANN N(j,i). We evaluated the resulting prediction function $\eta_{N(j,i)}$ with the coefficient $R^{2}\left(f_{\mathrm{fc}},\eta_{N(j,i)},D_{\pi }^{\left(i\right)}\right)$ of determination for the test set $D_{\pi }^{\left(i\right)}$. For each property π, let t-$R_{\mathrm{cv}}^{2}\left(j\right)$ denote the average of $R^{2}\left(f_{\mathrm{fc}},\eta_{N(j,i)},D_{\pi }^{\left(i\right)}\right)$ over all i∈[1,5] in the cross-validation to an architecture $A_{j}$.

Table 4 shows the results on Stages 2 and 3, where we denote the following.

- Λ: the set of selected chemical elements (hydrogen atoms are added at the final stage);
- L-time: the average time (s) to construct an ANN over all 10×5=50 ANNs;
- t-$R_{\mathrm{cv}}^{2}$ (best): the best value of t-$R_{\mathrm{cv}}^{2}\left(j\right)$ over all architectures $A_{j}$, j∈[1,10];
- t-$R_{\max}^{2}$: the maximum of $R^{2}\left(f_{\mathrm{fc}},\eta_{N(j,i)},D_{\pi }^{\left(i\right)}\right)$ over all j∈[1,10],i∈[1,5]; and
- Arch.: The architecture $A_{j}$, j∈[1,10] that attains t-$R_{\max}^{2}$. An architecture (K,p,1) (resp., $\left(K,p_{1},p_{2},1\right)$) consists of an input layer with *K* nodes, a hidden layer with *p* nodes (resp., two hidden layers with $p_{1}$ and $p_{2}$ nodes, respectively), and an output layer with a single node, where *K* is equal to the number of descriptors in the feature vector.

From Table 4, we see that the execution of Stage 3 was considerably successful, where most of t-$R_{\max}^{2}$ are around 0.85 to 0.95 for all six chemical properties.

**An Additional Experiment in Stage 3.** We conducted an additional experiment to compare our new feature function $f_{\mathrm{fc}}$ with the feature function $f_{\mathrm{ec}}$ based edge-configuration in the previous method [27] designed with the same framework. Note that the previous feature vector $f_{\mathrm{ec}}\left(G\right)$ can be defined only for a cyclic graph *G*, whereas our feature vector $f_{\mathrm{fc}}\left(G\right)$ is defined for an arbitrary graph *G*. For each property π ∈ {K_OW_, B_P_, M_P_, F_P_, L_P_, S_L_}, we set a set Λ of chemical elements to be {C,O,N,S,Cl} and then collect a data set ${\tilde{D}}_{\pi }$ of chemical cyclic graphs from the data set $D_{\pi }$ of all chemical graphs over the set Λ of chemical elements in the previous experiment. For each of the feature functions $f_{\mathrm{ec}}$ and $f_{\mathrm{fc}}$, we constructed five prediction functions with the same set of ten architectures $A_{j}$, j∈[1,10] and the data set ${\tilde{D}}_{\pi }$ of chemical cyclic graphs in the same manner of the previous experiment.

Table 5 shows the results of this experiment, where the table also includes the result of prediction functions by $f_{\mathrm{fc}}$ in the set $D_{\pi }$ of all chemical graphs. In the table, we denote the following:

- $|{\tilde{D}}_{\pi }|$, $|D_{\pi }|$: the size of data set ${\tilde{D}}_{\pi }$ of cyclic graphs (resp., $D_{\pi }$ of all chemical graphs) for property π;
- t-$R_{\mathrm{cv}}^{2}$ (ave.): the average of $R^{2}\left(f,\eta_{N(j,i)},D^{\left(i\right)}\right)$ over all j∈[1,10],i∈[1,5] for $f=f_{\mathrm{ec}},f_{\mathrm{fc}}$ and $D={\tilde{D}}_{\pi },D_{\pi }$; and
- t-$R_{\mathrm{cv}}^{2}$ (best): $\max_{j\in [1,10]}\{$the average of $R^{2}\left(f_{\mathrm{fc}},\eta_{N(j,i)},D_{\pi }^{\left(i\right)}\right)$ over all i∈[1,5]}.

From Table 5, we see that the score of R${}^{2}$ of the prediction function by $f_{\mathrm{fc}}$ in chemical cyclic graphs (resp., in all chemical graphs) is improved from that by $f_{\mathrm{ec}}$ for properties M_P_ and F_P_ (resp., B_P_, M_P_, and F_P_). Recall that our new feature function $f_{\mathrm{fc}}$ can be defined for arbitrary graphs and we can select a larger data set than that by $f_{\mathrm{ec}}$ in a learning stage. This advantage is observed in the experiment. We guess that the better prediction function for B_P_ (resp., F_P_) is obtained by using $f_{\mathrm{fc}}$ because the size of data set becomes considerably larger from $|{\tilde{D}}_{\pi }|=224$ to $|D_{\pi }|=425$ (resp., from $|{\tilde{D}}_{\pi }|=218$ to $|D_{\pi }|=399$).

**Results on Phase 2.**

**Figure 9:**
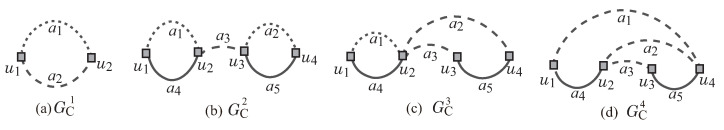
An illustration of seed graphs: (**a**) A monocyclic graph $G_{C}^{1}$; (**b**) A rank-2 cyclic graph $G_{C}^{2}$ with two vertex-disjoint cycles; (**c**) A rank-2 cyclic graph $G_{C}^{3}$ with two disjoint cycles sharing a vertex; (**d**) A rank-2 cyclic graph $G_{C}^{4}$ with three cycles.

We prepared the following instances (a–d) for conducting experiments of Stages 4 and 5 in Phase 2.

(a) $I_{a}=\left(G_{C},\sigma_{\mathrm{int}},\sigma_{\mathrm{ce}}\right)$: The instance used in Section 2.2 to explain the target specification.
(b) $I_{b,i}=\left(G_{C}^{i},\sigma_{\mathrm{int}}^{i},\sigma_{\mathrm{ce}}^{i}\right)$, i=1,2,3,4: An instance for inferring chemical graphs with rank at most 2. In the four instances $I_{b,i}$, i=1,2,3,4, the following specifications in $\left(\sigma_{\mathrm{int}},\sigma_{\mathrm{ce}}\right)$ are common.
  Set Λ:={C,N,O}, set $\Lambda_{\mathrm{dg}}^{\mathrm{int}}$ to be the set of all possible symbols in Λ×[1,4], and set $\Gamma^{\mathrm{int}}$ to be the set of all possible edge-configurations. Set $\Lambda^{*}\left(v\right):=\Lambda$, $v\in V_{C}$.
  The lower bounds $\ell_{\mathrm{LB}}$, ${\mathrm{bl}}_{\mathrm{LB}}$, ${\mathrm{ch}}_{\mathrm{LB}}$, ${\mathrm{bd}}_{2,\mathrm{LB}}$, ${\mathrm{bd}}_{3,\mathrm{LB}}$, ${\mathrm{na}}_{\mathrm{LB}}$, ${\mathrm{na}}_{\mathrm{LB}}^{\mathrm{int}}$, ${\mathrm{ns}}_{\mathrm{LB}}^{\mathrm{int}}$, ${\mathrm{ac}}_{\mathrm{LB}}^{\mathrm{int}}$, ${\mathrm{ec}}_{\mathrm{LB}}^{\mathrm{int}}$ are all set to be 0.
  The upper bounds $\ell_{\mathrm{UB}}$, ${\mathrm{bl}}_{\mathrm{UB}}$, ${\mathrm{ch}}_{\mathrm{UB}}$, ${\mathrm{bd}}_{2,\mathrm{UB}}$, ${\mathrm{bd}}_{3,\mathrm{UB}}$, ${\mathrm{na}}_{\mathrm{UB}}$, ${\mathrm{na}}_{\mathrm{UB}}^{\mathrm{int}}$, ${\mathrm{ns}}_{\mathrm{UB}}^{\mathrm{int}}$, ${\mathrm{ac}}_{\mathrm{UB}}^{\mathrm{int}}$, ${\mathrm{ec}}_{\mathrm{UB}}^{\mathrm{int}}$ are all set to be an upper bound $n^{*}$ on $n(G^{*})$.
  For each property π, let $F(D_{\pi })$ denote the set of 2-fringe-trees in the compounds in $D_{\pi }$, and select a subset $F_{\pi }^{i}\subseteq F\left(D_{\pi }\right)$ with $|F_{\pi }^{i}|=45-5i$, i∈[1,5]. For each instance $I_{b,i}$, set $F_{E}:=F\left(v\right):=F_{\pi }^{i}$, $v\in V_{C}$.
  Instance $I_{b,1}$ is given by the rank-1 seed graph $G_{C}^{1}$ in Figure 9a and Instances $I_{b,i}$, i=2,3,4 are given by the rank-2 seed graph $G_{C}^{i}$, i=2,3,4 in Figure 9b–d.
  (i) For instance $I_{b,1}$, select as a seed graph the monocyclic graph $G_{C}^{1}=\left(V_{C},E_{C}=E_{\left(\geq 2\right)}\cup E_{\left(\geq 1\right)}\right)$ in Figure 9a, where $V_{C}=\left\{u_{1},u_{2}\right\}$, $E_{\left(\geq 2\right)}=\left\{a_{1}\right\}$ and $E_{\left(\geq 1\right)}=\left\{a_{2}\right\}$. Set $n_{\mathrm{LB}}^{\mathrm{int}}:=0,n_{\mathrm{UB}}^{\mathrm{int}}:=12$ and $n_{\mathrm{LB}}:=n^{*}:=38$. We include a linear constraint $\ell \left(a_{1}\right)\leq \ell \left(a_{2}\right)$ as part of the side constraint.
  (ii) For instance $I_{b,2}$, select as a seed graph the graph $G_{C}^{2}=\left(V_{C},E_{C}=E_{\left(\geq 2\right)}\cup E_{\left(\geq 1\right)}\cup E_{\left(=1\right)}\right)$ in Figure 9b, where $V_{C}=\left\{u_{1},u_{2},u_{3},u_{4}\right\}$, $E_{\left(\geq 2\right)}=\left\{a_{1},a_{2}\right\}$, $E_{\left(\geq 1\right)}=\left\{a_{3}\right\}$ and $E_{\left(=1\right)}=\left\{a_{4},a_{5}\right\}$. Set $n_{\mathrm{LB}}^{\mathrm{int}}:=n_{\mathrm{UB}}^{\mathrm{int}}:=30$ and $n_{\mathrm{LB}}:=n^{*}:=50$. We include a linear constraint $\ell \left(a_{1}\right)\leq \ell \left(a_{2}\right)$.
  (iii) For instance $I_{b,3}$, select as a seed graph the graph $G_{C}^{3}=\left(V_{C},E_{C}=E_{\left(\geq 2\right)}\cup E_{\left(\geq 1\right)}\cup E_{\left(=1\right)}\right)$ in Figure 9c, where $V_{C}=\left\{u_{1},u_{2},u_{3},u_{4}\right\}$, $E_{\left(\geq 2\right)}=\left\{a_{1}\right\}$, $E_{\left(\geq 1\right)}=\left\{a_{2},a_{3}\right\}$ and $E_{\left(=1\right)}=\left\{a_{4},a_{5}\right\}$. Set $n_{\mathrm{LB}}^{\mathrm{int}}:=n_{\mathrm{UB}}^{\mathrm{int}}:=30$ and $n_{\mathrm{LB}}:=n^{*}:=50$. We include linear constraints $\ell \left(a_{1}\right)\leq \ell \left(a_{2}\right)+\ell \left(a_{3}\right)$ and $\ell \left(a_{2}\right)\leq \ell \left(a_{3}\right)$.
  (iv) For instance $I_{b,4}$, select as a seed graph the graph $G_{C}^{4}=\left(V_{C},E_{C}=E_{\left(\geq 2\right)}\cup E_{\left(\geq 1\right)}\cup E_{\left(=1\right)}\right)$ in Figure 9d, where $V_{C}=\left\{u_{1},u_{2},u_{3},u_{4}\right\}$, $E_{\left(\geq 1\right)}=\left\{a_{1},a_{2},a_{3}\right\}$ and $E_{\left(=1\right)}=\left\{a_{4},a_{5}\right\}$. Set $n_{\mathrm{LB}}^{\mathrm{int}}:=n_{\mathrm{UB}}^{\mathrm{int}}:=30$ and $n_{\mathrm{LB}}:=n^{*}:=50$. We include linear constraints $\ell \left(a_{2}\right)\leq \ell \left(a_{1}\right)+1$, $\ell \left(a_{2}\right)\leq \ell \left(a_{3}\right)+1$ and $\ell \left(a_{1}\right)\leq \ell \left(a_{3}\right)$.

**Figure 10:**
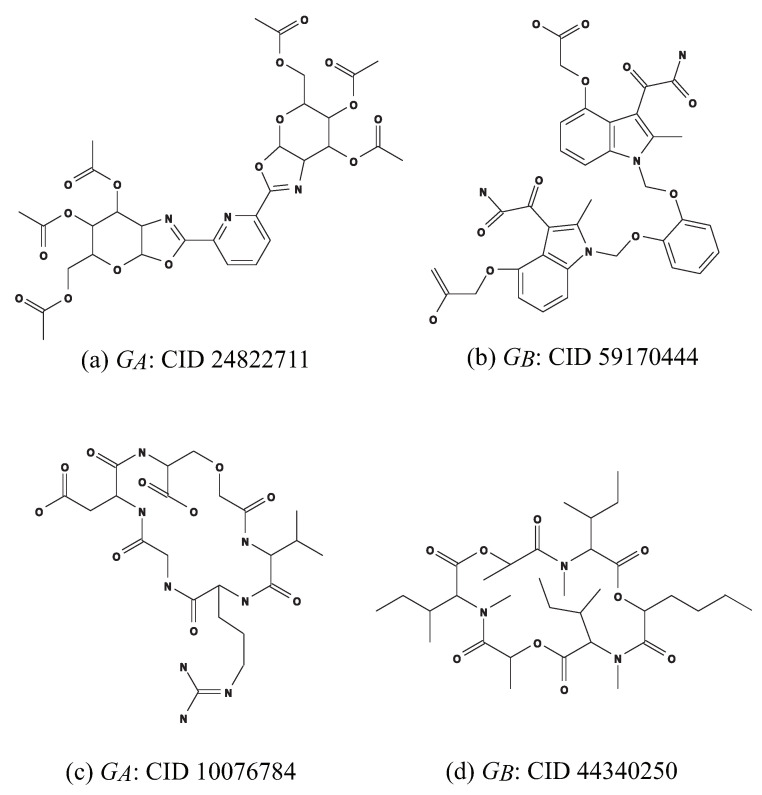
An illustration of chemical compounds for instances $I_{c}$ and $I_{d}$: (**a**) $G_{A}$: CID 24822711; (**b**) $G_{B}$: CID 59170444; (**c**) $G_{A}$: CID 10076784; (**d**) $G_{B}$: CID 44340250.

We define instances in (c) and (d) in order to find chemical graphs that have an intermediate structure of given two chemical cyclic graphs $G_{A}=\left(H_{A}=\left(V_{A},E_{A}\right),\alpha_{A},\beta_{A}\right)$ and $G_{B}=\left(H_{B}=\left(V_{B},E_{B}\right),\alpha_{B},\beta_{B}\right)$. Let $\Lambda_{A}^{\mathrm{int}}$ and $\Lambda_{\mathrm{dg},A}^{\mathrm{int}}$ denote the sets of chemical elements and chemical symbols of the interior-vertices in $G_{A}$, $\Gamma_{A}^{\mathrm{int}}$ denote the sets of edge-configurations of the interior-edges in $G_{A}$, and $F_{A}$ denote the set of 2-fringe-trees in $G_{A}$. Analogously define sets $\Lambda_{B}^{\mathrm{int}}$, $\Lambda_{\mathrm{dg},B}^{\mathrm{int}}$, $\Gamma_{B}^{\mathrm{int}}$, and $F_{B}$ in $G_{B}$.

(c) $I_{c}=\left(G_{C},\sigma_{\mathrm{int}},\sigma_{\mathrm{ce}}\right)$: An instance aimed to infer a chemical graph $G^{†}$ such that the core of $G^{†}$ is equal to the core of $G_{A}$ and the frequency of each edge-configuration in the non-core of $G^{†}$ is equal to that of $G_{B}$. We use chemical compounds CID 24822711 and CID 59170444 in Figure 10a,b for $G_{A}$ and $G_{B}$, respectively.
  Set a seed graph $G_{C}=\left(V_{C},E_{C}=E_{\left(=1\right)}\right)$ to be the core of $G_{A}$.
  Set Λ:={C,N,O}, and set $\Lambda_{\mathrm{dg}}^{\mathrm{int}}$ to be the set of all possible chemical symbols in Λ×[1,4].
  Set $\Gamma^{\mathrm{int}}:=\Gamma_{A}^{\mathrm{int}}\cup \Gamma_{B}^{\mathrm{int}}$ and $\Lambda^{*}\left(v\right):=\left\{\alpha_{A}\left(v\right)\right\}$, $v\in V_{C}$.
  Set $n_{\mathrm{LB}}^{\mathrm{int}}:=\min\left\{n^{\mathrm{int}}\left(G_{A}\right),n^{\mathrm{int}}\left(G_{B}\right)\right\}$, $n_{\mathrm{UB}}^{\mathrm{int}}:=\max\left\{n^{\mathrm{int}}\left(G_{A}\right),n^{\mathrm{int}}\left(G_{B}\right)\right\}$,
  $n_{\mathrm{LB}}:=\min\left\{n\left(G_{A}\right),n\left(G_{B}\right)\right\}-10$ and $n^{*}:=\max\left\{n\left(G_{A}\right),n\left(G_{B}\right)\right\}+5$.
  Set lower bounds $\ell_{\mathrm{LB}}$, ${\mathrm{bl}}_{\mathrm{LB}}$, ${\mathrm{ch}}_{\mathrm{LB}}$, ${\mathrm{bd}}_{2,\mathrm{LB}}$, ${\mathrm{bd}}_{3,\mathrm{LB}}$, ${\mathrm{na}}_{\mathrm{LB}}$, ${\mathrm{na}}_{\mathrm{LB}}^{\mathrm{int}}$, ${\mathrm{ns}}_{\mathrm{LB}}^{\mathrm{int}}$ and ${\mathrm{ac}}_{\mathrm{LB}}^{\mathrm{int}}$ to be 0.
  Set upper bounds $\ell_{\mathrm{UB}}$, ${\mathrm{bl}}_{\mathrm{UB}}$, ${\mathrm{ch}}_{\mathrm{UB}}$, ${\mathrm{bd}}_{2,\mathrm{UB}}$, ${\mathrm{bd}}_{3,\mathrm{UB}}$, ${\mathrm{na}}_{\mathrm{UB}}$, ${\mathrm{na}}_{\mathrm{UB}}^{\mathrm{int}}$, ${\mathrm{ns}}_{\mathrm{UB}}^{\mathrm{int}}$ and ${\mathrm{ac}}_{\mathrm{UB}}^{\mathrm{int}}$ to be $n^{*}$.
  Set ${\mathrm{ec}}_{\mathrm{LB}}^{\mathrm{int}}\left(\gamma \right)$ to be the number of core-edges in $G_{A}$ with $\gamma \in \Gamma^{\mathrm{int}}$ and ${\mathrm{ec}}_{\mathrm{UB}}^{\mathrm{int}}\left(\gamma \right)$ to be the number interior-edges in $G_{A}$ and $G_{B}$ with edge-configuration γ.
  Let $F_{B}^{\left(p\right)},p\in \left[1,2\right]$ denote the set of chemical rooted trees r-isomorphic *p*-fringe-trees in $G_{B}$.
  Set $F_{E}:=F\left(v\right):=F_{B}^{\left(1\right)}\cup F_{B}^{\left(2\right)}$, $v\in V_{C}$.
(d) $I_{d}=\left(G_{C}^{1},\sigma_{\mathrm{int}},\sigma_{\mathrm{ce}}\right)$: An instance aimed to infer a chemical monocyclic graph $G^{†}$ such that the frequency vector of edge-configurations in $G^{†}$ is a vector obtained by merging those of $G_{A}$ and $G_{B}$. We use chemical monocyclic compounds CID 10076784 and CID 44340250 in Figure 10c,d for $G_{A}$ and $G_{B}$, respectively. Set a seed graph to be the monocyclic seed graph $G_{C}^{1}=\left(V_{C},E_{C}=E_{\left(\geq 2\right)}\cup E_{\left(\geq 1\right)}\right)$ with $V_{C}=\left\{u_{1},u_{2}\right\}$, $E_{\left(\geq 2\right)}=\left\{a_{1}\right\}$ and $E_{\left(\geq 1\right)}=\left\{a_{2}\right\}$ in Figure 9a.
  Set Λ:={C,N,O}, $\Lambda_{\mathrm{dg}}^{\mathrm{int}}:=\Lambda_{\mathrm{dg},A}^{\mathrm{int}}\cup \Lambda_{\mathrm{dg},B}^{\mathrm{int}}$ and $\Gamma^{\mathrm{int}}:=\Gamma_{A}^{\mathrm{int}}\cup \Gamma_{B}^{\mathrm{int}}$.
  Set $n_{\mathrm{LB}}^{\mathrm{int}}:=\min\left\{n^{\mathrm{int}}\left(G_{A}\right),n^{\mathrm{int}}\left(G_{B}\right)\right\}$, $n_{\mathrm{UB}}^{\mathrm{int}}:=\max\left\{n^{\mathrm{int}}\left(G_{A}\right),n^{\mathrm{int}}\left(G_{B}\right)\right\}$,
  $n_{\mathrm{LB}}:=\min\left\{n\left(G_{A}\right),n\left(G_{B}\right)\right\}$ and $n^{*}:=\max\left\{n\left(G_{A}\right),n\left(G_{B}\right)\right\}$.
  Set lower bounds $\ell_{\mathrm{LB}}$, ${\mathrm{bl}}_{\mathrm{LB}}$, ${\mathrm{ch}}_{\mathrm{LB}}$, ${\mathrm{bd}}_{2,\mathrm{LB}}$, ${\mathrm{bd}}_{3,\mathrm{LB}}$, ${\mathrm{na}}_{\mathrm{LB}}$, ${\mathrm{na}}_{\mathrm{LB}}^{\mathrm{int}}$, ${\mathrm{ns}}_{\mathrm{LB}}^{\mathrm{int}}$ and ${\mathrm{ac}}_{\mathrm{LB}}^{\mathrm{int}}$ to be 0.
  Set upper bounds $\ell_{\mathrm{UB}}$, ${\mathrm{bl}}_{\mathrm{UB}}$, ${\mathrm{ch}}_{\mathrm{UB}}$, ${\mathrm{bd}}_{2,\mathrm{UB}}$, ${\mathrm{bd}}_{3,\mathrm{UB}}$, ${\mathrm{na}}_{\mathrm{UB}}$, ${\mathrm{na}}_{\mathrm{UB}}^{\mathrm{int}}$, ${\mathrm{ns}}_{\mathrm{UB}}^{\mathrm{int}}$ and ${\mathrm{ac}}_{\mathrm{UB}}^{\mathrm{int}}$ to be $n^{*}$.
  For each edge-configuration $\gamma \in \Gamma^{\mathrm{int}}$, let $x_{A}^{*}\left(\gamma^{\mathrm{int}}\right)$ (resp., $x_{B}^{*}\left(\gamma^{\mathrm{int}}\right)$) denote the number of interior-edges with γ in $G_{A}$ (resp., $G_{B}$), $\gamma \in \Gamma^{\mathrm{int}}$ and set
  $x_{\min}^{*}\left(\gamma \right):=\min\left\{x_{A}^{*}\left(\gamma \right),x_{B}^{*}\left(\gamma \right)\right\}$, $x_{\max}^{*}\left(\gamma \right):=\max\left\{x_{A}^{*}\left(\gamma \right),x_{B}^{*}\left(\gamma \right)\right\}$,
  ${\mathrm{ec}}_{\mathrm{LB}}^{\mathrm{int}}\left(\gamma \right):=\left\lfloor \left(3/4\right)x_{\min}^{*}\left(\gamma \right)+\left(1/4\right)x_{\max}^{*}\left(\gamma \right)\right\rfloor$ and
  ${\mathrm{ec}}_{\mathrm{UB}}^{\mathrm{int}}\left(\gamma \right):=\left\lceil \left(1/4\right)x_{\min}^{*}\left(\gamma \right)+\left(3/4\right)x_{\max}^{*}\left(\gamma \right)\right\rceil$.
  Set $F_{E}:=F\left(v\right):=F_{A}\cup F_{B}$, $v\in V_{C}$.

In Stage 5, before we formulate an MILP for inferring a target chemical graph $G^{†}$ for each instance *I*, we reduce the input layer of an ANN N constructed in Stage 3 so that the input layer consists of input nodes that correspond to the descriptors actually used in the specification $\left(G_{C},\sigma_{\mathrm{int}},\sigma_{\mathrm{ce}}\right)$ of the instance *I*, i.e., we remove any input nodes in N that represent the frequency of edge-configurations in $\Gamma^{\mathrm{int}}\left(D_{\pi }\right)$ and chemical rooted trees $\psi \in F(D_{\pi })$ not contained in the specification $\left(G_{C},\sigma_{\mathrm{int}},\sigma_{\mathrm{ce}}\right)$ of *I*. For example, there are $|F(D_{\pi })|=109$ chemical rooted trees in the set of 2-fringe-trees in the data set $D_{\pi }$ with π= K_OW_ in Table 3, and an ANN N constructed in Stage 3 contains 109 input nodes that correspond to the descriptors for the fringe-configuration. However, the set of input nodes for the fringe-configuration is reduced to a set of $|F^{*}|=40$ input nodes when we formulate an MILP for solving instance $I_{b,1}$, saving the number of integer variables.

Table 6 shows the features of the seven test instances, where we denote the following:

- Λ: the set of non-hydrogen chemical elements for inferring a target graph;
- $|\Gamma^{\mathrm{int}}|$: the number of different edge-configurations of interior-edges for inferring a target graph;
- $|F^{*}|$: the number of different chemical rooted trees in the set $F^{*}=F_{E}\cup {⋃}_{v\in V_{C}}F\left(v\right)$; and
- $\left[n_{\mathrm{LB}}^{\mathrm{int}},n_{\mathrm{UB}}^{\mathrm{int}}\right]$, $\left[n_{\mathrm{LB}},n^{*}\right]$: the lower and upper bounds on $n^{\mathrm{int}}\left(G^{†}\right)$ and $n(G^{†})$ for inferring a target graph $G^{†}$.

**Stage 4.** To solve an MILP in Stage 4, we used CPLEX version 12.10. Table 7, Table 8, Table 9, Table 10, Table 11 and Table 12 show the results on Stages 4 and 5, where we denote the following:

- $\left[\underline{a},\bar{a}\right]$: the minimum and maximum values of a(G) in π over compounds *G* in $D_{\pi }$ in Table 3;
- $\left[\underline{y},\bar{y}\right]$: $\underline{y}$ (resp., $\bar{y}$) denotes the minimum (resp., maximum) target value *y* with $\underline{a}\leq y\leq \bar{a}$ such that the MILP instance for the target value $y^{*}=y$ becomes feasible (i.e., admits a target chemical graph $G^{†}$). To determine the minimum and minimum target values $\underline{y}$ and $\bar{y}$, we solved many numbers of MILP instances. Note that the MILP instance may become infeasible for some value *y* within the range $\left[\underline{y},\bar{y}\right]$;
- $y^{*}$: a target value in $\left[\underline{y},\bar{y}\right]$ for a property π;
- #v: the number of variables in the MILP in Stage 4;
- #c: the number of constraints in the MILP in Stage 4;
- IP-time: the time (sec.) to solve the MILP in Stage 4;
- *n*: the number $n(G^{†})$ of non-hydrogen atoms in the chemical graph $G^{†}$ inferred in Stage 4; and
- $n^{\mathrm{int}}$: the number $n^{\mathrm{int}}\left(G^{†}\right)$ of interior-vertices in the chemical graph $G^{†}$ inferred in Stage 4.

**Figure 11:**
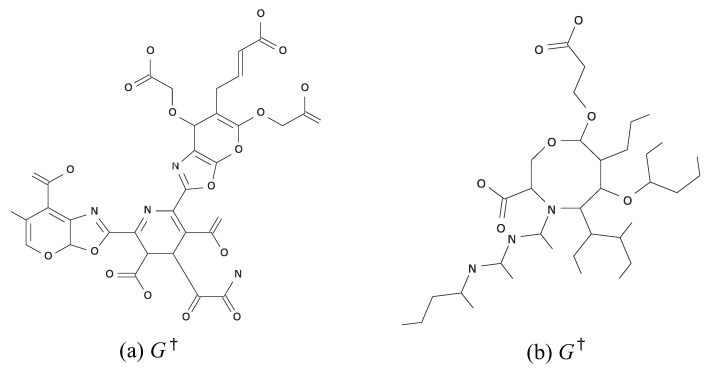
(**a**) $G^{†}$ inferred from $I_{c}$ with $y^{*}=3.0$ of K_OW_; (**b**) $G^{†}$ inferred from $I_{d}$ with $y^{*}=1.6$ of L_P_.

Figure 11a illustrates the chemical graph $G^{†}$ inferred from instance $I_{c}$ with $y^{*}=3.0$ of K_OW_ in Table 7.

Figure 11b illustrates the chemical graph $G^{†}$ inferred from instance $I_{d}$ with $y^{*}=1.6$ of L_P_ in Table 11.

The topological specification of instances $I_{a}$, $I_{c}$ and $I_{d}$ is more restricted than that of the other instances, and thereby the feasible target range $\left[\underline{y},\bar{y}\right]$ of $I_{a}$, $I_{c}$ and $I_{d}$ is rather narrower than the original range $\left[\underline{a},\bar{a}\right]$ for some property π. We see that the running time for solving an MILP instance with n=50 is 8.5 to 122 (s), which is much smaller than the running time of 61 to 12058 (s) to solve a similar set of MILP instances with n=50 in the experimental result for the previous method [28].

**Stage 5.** We computed chemical isomers $G^{*}$ of each target chemical graph $G^{†}$ inferred in Stage 4. We execute the algorithm for generating chemical isomers of $G^{†}$ up to 100 when the number of all chemical isomers exceeds 100. The algorithm can evaluate a lower bound on the total number of all chemical isomers $G^{†}$ without generating all of them.

Table 13 and Table 14 show the computational results of the experiment, where we denote the following:

- DP-time: the running time (s) to execute the dynamic programming algorithm in Stage 5 to compute a lower bound on the number of all chemical isomers $G^{*}$ of $G^{†}$ and generate all (or up to 100) chemical isomers $G^{*}$;
- G-LB: a lower bound on the number of all chemical isomers $G^{*}$ of $G^{†}$; and
- #G: the number of all (or up to 100) chemical isomers $G^{*}$ of $G^{†}$ generated in Stage 5.

From Table 13 and Table 14, we observe that the running time for generating up to 100 target chemical graphs in Stage 5 is not considerably larger than that in Stage 4.

## 4. Discussions and Conclusions

The framework of designing chemical graphs using ANNs and MILP has been proposed [23] as a basis of a total system of the QSAR and the inverse of QSAR, where the inverse of a prediction function produced by an ANN is solved by an MILP. The merit of the framework is that the inverse problem can be treated exactly as a mathematical problem, and an MILP instance with a moderate size can be efficiently solved with a fast MILP solver. On the other hand, the main technical concern in applying the framework is in defining a feature vector of a chemical graph in terms of graph theoretical descriptors so that the computation of a feature vector can be simulated with a set of linear constraints in an MILP. So far, the framework has been applied to the design of new methods of inferring several restricted classes of chemical graphs such as the graphs with rank at most 2 and the ρ-lean cyclic graphs [26,28].

Herein, we examine some technical issues in the previous method before we observe some new features of our method in this paper.

In the feature vector of the previous models [26,28], the structure of subgraphs used as descriptors is only a pair of adjacent vertices, called adjacency-configuration or edge-configuration, which is significantly limited from a variety of subgraphs used in a more sophisticated construction of a feature vector such as the fingerprint. However, including the occurrence of a certain subgraph with only a few vertices as part of a feature vector may require realizing a mechanism of the subgraph isomorphism in an MILP that simulates the computation of such an occurrence and can easily make the resulting MILP very complicated and hard to solve. Furthermore, the feature vector can be defined only for cyclic graphs and we need to eliminate any acyclic graphs from the original data set before we construct a prediction function. This may reduce a data set to an unnecessarily small size or reduce the chances of capturing important information on QSAR over all types of graphs.

A branch-parameter ρ was originally introduced as a new measure to the “agglomeration degree” of trees [24] and then used to define restricted classes of acyclic and cyclic graphs [24,27]. In fact, such a restriction on the structure of target chemical graphs was rather necessary to reduce the size of an MILP formulation that simulates a selection process of a target chemical graph from a supergraph (called a scheme graph), where the number of variables and constraints required to infer a chemical graph with $n^{*}$ non-hydrogen atoms is $O(n^{*})$ when some other parameters such as ρ are regarded as constants.

Although nearly 97% of cyclic chemical compounds with up to 100 non-hydrogen atoms in PubChem are 2-lean [24], the way of specifying the topological structure of a target chemical graph in the previous method [26,28] was based on the core and the non-core of a chemical graph, and we could not include a fixed substructure in the non-core of a target chemical graph.

Compared with the previous models, the two-layered model proposed in this paper is rather simple, where a chemical graph is regarded as a combination of the interior and the exterior. The new model can deal with chemical compounds with any graph structure and include a prescribed structure in both of the acyclic and cyclic parts of a target chemical graph as long as the requirement on target chemical graphs is described under the set of specification rules introduced in this paper. This considerably improves the availability of the framework in a practical application.

The feature vector of our two-layered model can be defined for arbitrary graphs. In the new feature vector, the exterior of a chemical graph is encoded into fringe-configurations, i.e., the occurrence of each chemical rooted tree with height at most ρ, where we may regard that the set of such a chemical rooted trees plays a similar role of some types of functional groups. In our method, we include as part of the descriptors of a feature vector the occurrence of each of such chemical rooted trees and the descriptors of our feature vector on the exterior of a chemical graph may have an analogous effect with the fingerprint.

Our specification of target chemical graphs can specify a candidate set F of chemical rooted trees that are allowed to be used as chemical rooted trees in the exterior of a target chemical graph. This allows us to control the chemical property of target chemical graphs in a more meaningful way since chemical properties of some rooted trees in F are known as functional groups and some kinds of rooted trees can be prohibited in a target chemical graph, if necessary, just by excluding from the candidate set F. Although the number $\left|F(D_{\pi })\right|$ of different kinds of such chemical trees in a data set $D_{\pi }$ from PubChem is approximately up to 300 for ρ=2 in many cases and the number of input nodes in an ANN N becomes over $\left|F(D_{\pi })\right|$, we derived an MILP formulation for inferring a chemical graph with with $n^{*}$ non-hydrogen atoms and a candidate set F of chemical rooted trees by using $O(n^{*}+|F|)$ variables and $O(n^{*}|F|)$ constraints when the number of interior-vertices is constant, where |F| can be quite small compared with $\left|F(D_{\pi })\right|$.

We have implemented the proposed method for inferring chemical compounds with a prescribed topological substructure setting ρ=2. The results of computational experiments using some chemical properties such as octanol/water partition coefficient, boiling point, melting point, flash point, lipophylicity, and solubility suggest that the proposed system can infer chemical graphs with 50 non-hydrogen atoms.

For a larger branch-parameter, say ρ=3,4, we obtain a more variety of chemical rooted trees which provides new descriptors in a feature vector and new candidates for fringe-trees in the exterior in a target chemical graph, whereas the number of different chemical rooted trees in $F(D_{\pi })$ may increase rapidly.

It is left as a future work to use other learning methods such as decision tree, random forest, graph convolution, and an ensemble method in Stages 3 and 4 in the framework.

## Figures and Tables

**Table 1:** Example 1 of an interior-specification $\sigma_{\mathrm{int}}$.

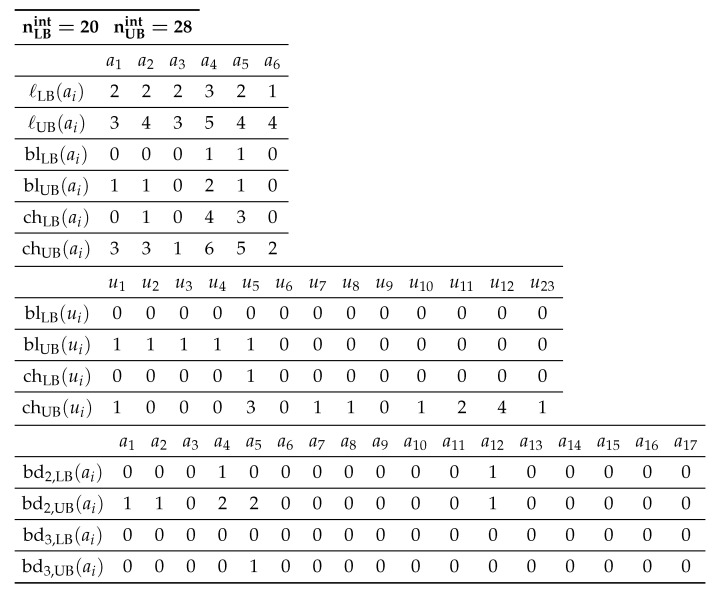

**Table 2:** Example 2 of a chemical-specification $\sigma_{\mathrm{ce}}$.

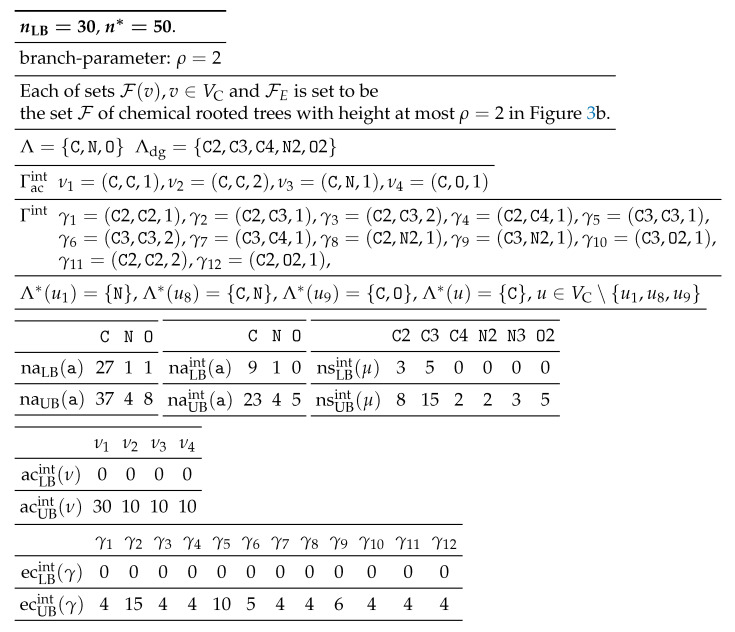

**Table 3:** Data sets for stage 1 in phase 1.

π	Λ	$|D_{\pi }|$	$|\Gamma^{\mathrm{int}}\left(D_{\pi }\right)|$	$\left|F(D_{\pi })\right|$	$\left[\underline{n},\bar{n}\right]$	$\left[\underline{a},\bar{a}\right]$
K_OW_	C,O,N	644	24	109	[4, 58]	[−7.53, 13.45]
K_OW_	C,O,N,S,Cl	837	31	142	[4, 73]	[−7.53, 13.45]
B_P_	C,O,N	358	21	91	[4, 30]	[−11.70, 470.0]
B_P_	C,O,N,S,Cl	425	23	114	[4, 30]	[−11.70, 470.0]
M_P_	C,O,N	448	22	94	[4, 122]	[−185.3, 300.0]
M_P_	C,O,N,S,Cl	548	26	118	[4, 122]	[−185.3, 300.0]
F_P_	C,O,N	348	20	85	[4, 66]	[−82.99, 300.0]
F_P_	C,O,N,S,Cl	399	24	107	[4, 66]	[−82.99, 300.0]
L_P_	C,O,N	592	27	71	[6, 60]	[−3.62, 6.84]
L_P_	C,O,N,S,Cl	779	32	78	[6, 74]	[−3.62, 6.84]
S_L_	C,O,N	640	25	111	[4, 55]	[−9.33, 1.11]
S_L_	C,O,N,S,Cl	847	31	144	[4, 55]	[−11.60, 1.11]

**Table 4:** Results of Stages 2 and 3 in Phase 1.

π	Λ	L-Time	t-$R_{\mathrm{cv}}^{2}$ (Best)	t-$R_{\max}^{2}$	Arch.
K_OW_	C,O,N	0.7	0.959	0.983	(156,10,10,1)
K_OW_	C,O,N,S,Cl	0.7	0.947	0.968	(199,20,10,1)
B_P_	C,O,N	3.5	0.858	0.923	(135,30,20,1)
B_P_	C,O,N,S,Cl	3.3	0.821	0.899	(163,10,1)
M_P_	C,O,N	3.8	0.784	0.893	(139,40,1)
M_P_	C,O,N,S,Cl	4.1	0.796	0.880	(170,10,10,1)
F_P_	C,O,N	1.1	0.750	0.874	(128,40,1)
F_P_	C,O,N,S,Cl	1.8	0.707	0.853	(157,10,10,1)
L_P_	C,O,N	0.5	0.868	0.908	(121,30,1)
L_P_	C,O,N,S,Cl	0.7	0.861	0.892	(137,20,10,1)
S_L_	C,O,N	0.7	0.870	0.913	(159,30,1)
S_L_	C,O,N,S,Cl	0.9	0.870	0.903	(201,30,20,1)

**Table 5:** Results of prediction functions by $f_{\mathrm{ec}}$ and $f_{\mathrm{fc}}$ in data set ${\tilde{D}}_{\pi }$ of cyclic graphs and $f_{\mathrm{fc}}$ in data set $D_{\pi }$ of all graphs.

	$f=f_{\mathrm{ec}}$, $D={\tilde{D}}_{\pi }$			$f=f_{\mathrm{fc}}$, $D={\tilde{D}}_{\pi }$		$f=f_{\mathrm{fc}}$, $D=D_{\pi }$		
π	$|{\tilde{D}}_{\pi }|$	t-$R_{\mathrm{cv}}^{2}$ (ave.)	t-$R_{\mathrm{cv}}^{2}$ (Best)	t-$R_{\mathrm{cv}}^{2}$ (ave.)	t-$R_{\mathrm{cv}}^{2}$ (Best)	$|D_{\pi }|$	t-$R_{\mathrm{cv}}^{2}$ (ave.)	t-$R_{\mathrm{cv}}^{2}$ (Best)
K_OW_	580	0.952	0.959	0.950	0.954	837	0.944	0.947
B_P_	224	0.688	0.718	0.680	0.693	425	0.809	0.821
M_P_	348	0.668	0.694	0.712	0.736	548	0.776	0.796
F_P_	218	0.435	0.476	0.574	0.623	399	0.688	0.707
L_P_	776	0.832	0.842	0.853	0.861	779	0.854	0.861
S_L_	638	0.851	0.863	0.853	0.861	847	0.860	0.870

**Table 6:** Features of test instances.

Instance	Λ	$|\Gamma^{\mathrm{int}}|$	$|F^{*}|$	$\left[n_{\mathrm{LB}}^{\mathrm{int}},n_{\mathrm{UB}}^{\mathrm{int}}\right]$	$\left[n_{\mathrm{LB}},n^{*}\right]$
$I_{a}$	C,O,N	10	11	[30,50]	[20,28]
$I_{b,1}$	C,O,N	28	40	[38,38]	[6,6]
$I_{b,2}$	C,O,N	28	35	[50,50]	[30,30]
$I_{b,3}$	C,O,N	28	30	[50,50]	[30,30]
$I_{b,4}$	C,O,N	28	25	[50,50]	[30,30]
$I_{c}$	C,O,N	8	12	[46,46]	[24,24]
$I_{d}$	C,O,N	7	8	[40,45]	[18,18]

**Table 7:** Results of Stage 4 for K_OW_.

Instance	$\left[\underline{a},\bar{a}\right]$	$\left[\underline{y},\;\bar{y}\right]$	$y^{*}$	#v	#c	IP-Time	*n*	$n^{\mathrm{int}}$
$I_{a}$	[−7.53, 13.45]	[−7.0, 13.4]	3.2	7663	9162	3.9	35	24
$I_{b,1}$	[−7.53, 13.45]	[−7.5, 13.4]	3.0	9894	6626	17.5	38	7
$I_{b,2}$	[−7.53, 13.45]	[−7.5, 13.4]	3.0	11,514	8934	14.0	50	30
$I_{b,3}$	[−7.53, 13.45]	[−7.5, 13.4]	3.0	11,318	8926	24.6	50	30
$I_{b,4}$	[−7.53, 13.45]	[−7.5, 13.4]	3.0	11,122	8918	22.0	50	30
$I_{c}$	[−7.53, 13.45]	[−7.5, 13.4]	3.0	7867	8630	2.1	49	32
$I_{d}$	[−7.53, 13.45]	[−7.5, 13.4]	3.0	5395	6899	5.2	45	23

**Table 8:** Results of Stage 4 for B_P_.

Instance	$\left[\underline{a},\bar{a}\right]$	$\left[\underline{y},\;\bar{y}\right]$	$y^{*}$	#v	#c	IP-Time	*n*	$n^{\mathrm{int}}$
$I_{a}$	[−11.70, 470.0]	[352, 470]	411	7583	8982	2.7	42	25
$I_{b,1}$	[−11.70, 470.0]	[−11, 470]	229	9816	6449	2.7	38	7
$I_{b,2}$	[−11.70, 470.0]	[−11, 470]	229	11,436	8757	9.1	50	30
$I_{b,3}$	[−11.70, 470.0]	[−11, 470]	229	11,240	8749	11.0	50	30
$I_{b,4}$	[−11.70, 470.0]	[−11, 470]	229	11,044	8741	24.0	50	30
$I_{c}$	[−11.70, 470.0]	[170, 470]	320	7575	8450	25.9	49	33
$I_{d}$	[−11.70, 470.0]	[151, 470]	310	5315	6719	4.4	43	23

**Table 9:** Results of Stage 4 for M_P_.

Instance	$\left[\underline{a},\bar{a}\right]$	$\left[\underline{y},\;\bar{y}\right]$	$y^{*}$	#v	#c	IP-Time	*n*	$n^{\mathrm{int}}$
$I_{a}$	[−185.3, 300.0]	[55, 300]	177.5	7602	9023	16.1	41	24
$I_{b,1}$	[−185.3, 300.0]	[−180, 300]	60	9833	6487	2.3	38	9
$I_{b,2}$	[−185.3, 300.0]	[−185, 300]	57.4	11,453	8795	44.7	50	30
$I_{b,3}$	[−185.3, 300.0]	[−185, 300]	57.4	11,257	8787	10.5	50	30
$I_{b,4}$	[−185.3, 300.0]	[−185, 300]	57.4	11,061	8779	93.9	50	30
$I_{c}$	[−185.3, 300.0]	[253, 300]	260.0	7580	6172	24.0	41	33
$I_{d}$	[−185.3, 300.0]	[−75, 299]	58	5110	4050	104.6	45	23

**Table 10:** Results of Stage 4 for F_P_.

Instance	$\left[\underline{a},\bar{a}\right]$	$\left[\underline{y},\;\bar{y}\right]$	$y^{*}$	#v	#c	IP-Time	*n*	$n^{\mathrm{int}}$
$I_{a}$	[−82.99, 300.0]	[98, 300]	199	7459	8696	1.6	35	22
$I_{b,1}$	[−82.99, 300.0]	[−82, 300]	109	9694	6166	1.4	38	8
$I_{b,2}$	[−82.99, 300.0]	[−82, 300]	109	11,314	8474	8.7	50	30
$I_{b,3}$	[−82.99, 300.0]	[−82, 300]	109	11,118	8466	25.8	50	30
$I_{b,4}$	[−82.99, 300.0]	[−82, 300]	109	10,922	8458	8.5	50	30
$I_{c}$	[−82.99, 300.0]	[250, 300]	275	7667	8170	60.9	47	34
$I_{d}$	[−82.99, 300.0]	[54, 300]	177	5193	6436	2.0	45	23

**Table 11:** Results of Stage 4 for L_P_.

Instance	$\left[\underline{a},\bar{a}\right]$	$\left[\underline{y},\;\bar{y}\right]$	$y^{*}$	#v	#c	IP-Time	*n*	$n^{\mathrm{int}}$
$I_{a}$	[−3.6, 6.84]	[−3.6, 6.8]	1.6	7597	9008	1.9	39	23
$I_{b,1}$	[−3.6, 6.84]	[−3.6, 6.8]	1.6	9836	6481	2.9	38	8
$I_{b,2}$	[−3.6, 6.84]	[−3.6, 6.8]	1.6	11,456	8789	21.1	50	30
$I_{b,3}$	[−3.6, 6.84]	[−3.6, 6.8]	1.6	11,260	8781	20.4	50	30
$I_{b,4}$	[−3.6, 6.84]	[−3.6, 6.8]	1.6	11,064	8773	24.2	50	30
$I_{c}$	[−3.6, 6.84]	[−3.6, 6.8]	1.6	7801	8476	1.1	47	32
$I_{d}$	[−3.6, 6.84]	[−3.6, 6.8]	1.6	5335	6754	4.3	45	23

**Table 12:** Results of Stage 4 for S_L_.

Instance	$\left[\underline{a},\bar{a}\right]$	$\left[\underline{y},\;\bar{y}\right]$	$y^{*}$	#v	#c	IP-Time	*n*	$n^{\mathrm{int}}$
$I_{a}$	[−9.33, 1.11]	[−9.3, −2.0]	−5.6	7674	9186	2.4	41	23
$I_{b,1}$	[−9.33, 1.11]	[−9.3, −2.0]	−5.6	9906	6650	22.3	38	12
$I_{b,2}$	[−9.33, 1.11]	[−9.3, −2.0]	−5.6	11,526	8958	15.2	50	30
$I_{b,3}$	[−9.33, 1.11]	[−9.3, −2.0]	−5.6	11,330	8950	16.2	50	30
$I_{b,4}$	[−9.33, 1.11]	[−9.3, −2.0]	−5.6	11,134	8942	122.7	50	30
$I_{c}$	[−9.33, 1.11]	[−9.3, −2.0]	−5.6	7874	8648	1.2	54	33
$I_{d}$	[−9.33, 1.11]	[−9.3, −3.0]	−6.1	5402	6917	8.1	43	23

**Table 13:** Results of Stage 5 for K_OW_, L_P_, and B_P_.

		Kow			Lp			Bp	
Instance	DP-Time	G-LB	#G	DP-Time	G-LB	#G	DP-time	G-LB	#G
$I_{a}$	0.031	16	16	0.164	128	100	0.164	$1.4\times 10^{5}$	100
$I_{b}^{1}$	0.149	$2.8\times 10^{5}$	100	0.148	$2.0\times 10^{10}$	100	0.162	$4.4\times 10^{5}$	100
$I_{b}^{2}$	44.1	$3.9\times 10^{10}$	100	118	900	100	171	6	6
$I_{b}^{3}$	27.2	20	20	80.2	6	6	28.6	7	7
$I_{b}^{4}$	0.166	6000	100	73	12	12	142	5	5
$I_{c}$	0.166	6000	100	0.168	288	100	0.168	$4.0\times 10^{5}$	100
$I_{d}$	22.3	$8.3\times 10^{10}$	100	1.44	$3.2\times 10^{8}$	100	1.7	$9.7\times 10^{9}$	100

**Table 14:** Results of Stage 5 for F_P_, M_P_, and S_L_.

		F_P_			M_P_			S_L_	
Instance	DP-Time	G-LB	#G	DP-Time	G-LB	#G	DP-Time	G-LB	#G
$I_{a}$	0.057	32	32	0.165	256	100	0.165	1024	100
$I_{b}^{1}$	0.164	$3.1\times 10^{6}$	100	0.166	$1.4\times 10^{6}$	100	0.163	$4.5\times 10^{5}$	100
$I_{b}^{2}$	28.8	720	100	8.26	$2.4\times 10^{10}$	100	1.07	$5.6\times 10^{9}$	100
$I_{b}^{3}$	72.2	27	27	51.9	1	1	46.5	1680	100
$I_{b}^{4}$	40.3	20	20	125	$6.1\times 10^{7}$	100	7.01	$1.1\times 10^{8}$	100
$I_{c}$	0.169	$1.1\times 10^{5}$	100	0.173	6048	100	0.168	120	100
$I_{d}$	0.057	32	32	0.17	$4.2\times 10^{8}$	100	0.165	1024	100

## Data Availability

Source code of the implementation of our algorithm is freely available from https://github.com/ku-dml/mol-infer.

## Supplementary Materials

The following are available online at https://www.mdpi.com/1422-0067/22/6/2847/s1.

## Abbreviations

ANN	artificial neural network
MILP	mixed integer linear programming
