# Supplementary material for: An Inverse QSAR Method Based on a Two-Layered Model and Integer Programming
Source: Int J Mol Sci. 2021 Mar 11;22(6):2847. doi: 10.3390/ijms22062847 (PMC8002091; doi:10.3390/ijms22062847)
Supplement: Supplementary file 1 [file ijms-22-02847-s001.pdf]

Supplementary Materials

An Inverse QSAR Method Based on a Two-layered Model and Integer Programming

Yu Shi[1], Jianshen Zhu[1], Naveed Ahmed Azam[1], Kazuya Haraguchi[1], Liang Zhao[2], Hiroshi Nagamochi[1], Tatsuya Akutsu[3]

[1] Department of Applied Mathematics and Physics, Kyoto University, Kyoto 606-8501, Japan;
[2] Graduate School of Advanced Integrated Studies in Human Survivability (Shishu-Kan), Kyoto University, Kyoto 606-8306, Japan;
[3] Bioinformatics Center, Institute for Chemical Research, Kyoto University, Uji 611-0011, Japan

1 An MILP Formulation for Inferring a Target Chemical Graph in Stage 4

1.1 Constructing Target Chemical Graphs

This section describes how to construct a target chemical graph in Stages 4 and 5.

1.1.1 Formulating an MILP for a prediction function in Stage 4

In Stage 3, we construct a prediction function $\eta_{\mathcal{N}} : \mathbb{R}^K \to \mathbb{R}$. It is known that the computation process of $\eta_{\mathcal{N}}(x)$ from a vector $x^* \in \mathbb{R}^K$ can be formulated as an MILP with the following property.

Theorem 1. ([1, 2]) *Let $\mathcal{N}$ be an ANN with a piecewise-linear activation function for an input vector $x \in \mathbb{R}^K$, n_A denote the number of nodes in the architecture and n_B denote the total number of breakpoints over all activation functions. Then there is an MILP $\mathcal{M}(x, y; \mathcal{C}_1)$ that consists of variable vectors $x \in \mathbb{R}^K$, $y \in \mathbb{R}$, and an auxiliary variable vector $z \in \mathbb{R}^p$ for some integer $p = O(n_A + n_B)$ and a set $\mathcal{C}_1$ of $O(n_A + n_B)$ constraints on these variables such that: $\eta_{\mathcal{N}}(x^*) = y^*$ if and only if there is a vector (x^*, y^*) feasible to $\mathcal{M}(x, y; \mathcal{C}_1)$.*

Solving this MILP delivers a vector $x^* \in \mathbb{R}^K$ such that $\eta_{\mathcal{N}}(x^*) = y^*$ for a target value $\boldsymbol{y}^*$. However, the resulting vector x^* may not admit a chemical graph G^* such that $f(G^*) = x^*$. To ensure that such chemical graph always exists in Stage 4, we further introduce some more constraints for a set of new variables in the next section.

1.1.2 Formulating an MILP for a feature vector and a target specification in Stage 4

In this section, we show an outline of formulation of an MILP that represents the computation process of a feature function $f(G)$ from a chemical graph G and a construction of a target chemical graph $G \in \mathcal{G}(G_{\mathrm{C}}, \sigma_{\mathrm{int}}, \sigma_{\mathrm{ce}})$. Recall that the number of vertices in a target chemical graph is bounded by an upper bound n^* in a specification $(G_{\mathrm{C}}, \sigma_{\mathrm{int}}, \sigma_{\mathrm{ce}})$. However, if we introduce a set of $(n^*)^2$ variables for all pairs of n^* vertices to present all possible graphs for a target chemical graph, then the resulting MILP formulation is hard to solve for $n^* > 20$ due to a larger number of variables and constraints. To overcome this, a sparse representation of chemical graphs has been proposed in the previous applications of the framework for acyclic graphs [3] and ρ-lean graphs [4]. We also define a similar sparse representation to formulate an MILP for our two-layered model.

Scheme Graphs We first regard a given seed graph $G_{\rm C}$ as a digraph and then add some more vertices and edges to construct a digraph, called a *scheme graph* $\mathrm{SG} = (\mathcal{V}, \mathcal{E})$ so that any $(\sigma_{\rm int}, \sigma_{\rm ce})$-extension H of $G_{\rm C}$ can be chosen as a subgraph of SG.

For a given target specification $(G_{\rm C}, \sigma_{\rm int}, \sigma_{\rm ce})$, define integers that determine the size of a scheme graph SG as follows. $m_{\rm C} := |E_{\rm C}|$, $t_{\rm C} := |V_{\rm C}|$, $t_{\rm T} := \mathrm{n}^{\rm int}_{\rm UB} - |V_{\rm C}|$, and $t_{\rm F} := n^* - \mathrm{n}^{\rm int}_{\rm LB}$.

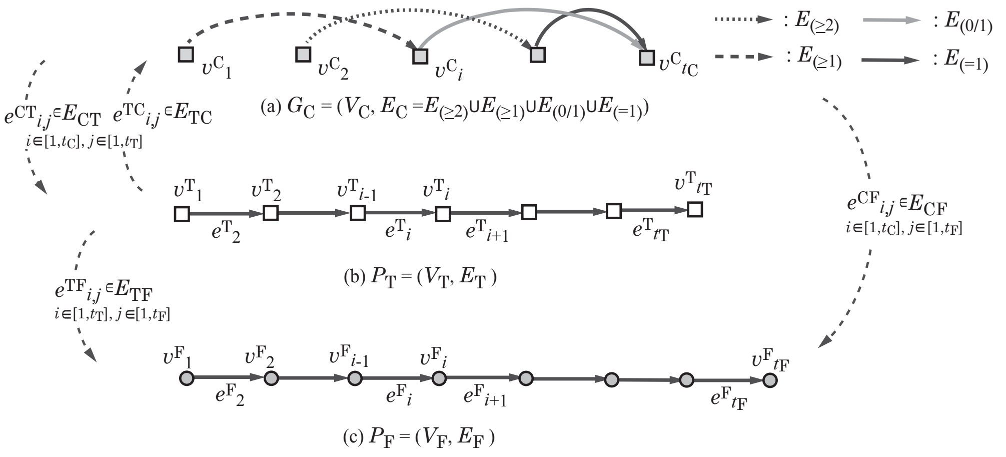

Figure 1: An illustration of a scheme graph SG: (a) A seed graph $G_{\rm C}$; (b) A path $P_{\rm T}$ of length $t_{\rm T}-1$; (c) A path $P_{\rm F}$ of length $t_{\rm F}-1$.

Formally the scheme graph $\mathrm{SG} = (\mathcal{V}, \mathcal{E})$ is defined with a vertex set $\mathcal{V} = V_{\rm C} \cup V_{\rm T} \cup V_{\rm F}$ and an edge set $\mathcal{E} = E_{\rm C} \cup E_{\rm T} \cup E_{\rm F} \cup E_{\rm CT} \cup E_{\rm TC} \cup E_{\rm CF} \cup E_{\rm TF}$ that consist of the following sets. See Figure 1 for an illustration of these sets.

Construction of a $\sigma_{\rm int}$-extension H^* of $G_{\rm C}$: Denote the vertex set $V_{\rm C}$ and the edge set $E_{\rm C}$ in the seed graph $G_{\rm C}$ by $V_{\rm C} = \{v^{\rm C}{}_i \mid i \in [1, t_{\rm C}]\}$ and $E_{\rm C} = \{a_i \mid i \in [1, m_{\rm C}]\}$, respectively, where $V_{\rm C}$ is always included in H^*. For including additional interior-vertices in H^*, introduce a path $P_{\rm T} = (V_{\rm T} = \{v^{\rm T}{}_1, v^{\rm T}{}_2, \ldots, v^{\rm T}{}_{t_{\rm T}}\}, E_{\rm T} = \{e^{\rm T}{}_2, e^{\rm T}{}_3, \ldots, e^{\rm T}{}_{t_{\rm T}}\})$ of length $t_{\rm T}-1$ and a set $E_{\rm CT}$ (resp., $E_{\rm TC}$) of directed edges $e^{\rm CT}{}_{i,j} = (v^{\rm C}{}_i, v^{\rm T}{}_j)$ (resp., $e^{\rm TC}{}_{i,j} = (v^{\rm T}{}_j, v^{\rm C}{}_i)$) $i \in [1, t_{\rm C}]$, $j \in [1, t_{\rm T}]$. In H^*, an edge $a_k = (v^{\rm C}{}_i, v^{\rm C}{}_{i'}) \in E_{(\geq 2)} \cup E_{(\geq 1)}$ is allowed to be replaced with a pure path P_k from vertex $v^{\rm C}{}_i$ to vertex $v^{\rm C}{}_{i'}$ that visits a set of consecutive vertices $v^{\rm T}{}_j, v^{\rm T}{}_{j+1}, \ldots, v^{\rm T}{}_{j+p} \in V_{\rm T}$ and edge $e^{\rm TC}{}_{i,j} = (v^{\rm C}{}_i, v^{\rm T}{}_j) \in E_{\rm CT}$, then edges $e^{\rm T}{}_{j+1}, e^{\rm T}{}_{j+2}, \ldots, e^{\rm T}{}_{j+p} \in E_{\rm T}$ and finally edge $e^{\rm TC}{}_{i',j+p} = (v^{\rm T}{}_{j+p}, v^{\rm C}{}_{i'}) \in E_{\rm TC}$. The vertices in $V_{\rm T}$ selected in the path will be vertices in H^*.

Appending leaf paths with additional interior-edges in a $(\sigma_{\rm int}, \sigma_{\rm ce})$-extension H of $G_{\rm C}$: Introduce a path $P_{\rm F} = (V_{\rm F} = \{v^{\rm F}{}_1, v^{\rm F}{}_2, \ldots, v^{\rm F}{}_{t_{\rm F}}\}, E_{\rm F} = \{e^{\rm F}{}_2, e^{\rm F}{}_3, \ldots, e^{\rm F}{}_{t_{\rm F}}\})$ of length $t_{\rm F}-1$, a set $E_{\rm CF}$ of directed edges $e^{\rm CF}{}_{i,j} = (v^{\rm C}{}_i, v^{\rm F}{}_j)$, $i \in [1, t_{\rm C}]$, $j \in [1, t_{\rm F}]$, and a set $E_{\rm TF}$ of directed edges $e^{\rm TF}{}_{i,j} = (v^{\rm T}{}_i, v^{\rm F}{}_j)$, $i \in [1, t_{\rm T}]$, $j \in [1, t_{\rm F}]$. In H, a leaf path Q with interior-edges that starts from a vertex $v^{\rm C}{}_i \in V_{\rm C}$ (resp., $v^{\rm T}{}_i \in V_{\rm T}$) visits a set of consecutive vertices $v^{\rm F}{}_j, v^{\rm F}{}_{j+1}, \ldots, v^{\rm F}{}_{j+p} \in V_{\rm F}$ and edge $e^{\rm CF}{}_{i,j} = (v^{\rm C}{}_i, v^{\rm F}{}_j) \in E_{\rm CF}$ (resp., $e^{\rm TF}{}_{i,j} = (v^{\rm T}{}_i, v^{\rm F}{}_j) \in E_{\rm TF}$) and edges $e^{\rm F}{}_{j+1}, e^{\rm F}{}_{j+2}, \ldots, e^{\rm F}{}_{j+p} \in E_{\rm F}$. In H, the edges and the vertices selected in the path Q are regarded as interior-edges and interior-vertices, respectively.

Construction of ρ-fringe-trees in a $(\sigma_{\rm int}, \sigma_{\rm ce})$-extension G of $G_{\rm C}$: In H, the root of a ρ-fringe-tree can be any vertex in $V_{\rm C} \cup V_{\rm T} \cup V_{\rm F}$. For each vertex $v = v^{\rm C}{}_i$ (resp., $v = v^{\rm T}{}_i$ or $v^{\rm F}{}_i$), we choose a chemical rooted tree T from the specified set $\mathcal{F}(v)$ (resp., $\mathcal{F}_E$).

Recall that the dimension K of a feature vector $x = f(G)$ used in constructing a prediction function $\eta_{\mathcal{N}}$ over a set of chemical graphs G is $K = 17+|\Lambda^{\rm int}(D_\pi)|+|\Lambda^{\rm ex}(D_\pi)|+|\Gamma^{\rm int}(D_\pi)|+|\mathcal{F}(D_\pi)|$. For a target specification $(G_{\rm C}, \sigma_{\rm int}, \sigma_{\rm ce})$, let $\mathcal{F}^*$ denote the set of chemical rooted trees ψ in the sets $\mathcal{F}(v), v \in V_{\rm C}$ and $\mathcal{F}_E$ and $K^* := 17+|\Lambda^{\rm int}(D_\pi)|+|\Lambda^{\rm ex}(D_\pi)|+|\Gamma^{\rm int}(D_\pi)|+|\mathcal{F}^*|$. Based on the scheme graph SG, we obtain the following MILP formulation $\mathcal{M}(x, g; \mathcal{C}_2)$.

Theorem 2. *Let $(G_{\rm C}, \sigma_{\rm int}, \sigma_{\rm ce})$ be a target specification and $\varphi^* = |\Lambda^{\rm int}(D_\pi)|+|\Lambda^{\rm ex}(D_\pi)|+|\Gamma^{\rm int}(D_\pi)|+|\mathcal{F}^*|$ for sets of chemical elements, edge-configurations and fringe-configurations in $\sigma_{\rm ce}$. Then there is an MILP $\mathcal{M}(x, g; \mathcal{C}_2)$ that consists of variable vectors $x \in \mathbb{R}^{K^*}$ and $g \in \mathbb{R}^q$ for an integer $q = O(\mathrm{n}^{\rm int}_{\rm UB}(|E_{\rm C}| + n^*) + (|E_{\rm C}| + |\mathcal{V}|)\varphi^*)$ and a set $\mathcal{C}_2$ of $O([\mathrm{n}^{\rm int}_{\rm UB}(|E_{\rm C}| + n^*) + |\mathcal{V}|]\varphi^*)$ constraints on x and g such that: (x^*, g^*) is feasible to $\mathcal{M}(x, g; \mathcal{C}_2)$ if and only if g^* forms a chemical graph $G \in \mathcal{G}(G_{\rm C}, \sigma_{\rm int}, \sigma_{\rm ce})$ such that $f(G) = x^*$.*

Note that our MILP requires only $O(n^*)$ variables and constraints when the branch-parameter ρ, integers $|E_{\rm C}|$, $\mathrm{n}^{\rm int}_{\rm UB}$ and φ^* are constant. We explain the basic idea of our MILP that satisfies Theorem 2. The MILP mainly consists of the following three types of constraints.

C1. Constraints for selecting an underlying graph H of a chemical graph $G \in \mathcal{G}(G_{\rm C}, \sigma_{\rm int}, \sigma_{\rm ce})$ as a subgraph of the scheme graph SG;

C2. Constraints for assigning chemical elements to interior-vertices and multiplicity to interior-edges to determine a chemical graph $G = (H, \alpha, \beta)$; and

C3. Constraints for computing descriptors in the feature vector $f(G)$ of the selected chemical graph G.

In the constraints of C1, more formally we prepare the following.

Variables:

- a binary variable $v^{\rm X}(i) \in \{0, 1\}$ for each vertex $v^{\rm X}{}_i \in V_{\rm X}$, $\mathrm{X} \in \{\mathrm{C}, \mathrm{T}, \mathrm{F}\}$ so that $v^{\rm X}(i) = 1 \Leftrightarrow$ vertex $v^{\rm X}{}_i$ is used in a graph H selected from SG;
- a binary variable $e^{\rm X}(i) \in \{0, 1\}$ (resp., $e^{\rm C}(i) \in \{0, 1\}$) for each edge $e^{\rm X}{}_i \in E_{\rm T} \cup E_{\rm F}$ (resp., $e^{\rm C}{}_i = a_i \in E_{(\geq 2)} \cup E_{(\geq 1)} \cup E_{(0/1)}$) so that $e^{\rm X}(i) = 1 \Leftrightarrow$ edge $e^{\rm X}{}_i$ is used in a graph H selected from SG. To save the number of variables in our MILP formulation, we do not prepare a binary variable $e^{\rm X}(i, j) \in \{0, 1\}$ for any edge $e^{\rm X}{}_{i,j} \in E_{\rm CT} \cup E_{\rm TC} \cup E_{\rm CF} \cup E_{\rm TC}$, where we represent a choice of edges in these sets by a set of $O(n^*|E_{\rm C}|)$ variables (see Supplementary Materials for the details);
- binary variables $\delta^{\rm C}_{\rm fr}(i, \psi) \in \{0, 1\}, i \in [1, t_{\rm C}], \psi \in \mathcal{F}(v), v = v^{\rm C}{}_i \in V_{\rm C}$ and $\delta^{\rm T}_{\rm fr}(i, \psi) \in \{0, 1\}, i \in [1, t_{\rm T}], \delta^{\rm F}_{\rm fr}(i, \psi) \in \{0, 1\}, i \in [1, t_{\rm F}], \psi \in \mathcal{F}_E$, where $\delta^{\rm X}_{\rm fr}(i, \psi) = 1$ ($\mathrm{X} \in \{\mathrm{C}, \mathrm{T}, \mathrm{F}\}$) if and only if the ρ-fringe-tree rooted at vertex $v^{\rm X}{}_i$ is r-isomorphic to ψ.

Constraints:

- linear constraints so that each ρ-fringe-tree rooted at a vertex $v^{\rm X}{}_i$ in a graph H from SG is selected from the given set $\mathcal{F}(v^{\rm C}{}_i)$ for X=C (or $\mathcal{F}_E$ for $\mathrm{X} \in \{\mathrm{T}, \mathrm{F}\}$);
- linear constraints such that each edge $e^{\rm C}{}_i = a_i \in E_{(=1)}$ is always used as an edge in H and each edge $e^{\rm C}{}_i = a_i \in E_{(0/1)}$ is used as an edge in H if necessary;

- linear constraints such that for each edge $a_k = (v^{\mathrm{C}}{}_i, v^{\mathrm{C}}{}_{i'}) \in E_{(\geq 2)}$, vertex $v^{\mathrm{C}}{}_i \in V_{\mathrm{C}}$ is connected to vertex $v^{\mathrm{C}}{}_{i'} \in V_{\mathrm{C}}$ in H by a pure path P_k that passes through some vertices in V_{T} and edges $e^{\mathrm{CT}}{}_{i,j}, e^{\mathrm{T}}{}_{j+1}, e^{\mathrm{T}}{}_{j+2}, \ldots, e^{\mathrm{T}}{}_{j+p}, e^{\mathrm{TC}}{}_{i',j+p}$ for some integers j and p;
- linear constraints such that for each edge $a_k = (v^{\mathrm{C}}{}_i, v^{\mathrm{C}}{}_{i'}) \in E_{(\geq 1)}$, either the edge a_k is used as an edge in H or vertex $v^{\mathrm{C}}{}_i \in V_{\mathrm{C}}$ is connected to vertex $v^{\mathrm{C}}{}_{i'} \in V_{\mathrm{C}}$ in H by a pure path P_k as in the case of edges in $E_{(\geq 2)}$;
- linear constraints for selecting a leaf path Q_v rooted at a vertex $v = v^{\mathrm{C}}{}_i$ (resp., $v = v^{\mathrm{T}}{}_i$) with ρ-internal edges $e^{\mathrm{CF}}{}_{i,j}$ (resp., $e^{\mathrm{TF}}{}_{i,j}$), $e^{\mathrm{F}}{}_{j+1}, e^{\mathrm{F}}{}_{j+2}, \ldots, e^{\mathrm{F}}{}_{j+p}$ for some integers j and p.

In the constraints of C2, we prepare an integer variable $\alpha^{\mathrm{X}}(i)$ for each vertex $v^{\mathrm{X}}{}_i \in \mathcal{V}$, $\mathrm{X} \in \{\mathrm{C}, \mathrm{T}, \mathrm{F}\}$ in the scheme graph that represents the chemical element $\alpha(v^{\mathrm{X}}{}_i) \in \Lambda$ if $v^{\mathrm{X}}{}_i$ is in a selected graph H (or $\alpha(v^{\mathrm{X}}{}_i) = 0$ otherwise); integer variables $\beta^{\mathrm{C}} : E_{\mathrm{C}} \to [0,3]$, $\beta^{\mathrm{T}} : E_{\mathrm{T}} \to [0,3]$ and $\beta^{\mathrm{F}} : E_{\mathrm{F}} \to [0,3]$ that represent the bond-multiplicity of edges in $E_{\mathrm{C}} \cup E_{\mathrm{T}} \cup E_{\mathrm{F}}$; and integer variables $\beta^{+}, \beta^{-} : E_{(\geq 2)} \cup E_{(\geq 1)} \to [0,3]$ and $\beta^{\mathrm{in}} : V_{\mathrm{C}} \cup V_{\mathrm{T}} \to [0,3]$ that represent the bond-multiplicity of edges in $E_{\mathrm{CT}} \cup E_{\mathrm{TC}} \cup E_{\mathrm{CF}} \cup E_{\mathrm{TF}}$. This determines a chemical graph $G = (H, \alpha, \beta)$. Also we include constraints for a selected chemical graph G to satisfy the valence condition at each interior-vertex v with the edge-configurations $\mathrm{ec}(e)$ of the edges e incident to v and the chemical specification σ_{ce}.

In the constraints of C3, we introduce a variable for each descriptor and constraints with some more variables to compute the value of each descriptor in $f(G)$ for a selected chemical graph G.

The details of the MILP can be found in Section 3.

2 A Dynamic Programming Algorithm for Generating Isomers in Stage 5

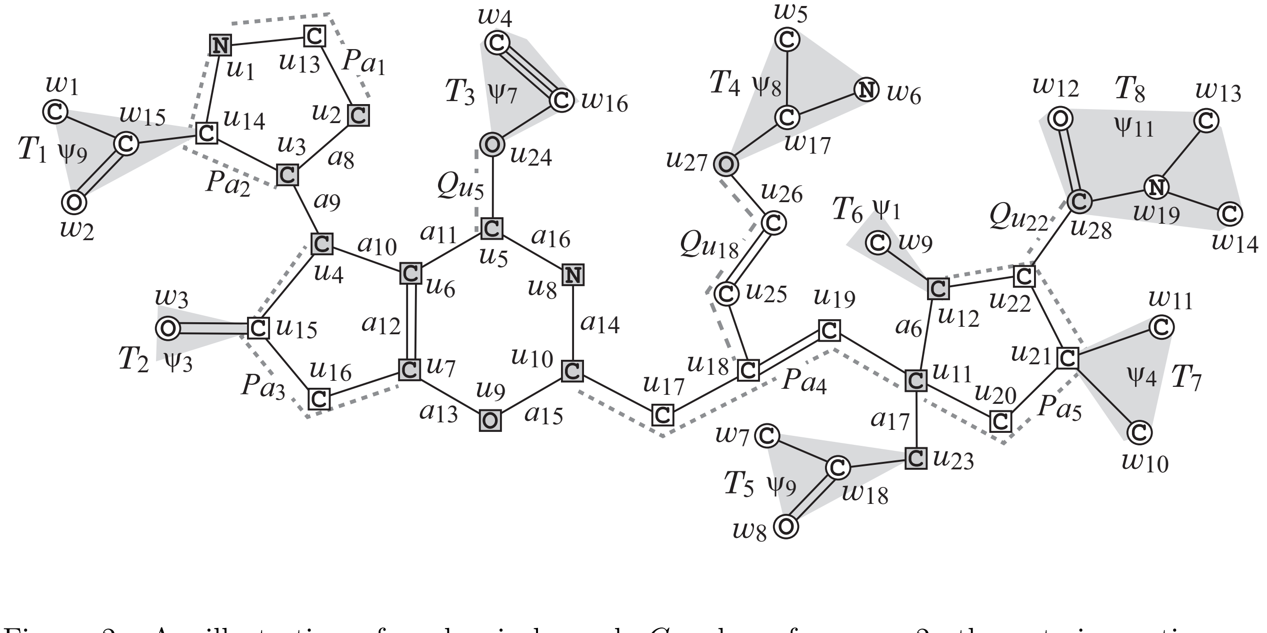

Figure 2: An illustration of a chemical graph G, where for $\rho = 2$, the exterior-vertices are $w_1, w_2, \ldots, w_{19}$ and the interior-vertices are $u_1, u_2, \ldots, u_{28}$.

This section briefly reviews the method [4] for Stage 5. Let $G^{\dagger}$ be a chemical graph that is a $(\sigma_{\mathrm{int}}, \sigma_{\mathrm{ce}})$-extension of a seed graph $G_{\mathrm{C}} = (V_{\mathrm{C}}, E_{\mathrm{C}})$, where we denote by $E_{(=0)}$ the set of the edges

in $E_{(0/1)}$ that are not used in $G^\dagger$. We define a *base-graph* $G_B = (V_B, E_B)$ to be the seed graph $(V_{\rm C}, E_{\rm C} \setminus E_{(=0)})$ after removing the edges in $E_{(=0)}$. We call a chemical graph G^* a *chemical isomer* of $G^\dagger$ if $f(G^*) = f(G^\dagger)$ and G^* is also a $(\sigma_{\rm int}, \sigma_{\rm ce})$-extension of G_B.

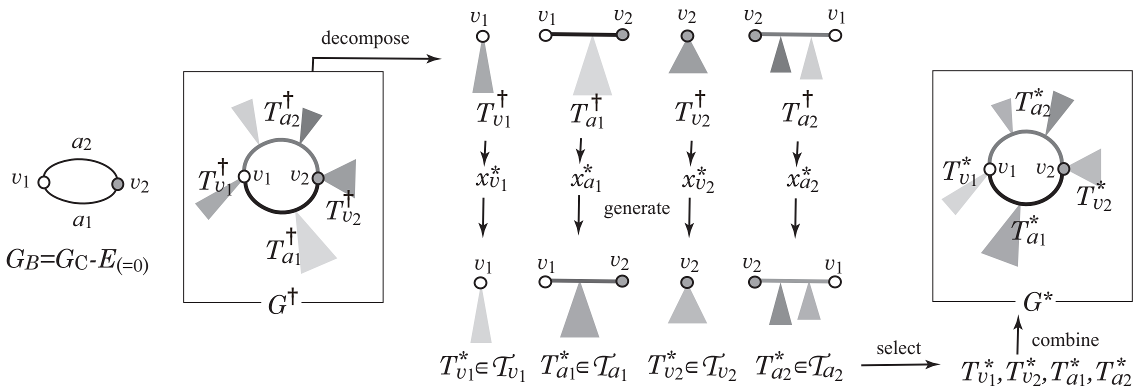

Figure 3: An illustration of generating a chemical isomer G^* of a chemical graph $G^\dagger$ with a base-graph $G_B = (V_B, E_B)$.

The method generates chemical isomers G^* of $G^\dagger$ in the following way, where Figure 3 illustrates the whole process in the case of $V_B = \{v_1, v_2\}$ and $E_B = \{a_1, a_2\}$.

1. We first decompose a given chemical graph $G^\dagger$ into a collection of chemical rooted or bi-rooted trees.

   - For each vertex $v \in V_B$, let $T^\dagger_v$ denote the chemical rooted tree rooted at v in G that is constructed with a leaf path Q_v and fringe-trees attached to Q_v. Possibly $T^\dagger_v$ consists of a single vertex v and we call such a tree *trivial*.
   - For each edge $a = uv \in E_{(\geq 2)} \cup E_{(\geq 1)}$, let $T^\dagger_a$ denote the chemical bi-rooted tree rooted at vertices u and v in G that consists of a pure u,v-path P_a, leaf paths rooted at internal vertices in P_a and fringe-trees attached to theses leaf paths. Possibly $T^\dagger_a$ consists of a single edge a and we call such a tree *trivial*.

   Figure 4 illustrates the non-trivial chemical trees $T^\dagger_{\rm t}, {\rm t} \in V^*_B \cup E^*_B$ of the $(\sigma_{\rm int}, \sigma_{\rm ce})$-extension $G^\dagger = G$ in Figure 2.

2. Let V^*_B (resp., E^*_B) denote the set of vertices $v \in V_B$ (resp., $a \in E_B$) such that $T^\dagger_v$ (resp., $T^\dagger_a$) is not trivial. For each vertex or edge ${\rm t} \in V^*_B \cup E^*_B$, compute the feature vector $x^*_{\rm t} = f(T^\dagger_{\rm t})$ and then generate a set $\mathcal{T}_{\rm t}$ of all (or a limited number of) chemical acyclic graphs $T^*_{\rm t}$ such that $f(T^*_{\rm t}) = x^*_{\rm t}$ and the structure of $T^*_{\rm t}$ satisfies the lower and upper bounds in the interior-specification $\sigma_{\rm int}$ by using the dynamic programming algorithm for chemical acyclic graphs [3].

3. For each combination of chemical trees $T^*_{\rm t} \in \mathcal{T}_{\rm t}, {\rm t} \in V^*_B \cup E^*_B$, a chemical graph G^* such that $f(G^*) = f(G^\dagger)$ is obtained from $G^\dagger$ by replacing each tree $T^\dagger_{\rm t}$ with a new tree $T^*_{\rm t}$. The number of such combinations is $\prod_{{\rm t} \in V^*_B \cup E^*_B} |\mathcal{T}_{\rm t}|$, where we ignore a possible automorphism of the resulting graphs G^*.

The above method [4] can be used to generate chemical isomers in Stage 5 in our two-layered model by making a minor modification to the definition of a feature vector $f(G)$.

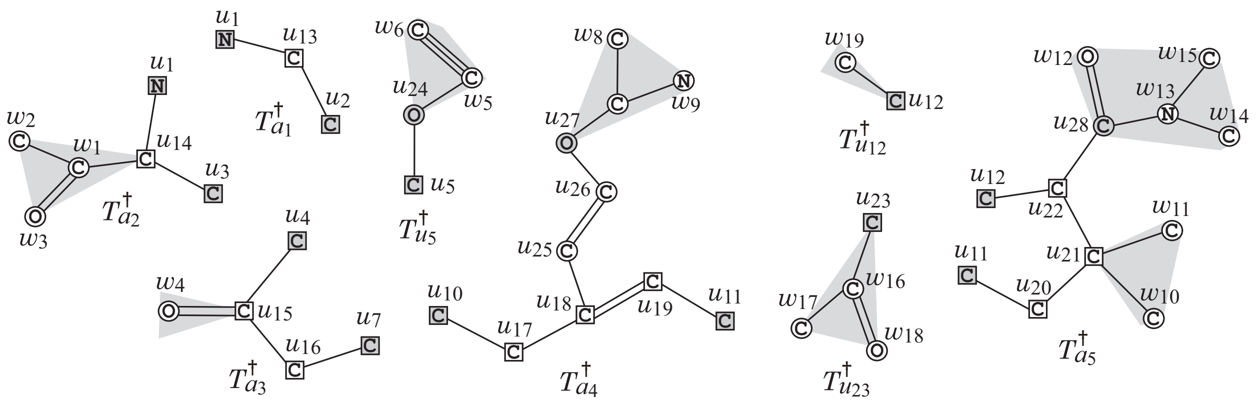

Figure 4: The non-trivial chemical rooted trees $T_v^\dagger$ for $v \in \{u_5, u_{12}, u_{23}\} = V_B^*$ and the non-trivial chemical bi-rooted trees $T_a^\dagger$ for $a \in \{a_1 = u_1u_2, a_2 = u_1u_3, a_3 = u_4u_7, a_4 = u_{10}u_{11}, a_5 = u_{11}u_{12}\} = E_B^*$ for the $(\sigma_{\rm int}, \sigma_{\rm ce})$-extension $G^\dagger = G$ in Figure 2, where the gray squares indicate the roots of these rooted and bi-rooted trees.

3 All Constraints in an MILP Formulation for Chemical Graphs

We define a standard encoding of a finite set A of elements to be a bijection $\sigma : A \to [1, |A|]$, where we denote by $[A]$ the set $[1, |A|]$ of integers and by $[\mathtt{e}]$ the encoded element $\sigma(\mathtt{e})$. Let ϵ denote *null*, a fictitious chemical element that does not belong to any set of chemical elements, chemical symbols, adjacency-configurations and edge-configurations in the following formulation. Given a finite set A, let A_ϵ denote the set $A \cup \{\epsilon\}$ and define a standard encoding of A_ϵ to be a bijection $\sigma : A \to [0, |A|]$ such that $\sigma(\epsilon) = 0$, where we denote by $[A_\epsilon]$ the set $[0, |A|]$ of integers and by $[\mathtt{e}]$ the encoded element $\sigma(\mathtt{e})$, where $[\epsilon] = 0$.

3.1 Selecting a Cyclical-base

Recall that

$$E_{(=1)} = \{e \in E_{\rm C} \mid \ell_{\rm LB}(e) = \ell_{\rm UB}(e) = 1\}; \quad E_{(0/1)} = \{e \in E_{\rm C} \mid \ell_{\rm LB}(e) = 0, \ell_{\rm UB}(e) = 1\};$$
$$E_{(\geq 1)} = \{e \in E_{\rm C} \mid \ell_{\rm LB}(e) = 1, \ell_{\rm UB}(e) \geq 2\}; \quad E_{(\geq 2)} = \{e \in E_{\rm C} \mid \ell_{\rm LB}(e) \geq 2\};$$

- Every edge $a_i \in E_{(=1)}$ is included in G;

- Each edge $a_i \in E_{(0/1)}$ is included in G if necessary;

- For each edge $a_i \in E_{(\geq 2)}$, edge a_i is not included in G and instead a path

$$P_i = ({v^{\rm C}}_{{\rm tail}(i)}, {v^{\rm T}}_{j-1}, {v^{\rm T}}_j, \ldots, {v^{\rm T}}_{j+t}, {v^{\rm C}}_{{\rm head}(i)})$$

  of length at least 2 from vertex ${v^{\rm C}}_{{\rm tail}(i)}$ to vertex ${v^{\rm C}}_{{\rm head}(i)}$ visiting some vertices in $V_{\rm T}$ is constructed in G; and

- For each edge $a_i \in E_{(\geq 1)}$, either edge a_i is directly used in G or the above path P_i of length at least 2 is constructed in G.

Let $t_{\rm C} \triangleq |V_{\rm C}|$ and denote $V_{\rm C}$ by $\{v^{\rm C}{}_i \mid i \in [1, t_{\rm C}]\}$. Regard the seed graph $G_{\rm C}$ as a digraph such that each edge a_i with end-vertices $v^{\rm C}{}_j$ and $v^{\rm C}{}_{j'}$ is directed from $v^{\rm C}{}_j$ to $v^{\rm C}{}_{j'}$ when $j < j'$. For each directed edge $a_i \in E_{\rm C}$, let head(i) and tail(i) denote the head and tail of $e^{\rm C}(i)$; i.e., $a_i = (v^{\rm C}{}_{{\rm tail}(i)}, v^{\rm C}{}_{{\rm head}(i)})$.

Assume that $E_{\rm C} = \{a_i \mid i \in [1, m_{\rm C}]\}$, $E_{(\geq 2)} = \{a_k \mid k \in [1, p]\}$, $E_{(\geq 1)} = \{a_k \mid k \in [p+1, q]\}$, $E_{(0/1)} = \{a_i \mid i \in [q+1, t]\}$ and $E_{(=1)} = \{a_i \mid i \in [t+1, m_{\rm C}]\}$ for integers p, q and t. Let $I_{(=1)}$ denote the set of indices i of edges $a_i \in E_{(=1)}$. Similarly for $I_{(0/1)}$, $I_{(\geq 1)}$ and $I_{(\geq 2)}$.

Define

$$k_{\rm C} \triangleq |E_{(\geq 2)} \cup E_{(\geq 1)}|, \quad \widetilde{k_{\rm C}} \triangleq |E_{(\geq 2)}|.$$

To control the construction of such a path P_i for each edge $a_k \in E_{(\geq 2)} \cup E_{(\geq 1)}$, we regard the index $k \in [1, k_{\rm C}]$ of each edge $a_k \in E_{(\geq 2)} \cup E_{(\geq 1)}$ as the "color" of the edge. To introduce necessary linear constraints that can construct such a path P_k properly in our MILP, we assign the color k to the vertices $v^{\rm T}{}_{j-1}, v^{\rm T}{}_j, \ldots, v^{\rm T}{}_{j+t}$ in $V_{\rm T}$ when the above path P_k is used in G.

For each index $s \in [1, t_{\rm C}]$, let $I_{\rm C}(s)$ denote the set of edges $e \in E_{\rm C}$ incident to vertex $v^{\rm C}{}_s$, and $E^+_{(=1)}(s)$ (resp., $E^-_{(=1)}(s)$) denote the set of edges $a_i \in E_{(=1)}$ such that the tail (resp., head) of a_i is vertex $v^{\rm C}{}_s$. Similarly for $E^+_{(0/1)}(s)$, $E^-_{(0/1)}(s)$, $E^+_{(\geq 1)}(s)$, $E^-_{(\geq 1)}(s)$, $E^+_{(\geq 2)}(s)$ and $E^-_{(\geq 2)}(s)$. Let $I_{\rm C}(s)$ denote the set of indices i of edges $a_i \in I_{\rm C}(s)$. Similarly for $I^+_{(=1)}(s)$, $I^-_{(=1)}(s)$, $I^+_{(0/1)}(s)$, $I^-_{(0/1)}(s)$, $I^+_{(\geq 1)}(s)$, $I^-_{(\geq 1)}(s)$, $I^+_{(\geq 2)}(s)$ and $I^-_{(\geq 2)}(s)$. Note that $[1, k_{\rm C}] = I_{(\geq 2)} \cup I_{(\geq 1)}$ and $[\widetilde{k_{\rm C}} + 1, m_{\rm C}] = I_{(\geq 1)} \cup I_{(0/1)} \cup I_{(=1)}$.

constants:

- $t_{\rm C} = |V_{\rm C}|$, $\widetilde{k_{\rm C}} = |E_{(\geq 2)}|$, $k_{\rm C} = |E_{(\geq 2)} \cup E_{(\geq 1)}|$, $t_{\rm T} = {\rm n}^{\rm int}_{\rm UB} - |V_{\rm C}|$, $m_{\rm C} = |E_{\rm C}|$. Note that $a_i \in E_{\rm C} \setminus (E_{(\geq 2)} \cup E_{(\geq 1)})$ holds $i \in [k_{\rm C} + 1, m_{\rm C}]$;
- $\ell_{\rm LB}(k), \ell_{\rm UB}(k) \in [1, t_{\rm T}]$, $k \in [1, k_{\rm C}]$: lower and upper bounds on the length of path P_k;

variables:

- $e^{\rm C}(i) \in [0, 1]$, $i \in [1, m_{\rm C}]$: $e^{\rm C}(i)$ represents edge $a_i \in E_{\rm C}$, $i \in [1, m_{\rm C}]$ ($e^{\rm C}(i) = 1$, $i \in I_{(=1)}$; $e^{\rm C}(i) = 0$, $i \in I_{(\geq 2)}$) ($e^{\rm C}(i) = 1 \Leftrightarrow$ edge a_i is used in G);
- $v^{\rm T}(i) \in [0, 1]$, $i \in [1, t_{\rm T}]$: $v^{\rm T}(i) = 1 \Leftrightarrow$ vertex $v^{\rm T}{}_i$ is used in G;
- $e^{\rm T}(i) \in [0, 1]$, $i \in [1, t_{\rm T} + 1]$: $e^{\rm T}(i)$ represents edge $e^{\rm T}{}_i = (v^{\rm T}{}_{i-1}, v^{\rm T}{}_i) \in E_{\rm T}$, where $e^{\rm T}{}_1$ and $e^{\rm T}{}_{t_{\rm T}+1}$ are fictitious edges ($e^{\rm T}(i) = 1 \Leftrightarrow$ edge $e^{\rm T}{}_i$ is used in G);
- $\chi^{\rm T}(i) \in [0, k_{\rm C}]$, $i \in [1, t_{\rm T}]$: $\chi^{\rm T}(i)$ represents the color assigned to vertex $v^{\rm T}{}_i$ ($\chi^{\rm T}(i) = k > 0 \Leftrightarrow$ vertex $v^{\rm T}{}_i$ is assigned color k; $\chi^{\rm T}(i) = 0$ means that vertex $v^{\rm T}{}_i$ is not used in G);
- ${\rm clr}^{\rm T}(k) \in [\ell_{\rm LB}(k) - 1, \ell_{\rm UB}(k) - 1]$, $k \in [1, k_{\rm C}]$, ${\rm clr}^{\rm T}(0) \in [0, t_{\rm T}]$: the number of vertices $v^{\rm T}{}_i \in V_{\rm T}$ with color c;
- $\delta^{\rm T}_\chi(k) \in [0, 1]$, $k \in [0, k_{\rm C}]$: $\delta^{\rm T}_\chi(k) = 1 \Leftrightarrow \chi^{\rm T}(i) = k$ for some $i \in [1, t_{\rm T}]$;
- $\chi^{\rm T}(i, k) \in [0, 1]$, $i \in [1, t_{\rm T}]$, $k \in [0, k_{\rm C}]$ ($\chi^{\rm T}(i, k) = 1 \Leftrightarrow \chi^{\rm T}(i) = k$);
- $\widetilde{\rm deg}^+_{\rm C}(i) \in [0, 4]$, $i \in [1, t_{\rm C}]$: the out-degree of vertex $v^{\rm C}{}_i$ with the used edges $e^{\rm C}$ in $E_{\rm C}$;
- $\widetilde{\rm deg}^-_{\rm C}(i) \in [0, 4]$, $i \in [1, t_{\rm C}]$: the in-degree of vertex $v^{\rm C}{}_i$ with the used edges $e^{\rm C}$ in $E_{\rm C}$;

constraints:

$$e^{\rm C}(i) = 1, \quad i \in I_{(=1)}, \tag{1}$$

$$e^{\rm C}(i) = 0, \quad {\rm clr}^{\rm T}(i) \geq 1, \quad i \in I_{(\geq 2)}, \tag{2}$$

$$e^{\rm C}(i) + {\rm clr}^{\rm T}(i) \geq 1, \qquad {\rm clr}^{\rm T}(i) \leq t_{\rm T} \cdot (1 - e^{\rm C}(i)), \quad i \in I_{(\geq 1)}, \tag{3}$$

$$\sum_{c \in I^-_{(\geq 1)}(i) \cup I^-_{(0/1)}(i) \cup I^-_{(=1)}(i)} e^{\rm C}(c) = \widetilde{\deg}^-_{\rm C}(i), \qquad \sum_{c \in I^+_{(\geq 1)}(i) \cup I^+_{(0/1)}(i) \cup I^+_{(=1)}(i)} e^{\rm C}(c) = \widetilde{\deg}^+_{\rm C}(i), \quad i \in [1, t_{\rm C}], \tag{4}$$

$$\chi^{\rm T}(i, 0) = 1 - v^{\rm T}(i), \qquad \sum_{k \in [0, k_{\rm C}]} \chi^{\rm T}(i, k) = 1, \qquad \sum_{k \in [0, k_{\rm C}]} k \cdot \chi^{\rm T}(i, k) = \chi^{\rm T}(i), \qquad i \in [1, t_{\rm T}], \tag{5}$$

$$\sum_{i \in [1, t_{\rm T}]} \chi^{\rm T}(i, k) = {\rm clr}^{\rm T}(k), \quad t_{\rm T} \cdot \delta^{\rm T}_\chi(k) \geq \sum_{i \in [1, t_{\rm T}]} \chi^{\rm T}(i, k) \geq \delta^{\rm T}_\chi(k), \qquad k \in [0, k_{\rm C}], \tag{6}$$

$$\begin{aligned} v^{\rm T}(i-1) &\geq v^{\rm T}(i), \\ k_{\rm C} \cdot (v^{\rm T}(i-1) - e^{\rm T}(i)) \geq \chi^{\rm T}(i-1) - \chi^{\rm T}(i) &\geq v^{\rm T}(i-1) - e^{\rm T}(i), \qquad i \in [2, t_{\rm T}]. \end{aligned} \tag{7}$$

3.2 Constraints for Including Leaf Paths

Let $\widetilde{t_{\rm C}}$ denote the number of vertices $u \in V_{\rm C}$ such that ${\rm bl}_{\rm UB}(u) = 1$ and assume that $V_{\rm C} = \{u_1, u_2, \ldots, u_p\}$ so that

$${\rm bl}_{\rm UB}(u_i) = 1,\ i \in [1, \widetilde{t_{\rm C}}] \text{ and } {\rm bl}_{\rm UB}(u_i) = 0,\ i \in [\widetilde{t_{\rm C}} + 1, t_{\rm C}].$$

Define the set of colors for the vertex set $\{u_i \mid i \in [1, \widetilde{t_{\rm C}}]\} \cup V_{\rm T}$ to be $[1, c_{\rm F}]$ with

$$c_{\rm F} \triangleq \widetilde{t_{\rm C}} + t_{\rm T} = |\{u_i \mid i \in [1, \widetilde{t_{\rm C}}]\} \cup V_{\rm T}|.$$

Let each vertex ${v^{\rm C}}_i$, $i \in [1, \widetilde{t_{\rm C}}]$ (resp., ${v^{\rm T}}_i \in V_{\rm T}$) correspond to a color $i \in [1, c_{\rm F}]$ (resp., $i + \widetilde{t_{\rm C}} \in [1, c_{\rm F}]$). When a path $P = (u, {v^{\rm F}}_j, {v^{\rm F}}_{j+1}, \ldots, {v^{\rm F}}_{j+t})$ from a vertex $u \in V_{\rm C} \cup V_{\rm T}$ is used in G, we assign the color $i \in [1, c_{\rm F}]$ of the vertex u to the vertices ${v^{\rm F}}_j, {v^{\rm F}}_{j+1}, \ldots, {v^{\rm F}}_{j+t} \in V_{\rm F}$.

constants:

- $c_{\rm F}$: the maximum number of different colors assigned to the vertices in $V_{\rm F}$;
- ${\rm n}^{\rm int}_{\rm LB}, {\rm n}^{\rm int}_{\rm UB} \in [2, n^*]$: lower and upper bounds on the number of interior-vertices in G;
- ${\rm bl}_{\rm LB}(i) \in [0, 1]$, $i \in [1, \widetilde{t_{\rm C}}]$: a lower bound on the number of leaf ρ-branches in the leaf path rooted at a vertex ${v^{\rm C}}_i$;
- ${\rm bl}_{\rm LB}(k), {\rm bl}_{\rm UB}(k) \in [0, \ell_{\rm UB}(k) - 1]$, $k \in [1, k_{\rm C}] = I_{(\geq 2)} \cup I_{(\geq 1)}$: lower and upper bounds on the number of leaf ρ-branches in the trees rooted at internal vertices of a pure path P_k for an edge $a_k \in E_{(\geq 1)} \cup E_{(\geq 2)}$;

variables:

- $\mathrm{n}^{\mathrm{int}}_G \in [\mathrm{n}^{\mathrm{int}}_{\mathrm{LB}}, \mathrm{n}^{\mathrm{int}}_{\mathrm{UB}}]$: the number of interior-vertices in G;
- $v^{\mathrm{F}}(i) \in [0,1]$, $i \in [1, t_{\mathrm{F}}]$: $v^{\mathrm{F}}(i) = 1 \Leftrightarrow$ vertex ${v^{\mathrm{F}}}_i$ is used in G;
- $e^{\mathrm{F}}(i) \in [0,1]$, $i \in [1, t_{\mathrm{F}}+1]$: $e^{\mathrm{F}}(i)$ represents edge ${e^{\mathrm{F}}}_i = {v^{\mathrm{F}}}_{i-1}{v^{\mathrm{F}}}_i$, where ${e^{\mathrm{F}}}_1$ and ${e^{\mathrm{F}}}_{t_{\mathrm{F}}+1}$ are fictitious edges ($e^{\mathrm{F}}(i) = 1 \Leftrightarrow$ edge ${e^{\mathrm{F}}}_i$ is used in G);
- $\chi^{\mathrm{F}}(i) \in [0, c_{\mathrm{F}}]$, $i \in [1, t_{\mathrm{F}}]$: $\chi^{\mathrm{F}}(i)$ represents the color assigned to vertex ${v^{\mathrm{F}}}_i$ ($\chi^{\mathrm{F}}(i) = c \Leftrightarrow$ vertex ${v^{\mathrm{F}}}_i$ is assigned color c);
- $\mathrm{clr}^{\mathrm{F}}(c) \in [0, t_{\mathrm{F}}]$, $c \in [0, c_{\mathrm{F}}]$: the number of vertices ${v^{\mathrm{F}}}_i$ with color c;
- $\delta^{\mathrm{F}}_{\chi}(c) \in [\mathrm{bl}_{\mathrm{LB}}(c), 1]$, $c \in [1, \widetilde{t_{\mathrm{C}}}]$: $\delta^{\mathrm{F}}_{\chi}(c) = 1 \Leftrightarrow \chi^{\mathrm{F}}(i) = c$ for some $i \in [1, t_{\mathrm{F}}]$;
- $\delta^{\mathrm{F}}_{\chi}(c) \in [0,1]$, $c \in [\widetilde{t_{\mathrm{C}}}+1, c_{\mathrm{F}}]$: $\delta^{\mathrm{F}}_{\chi}(c) = 1 \Leftrightarrow \chi^{\mathrm{F}}(i) = c$ for some $i \in [1, t_{\mathrm{F}}]$;
- $\chi^{\mathrm{F}}(i,c) \in [0,1]$, $i \in [1, t_{\mathrm{F}}]$, $c \in [0, c_{\mathrm{F}}]$: $\chi^{\mathrm{F}}(i,c) = 1 \Leftrightarrow \chi^{\mathrm{F}}(i) = c$;
- $\mathrm{bl}(k,i) \in [0,1]$, $k \in [1, k_{\mathrm{C}}] = I_{(\geq 2)} \cup I_{(\geq 1)}$, $i \in [1, t_{\mathrm{T}}]$: $\mathrm{bl}(k,i) = 1 \Leftrightarrow$ path P_k contains vertex ${v^{\mathrm{T}}}_i$ as an internal vertex and the ρ-fringe-tree rooted at ${v^{\mathrm{T}}}_i$ contains a leaf ρ-branch;

constraints:

$$\chi^{\mathrm{F}}(i,0) = 1 - v^{\mathrm{F}}(i), \quad \sum_{c \in [0, c_{\mathrm{F}}]} \chi^{\mathrm{F}}(i,c) = 1, \quad \sum_{c \in [0, c_{\mathrm{F}}]} c \cdot \chi^{\mathrm{F}}(i,c) = \chi^{\mathrm{F}}(i), \qquad i \in [1, t_{\mathrm{F}}], \tag{8}$$

$$\sum_{i \in [1, t_{\mathrm{F}}]} \chi^{\mathrm{F}}(i,c) = \mathrm{clr}^{\mathrm{F}}(c), \quad t_{\mathrm{F}} \cdot \delta^{\mathrm{F}}_{\chi}(c) \geq \sum_{i \in [1, t_{\mathrm{F}}]} \chi^{\mathrm{F}}(i,c) \geq \delta^{\mathrm{F}}_{\chi}(c), \qquad c \in [0, c_{\mathrm{F}}], \tag{9}$$

$$e^{\mathrm{F}}(1) = e^{\mathrm{F}}(t_{\mathrm{F}}+1) = 0, \tag{10}$$

$$\begin{aligned} v^{\mathrm{F}}(i-1) &\geq v^{\mathrm{F}}(i), \\ c_{\mathrm{F}} \cdot (v^{\mathrm{F}}(i-1) - e^{\mathrm{F}}(i)) \geq \chi^{\mathrm{F}}(i-1) - \chi^{\mathrm{F}}(i) &\geq v^{\mathrm{F}}(i-1) - e^{\mathrm{F}}(i), \end{aligned} \qquad i \in [2, t_{\mathrm{F}}], \tag{11}$$

$$\mathrm{bl}(k,i) \geq \delta^{\mathrm{F}}_{\chi}(\widetilde{t_{\mathrm{C}}}+i) + \chi^{\mathrm{T}}(i,k) - 1, \qquad k \in [1, k_{\mathrm{C}}], i \in [1, t_{\mathrm{T}}], \tag{12}$$

$$\sum_{k \in [1, k_{\mathrm{C}}], i \in [1, t_{\mathrm{T}}]} \mathrm{bl}(k,i) \leq \sum_{i \in [1, t_{\mathrm{T}}]} \delta^{\mathrm{F}}_{\chi}(\widetilde{t_{\mathrm{C}}}+i), \tag{13}$$

$$\mathrm{bl}_{\mathrm{LB}}(k) \leq \sum_{i \in [1, t_{\mathrm{T}}]} \mathrm{bl}(k,i) \leq \mathrm{bl}_{\mathrm{UB}}(k), \qquad k \in [1, k_{\mathrm{C}}], \tag{14}$$

$$t_{\mathrm{C}} + \sum_{i \in [1, t_{\mathrm{T}}]} v^{\mathrm{T}}(i) + \sum_{i \in [1, t_{\mathrm{F}}]} v^{\mathrm{F}}(i) = \mathrm{n}^{\mathrm{int}}_G. \tag{15}$$

3.3 Constraints for Including Fringe-trees

To express the condition that the ρ-fringe-tree is chosen from a rooted tree C_i, T_i or F_i, we introduce the following set of variables and constraints.

constants:

- $n_{\rm LB}, n^*$: lower and upper bounds on $n(G)$, where $n_{\rm LB}, n^* \geq {\rm n}^{\rm int}_{\rm LB}$;
- ${\rm ch}_{\rm LB}(i), {\rm ch}_{\rm UB}(i) \in [0, n^*]$, $i \in [1, t_{\rm T}]$: lower and upper bounds on ${\rm ht}(T_i)$ of the tree T_i rooted at a vertex $v^{\rm C}{}_i$;
- ${\rm ch}_{\rm LB}(k), {\rm ch}_{\rm UB}(k) \in [0, n^*]$, $k \in [1, k_{\rm C}] = I_{(\geq 2)} \cup I_{(\geq 1)}$: lower and upper bounds on the maximum height ${\rm ht}(T)$ of the tree $T \in \mathcal{F}(P_k)$ rooted at an internal vertex of a path P_k for an edge $a_k \in E_{(\geq 1)} \cup E_{(\geq 2)}$;
- Let $\mathcal{F}_\Lambda$ denote the set of chemical rooted trees $\psi = (\{v\}, \emptyset)$ with ${\rm ht}(\psi) = 0$ and $\alpha(v) = \mathtt{a}$ for each chemical element $\mathtt{a} \in \Lambda$;
- Prepare a coding of the set $\mathcal{F}(D_\pi)$ and let $[\psi]$ denote the coded integer of an element ψ in $\mathcal{F}(D_\pi)$;
- Sets $\mathcal{F}(v) \subseteq \mathcal{F}(D_\pi), v \in V_{\rm C}$ and $\mathcal{F}_E \subseteq \mathcal{F}(D_\pi)$ of chemical rooted trees T with ${\rm ht}(T) \in [1, \rho]$;
- Define $\mathcal{F}^* := \bigcup_{v \in V_{\rm C}} \mathcal{F}(v) \cup \mathcal{F}_E$, $\mathcal{F}^{\rm C}_i := \mathcal{F}(v^{\rm C}{}_i)$, $i \in [1, t_{\rm C}]$, $\mathcal{F}^{\rm T}_i := \mathcal{F}_E$, $i \in [1, t_{\rm T}]$ and $\mathcal{F}^{\rm F}_i := \mathcal{F}_E$, $i \in [1, t_{\rm F}]$;
- $\mathcal{F}^{\rm X}_i[p], p \in [1, \rho], {\rm X} \in \{{\rm C}, {\rm T}, {\rm F}\}$: the set of chemical rooted trees $T \in \mathcal{F}^{\rm X}_i$ with ${\rm ht}(T) = p$;
- $n([\psi]) \in [0, 3^\rho], \psi \in \mathcal{F}^*$: the number of non-root vertices in a chemical rooted tree ψ;
- ${\rm ht}([\psi]) \in [0, \rho], \psi \in \mathcal{F}^*$: the height of a chemical rooted tree ψ;
- ${\rm deg}_{\rm r}([\psi]) \in [0, 4], \psi \in \mathcal{F}^*$: the number of children of the root r of a chemical rooted tree ψ;

variables:

- $n_G \in [n_{\rm LB}, n^*]$: $n(G)$;
- $v^{\rm X}(i) \in [0, 1], i \in [1, t_{\rm X}]$, ${\rm X} \in \{{\rm T}, {\rm F}\}$: $v^{\rm X}(i) = 1 \Leftrightarrow$ vertex $v^{\rm X}{}_i$ is used in G;
- $h^{\rm X}(i) \in [0, \rho]$, $i \in [1, t_{\rm X}]$, ${\rm X} \in \{{\rm C}, {\rm T}, {\rm F}\}$: the height of the ρ-fringe-tree rooted at vertex $v^{\rm X}{}_i$ in G;
- $\delta^{\rm X}_{\rm fr}(i, [\psi]) \in [0, 1], i \in [1, t_{\rm X}], \psi \in \mathcal{F}^{\rm X}_i \cup \mathcal{F}_\Lambda, {\rm X} \in \{{\rm T}, {\rm F}\}$: $\delta^{\rm X}_{\rm fr}(i, [\psi]) = 1 \Leftrightarrow \psi$ is the ρ-fringe-tree at vertex $v^{\rm X}{}_i$, where $\psi \in \mathcal{F}_\Lambda$ means that the height of the ρ-fringe-tree is 0;
- ${\rm deg}^{\rm ex}_{\rm X}(i) \in [0, 3], i \in [1, t_{\rm X}], {\rm X} \in \{{\rm C}, {\rm T}, {\rm F}\}$: the number of children of the root of the ρ-fringe-tree rooted at vertex $v^{\rm X}{}_i$ in G;
- $\sigma(k, i) \in [0, 1]$, $k \in [1, k_{\rm C}] = I_{(\geq 2)} \cup I_{(\geq 1)}, i \in [1, t_{\rm T}]$: $\sigma(k, i) = 1 \Leftrightarrow$ the ρ-fringe-tree T_v rooted at vertex $v = v^{\rm T}{}_i$ with color k has the largest height among such trees;

constraints:

$$\sum_{\psi \in \mathcal{F}^{\rm C}_i \cup \mathcal{F}_\Lambda} \delta^{\rm C}_{\rm fr}(i, [\psi]) = 1, \quad \sum_{\psi \in \mathcal{F}^{\rm C}_i \cup \mathcal{F}_\Lambda} {\rm deg}_{\rm r}([\psi]) \cdot \delta^{\rm C}_{\rm fr}(i, [\psi]) = {\rm deg}^{\rm ex}_{\rm C}(i), \qquad i \in [1, t_{\rm C}],$$

$$\sum_{\psi \in \mathcal{F}^{\rm X}_i \cup \mathcal{F}_\Lambda} \delta^{\rm X}_{\rm fr}(i, [\psi]) = v^{\rm X}(i), \quad \sum_{\psi \in \mathcal{F}^{\rm X}_i \cup \mathcal{F}_\Lambda} {\rm deg}_{\rm r}([\psi]) \cdot \delta^{\rm X}_{\rm fr}(i, [\psi]) = {\rm deg}^{\rm ex}_{\rm X}(i), \quad i \in [1, t_{\rm X}], {\rm X} \in \{{\rm T}, {\rm F}\}, \quad (16)$$

$$\sum_{\psi\in\mathcal{F}_i^{\rm F}[\rho]} \delta_{\rm fr}^{\rm F}(i,[\psi]) \geq v^{\rm F}(i) - e^{\rm F}(i+1), \qquad i\in[1,t_{\rm F}]\ (e^{\rm F}(t_{\rm F}+1)=0), \tag{17}$$

$$\sum_{\psi\in\mathcal{F}_i^{\rm X}} {\rm ht}([\psi])\cdot\delta_{\rm fr}^{\rm X}(i,[\psi]) = h^{\rm X}(i), \qquad i\in[1,t_{\rm X}], {\rm X}\in\{{\rm C},{\rm T},{\rm F}\}, \tag{18}$$

$$\sum_{\substack{\psi\in\mathcal{F}_i^{\rm X}\\ i\in[1,t_{\rm X}],{\rm X}\in\{{\rm C},{\rm T},{\rm F}\}}} n([\psi])\cdot\delta_{\rm fr}^{\rm X}(i,[\psi]) + \sum_{i\in[1,t_{\rm X}],{\rm X}\in\{{\rm T},{\rm F}\}} v^{\rm X}(i) + t_{\rm C} = n_G, \tag{19}$$

$$\begin{aligned} h^{\rm C}(i) \geq {\rm ch}_{\rm LB}(i) - n^*\delta_\chi^{\rm F}(i), \quad {\rm clr}^{\rm F}(i)+\rho \geq {\rm ch}_{\rm LB}(i), \\ h^{\rm C}(i) \leq {\rm ch}_{\rm UB}(i), \quad {\rm clr}^{\rm F}(i)+\rho \leq {\rm ch}_{\rm UB}(i) + n^*(1-\delta_\chi^{\rm F}(i)),\ i\in[1,\widetilde{t_{\rm C}}], \end{aligned} \tag{20}$$

$${\rm ch}_{\rm LB}(i) \leq h^{\rm C}(i) \leq {\rm ch}_{\rm UB}(i), \quad i\in[\widetilde{t_{\rm C}}+1, t_{\rm C}], \tag{21}$$

$$\begin{aligned} h^{\rm T}(i) \leq {\rm ch}_{\rm UB}(k) + n^*(\delta_\chi^{\rm F}(\widetilde{t_{\rm C}}+i) + 1 - \chi^{\rm T}(i,k)), \\ {\rm clr}^{\rm F}(\widetilde{t_{\rm C}}+i)+\rho \leq {\rm ch}_{\rm UB}(k) + n^*(2-\delta_\chi^{\rm F}(\widetilde{t_{\rm C}}+i) - \chi^{\rm T}(i,k)), \\ k\in[1,k_{\rm C}], i\in[1,t_{\rm T}], \end{aligned} \tag{22}$$

$$\sum_{i\in[1,t_{\rm T}]} \sigma(k,i) = \delta_\chi^{\rm T}(k), \qquad k\in[1,k_{\rm C}], \tag{23}$$

$$\begin{aligned} \chi^{\rm T}(i,k) \geq \sigma(k,i), \\ h^{\rm T}(i) \geq {\rm ch}_{\rm LB}(k) - n^*(\delta_\chi^{\rm F}(\widetilde{t_{\rm C}}+i) + 1 - \sigma(k,i)), \\ {\rm clr}^{\rm F}(\widetilde{t_{\rm C}}+i)+\rho \geq {\rm ch}_{\rm LB}(k) - n^*(2-\delta_\chi^{\rm F}(\widetilde{t_{\rm C}}+i) - \sigma(k,i)), \qquad k\in[1,k_{\rm C}], i\in[1,t_{\rm T}]. \end{aligned} \tag{24}$$

3.4 Descriptor for the Number of Specified Degree

We include constraints to compute descriptors ${\rm dg}_d^{\rm int}(G)$, $d\in[1,4]$.

variables:

- ${\rm deg}^{\rm X}(i)\in[0,4]$, $i\in[1,t_{\rm X}]$, ${\rm X}\in\{{\rm C},{\rm T},{\rm F}\}$: the degree ${\rm deg}_G(v^{\rm X}{}_i)$ of vertex $v^{\rm X}{}_i$ in G;
- ${\rm deg}_{\rm CT}(i)\in[0,4]$, $i\in[1,t_{\rm C}]$: the number of edges from vertex $v^{\rm C}{}_i$ to vertices $v^{\rm T}{}_j$, $j\in[1,t_{\rm T}]$;
- ${\rm deg}_{\rm TC}(i)\in[0,4]$, $i\in[1,t_{\rm C}]$: the number of edges from vertices $v^{\rm T}{}_j$, $j\in[1,t_{\rm T}]$ to vertex $v^{\rm C}{}_i$;

- $\delta_{\rm dg}^{\rm C}(i,d) \in [0,1]$, $i \in [1,t_{\rm C}]$, $d \in [1,4]$, $\delta_{\rm dg}^{\rm X}(i,d) \in [0,1]$, $i \in [1,t_{\rm X}]$, $d \in [0,4]$, ${\rm X} \in \{{\rm T},{\rm F}\}$: $\delta_{\rm dg}^{\rm X}(i,d) = 1 \Leftrightarrow {\rm deg}^{\rm X}(i) = d$;
- ${\rm dg}(d) \in [{\rm dg}_{\rm LB}(d), {\rm dg}_{\rm UB}(d)]$, $d \in [1,4]$: the number of interior-vertices v with ${\rm deg}_G(v) = d$;
- ${\rm deg}_{\rm C}^{\rm int}(i) \in [1,4]$, $i \in [1,t_{\rm C}]$, ${\rm deg}_{\rm X}^{\rm int}(i) \in [0,4]$, $i \in [1,t_{\rm X}], {\rm X} \in \{{\rm T},{\rm F}\}$: the interior-degree ${\rm deg}_{(V^{\rm int},E^{\rm int})}(v^{\rm X}{}_i)$; i.e., the number of interior-edges incident to vertex $v^{\rm X}{}_i$;
- $\delta_{\rm dg,C}^{\rm int}(i,d) \in [0,1]$, $i \in [1,t_{\rm C}]$, $d \in [1,4]$, $\delta_{\rm dg,X}^{\rm int}(i,d) \in [0,1]$, $i \in [1,t_{\rm X}]$, $d \in [0,4]$, ${\rm X} \in \{{\rm T},{\rm F}\}$: $\delta_{\rm dg,X}^{\rm int}(i,d) = 1 \Leftrightarrow {\rm deg}_{\rm X}^{\rm int}(i) = d$;
- ${\rm dg}^{\rm int}(d) \in [{\rm dg}_{\rm LB}(d), {\rm dg}_{\rm UB}(d)]$, $d \in [1,4]$: the number of interior-vertices v with the interior-degree ${\rm deg}_{(V^{\rm int},E^{\rm int})}(v) = d$;

constraints:

$$\sum_{k \in I^+_{(\geq 2)}(i) \cup I^+_{(\geq 1)}(i)} \delta_\chi^{\rm T}(k) = {\rm deg}_{\rm CT}(i), \quad \sum_{k \in I^-_{(\geq 2)}(i) \cup I^-_{(\geq 1)}(i)} \delta_\chi^{\rm T}(k) = {\rm deg}_{\rm TC}(i), \qquad i \in [1,t_{\rm C}], \tag{25}$$

$$\widetilde{\rm deg}_{\rm C}^{-}(i) + \widetilde{\rm deg}_{\rm C}^{+}(i) + {\rm deg}_{\rm CT}(i) + {\rm deg}_{\rm TC}(i) + \delta_\chi^{\rm F}(i) = {\rm deg}_{\rm C}^{\rm int}(i), \qquad i \in [1,\widetilde{t_{\rm C}}], \tag{26}$$

$$\widetilde{\rm deg}_{\rm C}^{-}(i) + \widetilde{\rm deg}_{\rm C}^{+}(i) + {\rm deg}_{\rm CT}(i) + {\rm deg}_{\rm TC}(i) = {\rm deg}_{\rm C}^{\rm int}(i), \qquad i \in [\widetilde{t_{\rm C}}+1, t_{\rm C}], \tag{27}$$

$${\rm deg}_{\rm C}^{\rm int}(i) + {\rm deg}_{\rm C}^{\rm ex}(i) = {\rm deg}^{\rm C}(i), \qquad i \in [1,t_{\rm C}], \tag{28}$$

$$\sum_{\psi \in \mathcal{F}_i^{\rm C}[\rho]} \delta_{\rm fr}^{\rm C}(i,[\psi]) \geq 2 - {\rm deg}_{\rm C}^{\rm int}(i) \qquad i \in [1,t_{\rm C}], \tag{29}$$

$$\begin{aligned} 2v^{\rm T}(i) + \delta_\chi^{\rm F}(\widetilde{t_{\rm C}}+i) &= {\rm deg}_{\rm T}^{\rm int}(i), \\ {\rm deg}_{\rm T}^{\rm int}(i) + {\rm deg}_{\rm T}^{\rm ex}(i) &= {\rm deg}^{\rm T}(i), \qquad i \in [1,t_{\rm T}] \ (e^{\rm T}(1) = e^{\rm T}(t_{\rm T}+1) = 0), \end{aligned} \tag{30}$$

$$\begin{aligned} v^{\rm F}(i) + e^{\rm F}(i+1) &= {\rm deg}_{\rm F}^{\rm int}(i), \\ {\rm deg}_{\rm F}^{\rm int}(i) + {\rm deg}_{\rm F}^{\rm ex}(i) &= {\rm deg}^{\rm F}(i), \qquad i \in [1,t_{\rm F}] \ (e^{\rm F}(1) = e^{\rm F}(t_{\rm F}+1) = 0), \end{aligned} \tag{31}$$

$$\begin{aligned} &\sum_{d \in [0,4]} \delta_{\rm dg}^{\rm X}(i,d) = 1, \quad \sum_{d \in [1,4]} d \cdot \delta_{\rm dg}^{\rm X}(i,d) = {\rm deg}^{\rm X}(i), \\ &\sum_{d \in [0,4]} \delta_{\rm dg,X}^{\rm int}(i,d) = 1, \quad \sum_{d \in [1,4]} d \cdot \delta_{\rm dg,X}^{\rm int}(i,d) = {\rm deg}_{\rm X}^{\rm int}(i), \qquad i \in [1,t_{\rm X}], {\rm X} \in \{{\rm T},{\rm C},{\rm F}\}, \end{aligned} \tag{32}$$

$$\begin{aligned} &\sum_{i \in [1,t_{\rm C}]} \delta_{\rm dg}^{\rm C}(i,d) + \sum_{i \in [1,t_{\rm T}]} \delta_{\rm dg}^{\rm T}(i,d) + \sum_{i \in [1,t_{\rm F}]} \delta_{\rm dg}^{\rm F}(i,d) = {\rm dg}(d), \\ &\sum_{i \in [1,t_{\rm C}]} \delta_{\rm dg,C}^{\rm int}(i,d) + \sum_{i \in [1,t_{\rm T}]} \delta_{\rm dg,T}^{\rm int}(i,d) + \sum_{i \in [1,t_{\rm F}]} \delta_{\rm dg,F}^{\rm int}(i,d) = {\rm dg}^{\rm int}(d), \qquad d \in [1,4]. \end{aligned} \tag{33}$$

3.5 Assigning Multiplicity

We prepare an integer variable $\beta(e)$ for each edge e in the scheme graph SG to denote the bond-multiplicity of e in a selected graph G and include necessary constraints for the variables to satisfy in G.

constants:

- $\beta_{\rm r}([\psi])$: the sum of bond-multiplicities of edges incident to the root of a tree $\psi \in \mathcal{F}^*$;

variables:

- $\beta^{\rm X}(i) \in [0,3]$, $i \in [2, t_{\rm X}]$, ${\rm X} \in \{{\rm T}, {\rm F}\}$: the bond-multiplicity of edge $e^{\rm X}{}_i$;
- $\beta^{\rm C}(i) \in [0,3]$, $i \in [\widetilde{k_{\rm C}}+1, m_{\rm C}] = I_{(\geq 1)} \cup I_{(0/1)} \cup I_{(=1)}$: the bond-multiplicity of edge $a_i \in E_{(\geq 1)} \cup E_{(0/1)} \cup E_{(=1)}$;
- $\beta^{+}(k), \beta^{-}(k) \in [0,3]$, $k \in [1, k_{\rm C}] = I_{(\geq 2)} \cup I_{(\geq 1)}$: the bond-multiplicity of the first (resp., last) edge of the pure path P_k;
- $\beta^{\rm in}(c) \in [0,3]$, $c \in [1, c_{\rm F}]$: the bond-multiplicity of the first edge of the leaf path Q_c rooted at vertex c;
- $\beta^{\rm X}_{\rm ex}(i) \in [0,4], i \in [1, t_{\rm X}], {\rm X} \in \{{\rm C}, {\rm T}, {\rm F}\}$: the sum $\beta_{T_v}(v)$ of bond-multiplicities of edges in the ρ-fringe-tree T_v rooted at interior-vertex $v = v^{\rm X}{}_i$;
- $\delta^{\rm X}_{\beta}(i,m) \in [0,1]$, $i \in [2, t_{\rm X}]$, $m \in [0,3]$, ${\rm X} \in \{{\rm T}, {\rm F}\}$: $\delta^{\rm X}_{\beta}(i,m) = 1 \Leftrightarrow \beta^{\rm X}(i) = m$;
- $\delta^{\rm C}_{\beta}(i,m) \in [0,1]$, $i \in [\widetilde{k_{\rm C}}, m_{\rm C}] = I_{(\geq 1)} \cup I_{(0/1)} \cup I_{(=1)}$, $m \in [0,3]$: $\delta^{\rm C}_{\beta}(i,m) = 1 \Leftrightarrow \beta^{\rm C}(i) = m$;
- $\delta^{+}_{\beta}(k,m), \delta^{-}_{\beta}(k,m) \in [0,1]$, $k \in [1, k_{\rm C}] = I_{(\geq 2)} \cup I_{(\geq 1)}$, $m \in [0,3]$: $\delta^{+}_{\beta}(k,m) = 1$ (resp., $\delta^{-}_{\beta}(k,m) = 1$) $\Leftrightarrow \beta^{+}(k) = m$ (resp., $\beta^{-}(k) = m$);
- $\delta^{\rm in}_{\beta}(c,m) \in [0,1]$, $c \in [1, c_{\rm F}]$, $m \in [0,3]$: $\delta^{\rm in}_{\beta}(c,m) = 1 \Leftrightarrow \beta^{\rm in}(c) = m$;
- ${\rm bd}^{\rm int}(m) \in [0, 2{\rm n}^{\rm int}_{\rm UB}]$, $m \in [1,3]$: the number of interior-edges with bond-multiplicity m in G;
- ${\rm bd}_{\rm X}(m) \in [0, 2{\rm n}^{\rm int}_{\rm UB}], {\rm X} \in \{{\rm C}, {\rm T}, {\rm CT}, {\rm TC}\}$, ${\rm bd}_{\rm X}(m) \in [0, 2{\rm n}^{\rm int}_{\rm UB}], {\rm X} \in \{{\rm F}, {\rm CF}, {\rm TF}\}$, $m \in [1,3]$: the number of interior-edges $e \in E_{\rm X}$ with bond-multiplicity m in G;

constraints:

$$e^{\rm C}(i) \leq \beta^{\rm C}(i) \leq 3e^{\rm C}(i), i \in [\widetilde{k_{\rm C}}+1, m_{\rm C}] = I_{(\geq 1)} \cup I_{(0/1)} \cup I_{(=1)}, \tag{34}$$

$$e^{\rm X}(i) \leq \beta^{\rm X}(i) \leq 3e^{\rm X}(i), \qquad i \in [2, t_{\rm X}], {\rm X} \in \{{\rm T}, {\rm F}\}, \tag{35}$$

$$\delta^{\rm T}_{\chi}(k) \leq \beta^{+}(k) \leq 3\delta^{\rm T}_{\chi}(k), \quad \delta^{\rm T}_{\chi}(k) \leq \beta^{-}(k) \leq 3\delta^{\rm T}_{\chi}(k), \qquad k \in [1, k_{\rm C}], \tag{36}$$

$$\delta^{\rm F}_{\chi}(c) \leq \beta^{\rm in}(c) \leq 3\delta^{\rm F}_{\chi}(c), \qquad c \in [1, c_{\rm F}], \tag{37}$$

$$\sum_{m\in[0,3]} \delta_\beta^{\rm X}(i,m)=1, \quad \sum_{m\in[0,3]} m\cdot\delta_\beta^{\rm X}(i,m)=\beta^{\rm X}(i), \qquad i\in[2,t_{\rm X}], {\rm X}\in\{{\rm T},{\rm F}\}, \tag{38}$$

$$\sum_{m\in[0,3]} \delta_\beta^{\rm C}(i,m)=1, \quad \sum_{m\in[0,3]} m\cdot\delta_\beta^{\rm C}(i,m)=\beta^{\rm C}(i), \qquad i\in[\widetilde{k_{\rm C}}+1,m_{\rm C}], \tag{39}$$

$$\begin{aligned}
&\sum_{m\in[0,3]} \delta_\beta^{+}(k,m)=1, \quad \sum_{m\in[0,3]} m\cdot\delta_\beta^{+}(k,m)=\beta^{+}(k), && k\in[1,k_{\rm C}],\\
&\sum_{m\in[0,3]} \delta_\beta^{-}(k,m)=1, \quad \sum_{m\in[0,3]} m\cdot\delta_\beta^{-}(k,m)=\beta^{-}(k), && k\in[1,k_{\rm C}],\\
&\sum_{m\in[0,3]} \delta_\beta^{\rm in}(c,m)=1, \quad \sum_{m\in[0,3]} m\cdot\delta_\beta^{\rm in}(c,m)=\beta^{\rm in}(c), && c\in[1,c_{\rm F}],
\end{aligned} \tag{40}$$

$$\sum_{\psi\in\mathcal{F}_i^{\rm X}} \beta_{\rm r}([\psi])\cdot\delta_{\rm fr}^{\rm X}(i,[\psi])=\beta_{\rm ex}^{\rm X}(i), \qquad i\in[1,t_{\rm X}], {\rm X}\in\{{\rm C},{\rm T},{\rm F}\}, \tag{41}$$

$$\begin{aligned}
\sum_{i\in[\widetilde{k_{\rm C}}+1,m_{\rm C}]} \delta_\beta^{\rm C}(i,m)={\rm bd}_{\rm C}(m), \quad & \sum_{i\in[2,t_{\rm T}]} \delta_\beta^{\rm T}(i,m)={\rm bd}_{\rm T}(m),\\
\sum_{k\in[1,k_{\rm C}]} \delta_\beta^{+}(k,m)={\rm bd}_{\rm CT}(m), \quad & \sum_{k\in[1,k_{\rm C}]} \delta_\beta^{-}(k,m)={\rm bd}_{\rm TC}(m),\\
\sum_{i\in[2,t_{\rm F}]} \delta_\beta^{\rm F}(i,m)={\rm bd}_{\rm F}(m), \quad & \sum_{c\in[1,\widetilde{t_{\rm C}}]} \delta_\beta^{\rm in}(c,m)={\rm bd}_{\rm CF}(m),\\
& \sum_{c\in[\widetilde{t_{\rm C}}+1,c_{\rm F}]} \delta_\beta^{\rm in}(c,m)={\rm bd}_{\rm TF}(m),\\
{\rm bd}_{\rm C}(m)+{\rm bd}_{\rm T}(m)+{\rm bd}_{\rm F}(m)+{\rm bd}_{\rm CT}(m)+&{\rm bd}_{\rm TC}(m)+{\rm bd}_{\rm TF}(m)+{\rm bd}_{\rm CF}(m)={\rm bd}^{\rm int}(m),\\
& m\in[1,3].
\end{aligned} \tag{42}$$

3.6 Assigning Chemical Elements and Valence Condition

We include constraints so that each vertex u in a selected graph H satisfies the valence condition; i.e., $\sum_{uv\in E(H)} \beta(uv) \le {\rm val}(\alpha(u))$. With these constraints, a chemical graph $G = (H, \alpha, \beta)$ on a selected subgraph H will be constructed.

constants:

- Subsets $\Lambda^{\rm int}, \Lambda^{\rm ex} \subseteq \Lambda$ of chemical elements, where we denote by $[\mathtt{e}]$ (resp., $[\mathtt{e}]^{\rm int}$ and $[\mathtt{e}]^{\rm ex}$) of a standard encoding of an element $\mathtt{e}$ in the set Λ (resp., $\Lambda_\epsilon^{\rm int}$ and $\Lambda_\epsilon^{\rm ex}$);
- A valence function: ${\rm val} : \Lambda \to [1, 4]$;
- A function ${\rm mass}^* : \Lambda \to \mathbb{Z}$ (we let ${\rm mass}(\mathtt{a})$ denote the observed mass of a chemical element $\mathtt{a} \in \Lambda$, and define ${\rm mass}^*(\mathtt{a}) \triangleq \lfloor 10 \cdot {\rm mass}(\mathtt{a}) \rfloor$);

- Subsets $\Lambda^*(i) \subseteq \Lambda^{\rm int}$, $i \in [1, t_{\rm C}]$;
- $\mathrm{na_{LB}}(\mathtt{a}), \mathrm{na_{UB}}(\mathtt{a}) \in [0, n^*]$, $\mathtt{a} \in \Lambda$: lower and upper bounds on the number of vertices v with $\alpha(v) = \mathtt{a}$;
- $\mathrm{na^{int}_{LB}}(\mathtt{a}), \mathrm{na^{int}_{UB}}(\mathtt{a}) \in [0, n^*]$, $\mathtt{a} \in \Lambda^{\rm int}$: lower and upper bounds on the number of interior-vertices v with $\alpha(v) = \mathtt{a}$;
- $\alpha_{\rm r}([\psi]) \in [\Lambda^{\rm ex}], \in \mathcal{F}^* \cup \mathcal{F}_\Lambda$: the chemical element $\alpha(r)$ of the root r of ψ;
- $\mathrm{na^{ex}_{\mathtt{a}}}([\psi]) \in [0, n^*]$, $\mathtt{a} \in \Lambda^{\rm ex}, \psi \in \mathcal{F}^*$: the frequency of chemical element $\mathtt{a}$ in the set of non-rooted vertices in ψ;
- $n_{\mathtt{H}}([\psi], d) \in [0, 3^\rho], \psi \in \mathcal{F}^* \cup \mathcal{F}_\Lambda, d \in [0, 3]$: the number of non-root vertices with $\mathrm{deg_{hyd}}(v) = d$ in ψ.

variables:

- $\beta^{\rm CT}(i), \beta^{\rm TC}(i) \in [0, 3], i \in [1, t_{\rm T}]$: the bond-multiplicity of edge ${e^{\rm CT}}_{j,i}$ (resp., ${e^{\rm TC}}_{j,i}$) if one exists;
- $\beta^{\rm CF}(i), \beta^{\rm TF}(i) \in [0, 3], i \in [1, t_{\rm F}]$: the bond-multiplicity of ${e^{\rm CF}}_{j,i}$ (resp., ${e^{\rm TF}}_{j,i}$) if one exists;
- $\alpha^{\rm X}(i) \in [\Lambda^{\rm int}_\epsilon], \delta^{\rm X}_\alpha(i, [\mathtt{a}]^{\rm int}) \in [0, 1], \mathtt{a} \in \Lambda^{\rm int}_\epsilon, i \in [1, t_{\rm X}], {\rm X} \in \{\rm C, T, F\}$: $\alpha^{\rm X}(i) = [\mathtt{a}]^{\rm int} \geq 1$ (resp., $\alpha^{\rm X}(i) = 0$) $\Leftrightarrow$ $\delta^{\rm X}_\alpha(i, [\mathtt{a}]^{\rm int}) = 1$ (resp., $\delta^{\rm X}_\alpha(i, 0) = 0$) $\Leftrightarrow$ $\alpha({v^{\rm X}}_i) = \mathtt{a} \in \Lambda$ (resp., vertex ${v^{\rm X}}_i$ is not used in G);
- $\delta^{\rm X}_\alpha(i, [\mathtt{a}]^{\rm int}) \in [0, 1], i \in [1, t_{\rm X}], \mathtt{a} \in \Lambda^{\rm int}, {\rm X} \in \{\rm C, T, F\}$: $\delta^{\rm X}_\alpha(i, [\mathtt{a}]^{\rm t}) = 1 \Leftrightarrow \alpha({v^{\rm X}}_i) = \mathtt{a}$;
- $\mathrm{Mass} \in \mathbb{Z}_+$: $\sum_{v \in V(H)} \mathrm{mass}^*(\alpha(v))$;
- $\mathrm{na}([\mathtt{a}]) \in [\mathrm{na_{LB}}(\mathtt{a}), \mathrm{na_{UB}}(\mathtt{a})]$, $\mathtt{a} \in \Lambda$: the number of vertices $v \in V(H)$ with $\alpha(v) = \mathtt{a}$;
- $\mathrm{na^{int}}([\mathtt{a}]^{\rm int}) \in [\mathrm{na^{int}_{LB}}(\mathtt{a}), \mathrm{na^{int}_{UB}}(\mathtt{a})]$, $\mathtt{a} \in \Lambda, {\rm X} \in \{\rm C, T, F\}$: the number of interior-vertices $v \in V(G)$ with $\alpha(v) = \mathtt{a}$;
- $\mathrm{na^{ex}_X}([\mathtt{a}]^{\rm ex}), \mathrm{na^{ex}}([\mathtt{a}]^{\rm ex}) \in [0, \mathrm{na_{UB}}(\mathtt{a})]$, $\mathtt{a} \in \Lambda$, ${\rm X} \in \{\rm C, T, F\}$: the number of exterior-vertices rooted at vertices $v \in V_{\rm X}$ and the number of exterior-vertices v such that $\alpha(v) = \mathtt{a}$;
- $\delta^{\rm X}_{\rm hyd}(i, d) \in [0, 1], d \in [0, 3], {\rm X} \in \{\rm C, T, F\}$: $\delta^{\rm X}_{\rm hyd}(i, d) \Leftrightarrow \mathrm{deg_{hyd}}({v^{\rm X}}_i) = d$;
- $\mathrm{hydg}(d), d \in [0, 3]$: the number of vertices v with $\mathrm{deg_{hyd}}({v^{\rm X}}_i) = d$;

constraints:

$$\begin{aligned}\beta^+(k) - 3(e^{\rm T}(i) - \chi^{\rm T}(i,k) + 1) \leq \beta^{\rm CT}(i) \leq \beta^+(k) + 3(e^{\rm T}(i) - \chi^{\rm T}(i,k) + 1), i \in [1, t_{\rm T}],\\ \beta^-(k) - 3(e^{\rm T}(i+1) - \chi^{\rm T}(i,k) + 1) \leq \beta^{\rm TC}(i) \leq \beta^-(k) + 3(e^{\rm T}(i+1) - \chi^{\rm T}(i,k) + 1), i \in [1, t_{\rm T}],\\ k \in [1, k_{\rm C}],\end{aligned} \tag{43}$$

$$\begin{aligned}\beta^{\rm in}(c) - 3(e^{\rm F}(i) - \chi^{\rm F}(i,c) + 1) \leq \beta^{\rm CF}(i) \leq \beta^{\rm in}(c) + 3(e^{\rm F}(i) - \chi^{\rm F}(i,c) + 1), i \in [1, t_{\rm F}], \quad c \in [1, \widetilde{t_{\rm C}}],\\ \beta^{\rm in}(c) - 3(e^{\rm F}(i) - \chi^{\rm F}(i,c) + 1) \leq \beta^{\rm TF}(i) \leq \beta^{\rm in}(c) + 3(e^{\rm F}(i) - \chi^{\rm F}(i,c) + 1), i \in [1, t_{\rm F}], \quad c \in [\widetilde{t_{\rm C}} + 1, c_{\rm F}],\end{aligned} \tag{44}$$

$$\sum_{\mathtt{a}\in\Lambda^{\mathrm{int}}} \delta_\alpha^{\mathrm{C}}(i,[\mathtt{a}]^{\mathrm{int}}) = 1, \quad \sum_{\mathtt{a}\in\Lambda^{\mathrm{int}}} [\mathtt{a}]^{\mathrm{int}} \cdot \delta_\alpha^{\mathrm{X}}(i,[\mathtt{a}]^{\mathrm{int}}) = \alpha^{\mathrm{C}}(i), \qquad i \in [1,t_{\mathrm{C}}],$$
$$\sum_{\mathtt{a}\in\Lambda^{\mathrm{int}}} \delta_\alpha^{\mathrm{X}}(i,[\mathtt{a}]^{\mathrm{int}}) = v^{\mathrm{X}}(i), \quad \sum_{\mathtt{a}\in\Lambda^{\mathrm{int}}} [\mathtt{a}]^{\mathrm{int}} \cdot \delta_\alpha^{\mathrm{X}}(i,[\mathtt{a}]^{\mathrm{int}}) = \alpha^{\mathrm{X}}(i), \qquad i \in [1,t_{\mathrm{X}}], \mathrm{X} \in \{\mathrm{T},\mathrm{F}\}, \tag{45}$$

$$\sum_{\psi\in\mathcal{F}_i^{\mathrm{X}}\cup\mathcal{F}_\Lambda} \alpha_{\mathrm{r}}([\psi]) \cdot \delta_{\mathrm{fr}}^{\mathrm{X}}(i,[\psi]) = \alpha^{\mathrm{X}}(i), \qquad i \in [1,t_{\mathrm{X}}], \mathrm{X} \in \{\mathrm{C},\mathrm{T},\mathrm{F}\}, \tag{46}$$

$$\sum_{j\in I_{\mathrm{C}}(i)} \beta^{\mathrm{C}}(j) + \sum_{k\in I^+_{(\geq 2)}(i)\cup I^+_{(\geq 1)}(i)} \beta^+(k) + \sum_{k\in I^-_{(\geq 2)}(i)\cup I^-_{(\geq 1)}(i)} \beta^-(k)$$
$$+\beta^{\mathrm{in}}(i) + \beta^{\mathrm{C}}_{\mathrm{ex}}(i) + \sum_{d\in[0,3]} d\cdot\delta^{\mathrm{C}}_{\mathrm{hyd}}(i,d) = \sum_{\mathtt{a}\in\Lambda^{\mathrm{int}}} \mathrm{val}(\mathtt{a})\delta_\alpha^{\mathrm{C}}(i,[\mathtt{a}]^{\mathrm{int}}), \qquad i \in [1,\widetilde{t_{\mathrm{C}}}], \tag{47}$$

$$\sum_{j\in I_{\mathrm{C}}(i)} \beta^{\mathrm{C}}(j) + \sum_{k\in I^+_{(\geq 2)}(i)\cup I^+_{(\geq 1)}(i)} \beta^+(k) + \sum_{k\in I^-_{(\geq 2)}(i)\cup I^-_{(\geq 1)}(i)} \beta^-(k)$$
$$+\beta^{\mathrm{C}}_{\mathrm{ex}}(i) + \sum_{d\in[0,3]} d\cdot\delta^{\mathrm{C}}_{\mathrm{hyd}}(i,d) = \sum_{\mathtt{a}\in\Lambda^{\mathrm{int}}} \mathrm{val}(\mathtt{a})\delta_\alpha^{\mathrm{C}}(i,[\mathtt{a}]^{\mathrm{int}}), \qquad i \in [\widetilde{t_{\mathrm{C}}}+1,t_{\mathrm{C}}], \tag{48}$$

$$\beta^{\mathrm{T}}(i) + \beta^{\mathrm{T}}(i{+}1) + \beta^{\mathrm{T}}_{\mathrm{ex}}(i) + \beta^{\mathrm{CT}}(i) + \beta^{\mathrm{TC}}(i)$$
$$+\beta^{\mathrm{in}}(\widetilde{t_{\mathrm{C}}}+i) + \sum_{d\in[0,3]} d\cdot\delta^{\mathrm{T}}_{\mathrm{hyd}}(i,d) = \sum_{\mathtt{a}\in\Lambda^{\mathrm{int}}} \mathrm{val}(\mathtt{a})\delta_\alpha^{\mathrm{T}}(i,[\mathtt{a}]^{\mathrm{int}}),$$
$$i \in [1,t_{\mathrm{T}}]\ (\beta^{\mathrm{T}}(1) = \beta^{\mathrm{T}}(t_{\mathrm{T}}+1) = 0), \tag{49}$$

$$\beta^{\mathrm{F}}(i) + \beta^{\mathrm{F}}(i{+}1) + \beta^{\mathrm{CF}}(i) + \beta^{\mathrm{TF}}(i)$$
$$+\beta^{\mathrm{F}}_{\mathrm{ex}}(i) + \sum_{d\in[0,3]} d\cdot\delta^{\mathrm{F}}_{\mathrm{hyd}}(i,d) = \sum_{\mathtt{a}\in\Lambda^{\mathrm{int}}} \mathrm{val}(\mathtt{a})\delta_\alpha^{\mathrm{F}}(i,[\mathtt{a}]^{\mathrm{int}}),$$
$$i \in [1,t_{\mathrm{F}}]\ (\beta^{\mathrm{F}}(1) = \beta^{\mathrm{F}}(t_{\mathrm{F}}+1) = 0), \tag{50}$$

$$\sum_{i\in[1,t_{\mathrm{X}}]} \delta_\alpha^{\mathrm{X}}(i,[\mathtt{a}]^{\mathrm{int}}) = \mathrm{na}_{\mathrm{X}}([\mathtt{a}]^{\mathrm{int}}), \qquad \mathtt{a} \in \Lambda^{\mathrm{int}}, \mathrm{X} \in \{\mathrm{C},\mathrm{T},\mathrm{F}\}, \tag{51}$$

$$\sum_{\psi\in\mathcal{F}_i^{\mathrm{X}}} \mathrm{na}^{\mathrm{ex}}_{\mathtt{a}}([\psi]) \cdot \delta_{\mathrm{fr}}^{\mathrm{X}}(i,[\psi]) = \mathrm{na}^{\mathrm{ex}}_{\mathrm{X}}([\mathtt{a}]^{\mathrm{ex}}), \qquad \mathtt{a} \in \Lambda^{\mathrm{ex}}, \mathrm{X} \in \{\mathrm{C},\mathrm{T},\mathrm{F}\}, \tag{52}$$

$$\begin{aligned}
\mathrm{na_C}([\mathtt{a}]^{\mathrm{int}}) + \mathrm{na_T}([\mathtt{a}]^{\mathrm{int}}) + \mathrm{na_F}([\mathtt{a}]^{\mathrm{int}}) &= \mathrm{na^{int}}([\mathtt{a}]^{\mathrm{int}}), && \mathtt{a} \in \Lambda^{\mathrm{int}}, \\
\sum_{\mathrm{X}\in\{\mathrm{C,T,F}\}} \mathrm{na_X^{ex}}([\mathtt{a}]^{\mathrm{ex}}) &= \mathrm{na^{ex}}([\mathtt{a}]^{\mathrm{ex}}), && \mathtt{a} \in \Lambda^{\mathrm{ex}}, \\
\mathrm{na^{int}}([\mathtt{a}]^{\mathrm{int}}) + \mathrm{na^{ex}}([\mathtt{a}]^{\mathrm{ex}}) &= \mathrm{na}([\mathtt{a}]), && \mathtt{a} \in \Lambda^{\mathrm{int}} \cap \Lambda^{\mathrm{ex}}, \\
\mathrm{na^{int}}([\mathtt{a}]^{\mathrm{int}}) &= \mathrm{na}([\mathtt{a}]), && \mathtt{a} \in \Lambda^{\mathrm{int}} \setminus \Lambda^{\mathrm{ex}}, \\
\mathrm{na^{ex}}([\mathtt{a}]^{\mathrm{ex}}) &= \mathrm{na}([\mathtt{a}]), && \mathtt{a} \in \Lambda^{\mathrm{ex}} \setminus \Lambda^{\mathrm{int}}, \qquad (53)
\end{aligned}$$

$$\sum_{\mathtt{a}\in\Lambda} \mathrm{mass}^*(\mathtt{a}) \cdot \mathrm{na}([\mathtt{a}]) = \mathrm{Mass}, \qquad (54)$$

$$\begin{aligned}
\sum_{d\in[0,3]} \delta^{\mathrm{C}}_{\mathrm{hyd}}(i,d) &= 1, i \in [1, t_{\mathrm{C}}], \\
\sum_{d\in[0,3]} \delta^{\mathrm{X}}_{\mathrm{hyd}}(i,d) &= v^{\mathrm{X}}(i), i \in [1, t_{\mathrm{X}}], \mathrm{X} \in \{\mathrm{T}, \mathrm{F}\}, \qquad (55)
\end{aligned}$$

$$\sum_{i\in[1,t_{\mathrm{X}}],\mathrm{X}\in\{\mathrm{C,T,F}\}} \delta^{\mathrm{X}}_{\mathrm{hyd}}(i,d) + \sum_{\psi\in\mathcal{F}^{\mathrm{X}}_i, i\in[1,t_{\mathrm{X}}],\mathrm{X}\in\{\mathrm{C,T,F}\}} n_{\mathtt{H}}([\psi],d) \cdot \delta^{\mathrm{X}}_{\mathrm{fr}}(i,[\psi]) = \mathrm{hydg}(d), d \in [0,3], \qquad (56)$$

$$\sum_{\mathtt{a}\in\Lambda^*(i)} \delta^{\mathrm{C}}_{\alpha}(i, [\mathtt{a}]^{\mathrm{int}}) = 1, \qquad i \in [1, t_{\mathrm{C}}]. \qquad (57)$$

3.7 Constraints for Bounds on the Number of Bonds

We include constraints for specification of lower and upper bounds $\mathrm{bd_{LB}}$ and $\mathrm{bd_{UB}}$.

constants:

- $\mathrm{bd}_{m,\mathrm{LB}}(i), \mathrm{bd}_{m,\mathrm{UB}}(i) \in [0, \mathrm{n^{int}_{UB}}]$, $i \in [1, m_{\mathrm{C}}]$, $m \in [2,3]$: lower and upper bounds on the number of edges $e \in E(P_i)$ with bond-multiplicity $\beta(e) = m$ in the pure path P_i for edge $e_i \in E_{\mathrm{C}}$;

variables :

- $\mathrm{bd_T}(k,i,m) \in [0,1]$, $k \in [1, k_{\mathrm{C}}]$, $i \in [2, t_{\mathrm{T}}]$, $m \in [2,3]$: $\mathrm{bd_T}(k,i,m) = 1 \Leftrightarrow$ the pure path P_k for edge $e_k \in E_{\mathrm{C}}$ contains edge ${e^{\mathrm{T}}}_i$ with $\beta({e^{\mathrm{T}}}_i) = m$;

constraints:

$$\mathrm{bd}_{m,\mathrm{LB}}(i) \le \delta^{\mathrm{C}}_{\beta}(i,m) \le \mathrm{bd}_{m,\mathrm{UB}}(i), i \in I_{(=1)} \cup I_{(0/1)}, m \in [2,3], \qquad (58)$$

$$\mathrm{bd_T}(k,i,m) \ge \delta^{\mathrm{T}}_{\beta}(i,m) + \chi^{\mathrm{T}}(i,k) - 1, \quad k \in [1, k_{\mathrm{C}}], i \in [2, t_{\mathrm{T}}], m \in [2,3], \qquad (59)$$

$$\sum_{j\in[2,t_{\rm T}]} \delta_\beta^{\rm T}(j,m) \geq \sum_{k\in[1,k_{\rm C}],i\in[2,t_{\rm T}]} \mathrm{bd}_{\rm T}(k,i,m), \quad m\in[2,3], \tag{60}$$

$$\mathrm{bd}_{m,\rm LB}(k) \leq \sum_{i\in[2,t_{\rm T}]} \mathrm{bd}_{\rm T}(k,i,m) + \delta_\beta^{+}(k,m) + \delta_\beta^{-}(k,m) \leq \mathrm{bd}_{m,\rm UB}(k),$$
$$k\in[1,k_{\rm C}], m\in[2,3]. \tag{61}$$

3.8 Descriptor for the Number of Adjacency-configurations

We call a tuple $(\mathtt{a},\mathtt{b},m)\in\Lambda\times\Lambda\times[1,3]$ an *adjacency-configuration*. The adjacency-configuration of an edge-configuration $(\mu=\mathtt{a}d,\mu'=\mathtt{b}d',m)$ is defined to be $(\mathtt{a},\mathtt{b},m)$. We include constraints to compute the frequency of each adjacency-configuration in an inferred chemical graph G.

constants:

- A set $\Gamma^{\rm int}$ of edge-configurations $\gamma=(\mu,\xi,m)$ with $\mu\leq\xi$;
- Let $\overline{\gamma}$ of an edge-configuration $\gamma=(\mu,\xi,m)$ denote the edge-configuration (ξ,μ,m);
- Let $\Gamma^{\rm int}_{<}=\{(\mu,\xi,m)\in\Gamma^{\rm int}\mid\mu<\xi\}$, $\Gamma^{\rm int}_{=}=\{(\mu,\xi,m)\in\Gamma^{\rm int}\mid\mu=\xi\}$ and $\Gamma^{\rm int}_{>}=\{\overline{\gamma}\mid\gamma\in\Gamma^{\rm int}_{<}\}$;
- Let $\Gamma^{\rm int}_{\rm ac,<}$, $\Gamma^{\rm int}_{\rm ac,=}$ and $\Gamma^{\rm int}_{\rm ac,>}$ denote the sets of the adjacency-configurations of edge-configurations in the sets $\Gamma^{\rm int}_{<}$, $\Gamma^{\rm int}_{=}$ and $\Gamma^{\rm int}_{>}$, respectively;
- Let $\overline{\nu}$ of an adjacency-configuration $\nu=(\mathtt{a},\mathtt{b},m)$ denote the adjacency-configuration $(\mathtt{b},\mathtt{a},m)$;
- Prepare a coding of the set $\Gamma^{\rm int}_{\rm ac}\cup\Gamma^{\rm int}_{\rm ac,>}$ and let $[\nu]^{\rm int}$ denote the coded integer of an element ν in $\Gamma^{\rm int}_{\rm ac}\cup\Gamma^{\rm int}_{\rm ac,>}$;
- Choose subsets $\widetilde{\Gamma}^{\rm C}_{\rm ac},\widetilde{\Gamma}^{\rm T}_{\rm ac},\widetilde{\Gamma}^{\rm CT}_{\rm ac},\widetilde{\Gamma}^{\rm TC}_{\rm ac},\widetilde{\Gamma}^{\rm F}_{\rm ac},\widetilde{\Gamma}^{\rm CF}_{\rm ac},\widetilde{\Gamma}^{\rm TF}_{\rm ac}\subseteq\Gamma^{\rm int}_{\rm ac}\cup\Gamma^{\rm int}_{\rm ac,>}$; To compute the frequency of adjacency-configurations exactly, set $\widetilde{\Gamma}^{\rm C}_{\rm ac}:=\widetilde{\Gamma}^{\rm T}_{\rm ac}:=\widetilde{\Gamma}^{\rm CT}_{\rm ac}:=\widetilde{\Gamma}^{\rm TC}_{\rm ac}:=\widetilde{\Gamma}^{\rm F}_{\rm ac}:=\widetilde{\Gamma}^{\rm CF}_{\rm ac}:=\widetilde{\Gamma}^{\rm TF}_{\rm ac}:=\Gamma^{\rm int}_{\rm ac}\cup\Gamma^{\rm int}_{\rm ac,>}$;
- $\mathrm{ac}^{\rm int}_{\rm LB}(\nu),\mathrm{ac}^{\rm int}_{\rm UB}(\nu)\in[0,2\mathrm{n}^{\rm int}_{\rm UB}],\nu=(\mathtt{a},\mathtt{b},m)\in\Gamma^{\rm int}_{\rm ac}$: lower and upper bounds on the number of interior-edges $e=uv$ with $\alpha(u)=\mathtt{a}$, $\alpha(v)=\mathtt{b}$ and $\beta(e)=m$;

variables:

- $\mathrm{ac}^{\rm int}([\nu]^{\rm int})\in[\mathrm{ac}^{\rm int}_{\rm LB}(\nu),\mathrm{ac}^{\rm int}_{\rm UB}(\nu)],\nu\in\Gamma^{\rm int}_{\rm ac}$: the number of interior-edges with adjacency-configuration ν;
- $\mathrm{ac}_{\rm C}([\nu]^{\rm int})\in[0,m_{\rm C}],\nu\in\widetilde{\Gamma}^{\rm C}_{\rm ac}$, $\mathrm{ac}_{\rm T}([\nu]^{\rm int})\in[0,t_{\rm T}],\nu\in\widetilde{\Gamma}^{\rm T}_{\rm ac}$, $\mathrm{ac}_{\rm F}([\nu]^{\rm int})\in[0,t_{\rm F}],\nu\in\widetilde{\Gamma}^{\rm F}_{\rm ac}$: the number of edges $e^{\rm C}\in E_{\rm C}$ (resp., edges $e^{\rm T}\in E_{\rm T}$ and edges $e^{\rm F}\in E_{\rm F}$) with adjacency-configuration ν;
- $\mathrm{ac}_{\rm CT}([\nu]^{\rm int})\in[0,\min\{k_{\rm C},t_{\rm T}\}],\nu\in\widetilde{\Gamma}^{\rm CT}_{\rm ac}$, $\mathrm{ac}_{\rm TC}([\nu]^{\rm int})\in[0,\min\{k_{\rm C},t_{\rm T}\}],\nu\in\widetilde{\Gamma}^{\rm CT}_{\rm ac}$, $\mathrm{ac}_{\rm CF}([\nu]^{\rm int})\in[0,\widetilde{t_{\rm C}}],\nu\in\widetilde{\Gamma}^{\rm CF}_{\rm ac}$, $\mathrm{ac}_{\rm TF}([\nu]^{\rm int})\in[0,t_{\rm T}],\nu\in\widetilde{\Gamma}^{\rm TF}_{\rm ac}$: the number of edges $e^{\rm CT}\in E_{\rm CT}$ (resp., edges $e^{\rm TC}\in E_{\rm TC}$ and edges $e^{\rm CF}\in E_{\rm CF}$ and $e^{\rm TF}\in E_{\rm TF}$) with adjacency-configuration ν;
- $\delta^{\rm C}_{\rm ac}(i,[\nu]^{\rm int})\in[0,1],i\in[\widetilde{k_{\rm C}}+1,m_{\rm C}]=I_{(\geq 1)}\cup I_{(0/1)}\cup I_{(=1)},\nu\in\widetilde{\Gamma}^{\rm C}_{\rm ac}$, $\delta^{\rm T}_{\rm ac}(i,[\nu]^{\rm int})\in[0,1],i\in[2,t_{\rm T}],\nu\in\widetilde{\Gamma}^{\rm T}_{\rm ac}$, $\delta^{\rm F}_{\rm ac}(i,[\nu]^{\rm int})\in[0,1],i\in[2,t_{\rm F}],\nu\in\widetilde{\Gamma}^{\rm F}_{\rm ac}$: $\delta^{\rm X}_{\rm ac}(i,[\nu]^{\rm int})=1\Leftrightarrow$ edge ${e^{\rm X}}_i$ has adjacency-configuration ν;

- $\delta_{\mathrm{ac}}^{\mathrm{CT}}(k,[\nu]^{\mathrm{int}}), \delta_{\mathrm{ac}}^{\mathrm{TC}}(k,[\nu]^{\mathrm{int}}) \in [0,1], k \in [1,k_{\mathrm{C}}] = I_{(\geq 2)} \cup I_{(\geq 1)}, \nu \in \widetilde{\Gamma}_{\mathrm{ac}}^{\mathrm{CT}}$: $\delta_{\mathrm{ac}}^{\mathrm{CT}}(k,[\nu]^{\mathrm{int}}) = 1$ (resp., $\delta_{\mathrm{ac}}^{\mathrm{TC}}(k,[\nu]^{\mathrm{int}}) = 1$) $\Leftrightarrow$ edge ${e^{\mathrm{CT}}}_{\mathrm{tail}(k),j}$ (resp., ${e^{\mathrm{TC}}}_{\mathrm{head}(k),j}$) for some $j \in [1,t_{\mathrm{T}}]$ has adjacency-configuration ν;
- $\delta_{\mathrm{ac}}^{\mathrm{CF}}(c,[\nu]^{\mathrm{int}}) \in [0,1], c \in [1,\widetilde{t_{\mathrm{C}}}], \nu \in \widetilde{\Gamma}_{\mathrm{ac}}^{\mathrm{CF}}$: $\delta_{\mathrm{ac}}^{\mathrm{CF}}(c,[\nu]^{\mathrm{int}}) = 1 \Leftrightarrow$ edge ${e^{\mathrm{CF}}}_{c,i}$ for some $i \in [1,t_{\mathrm{F}}]$ has adjacency-configuration ν;
- $\delta_{\mathrm{ac}}^{\mathrm{TF}}(i,[\nu]^{\mathrm{int}}) \in [0,1], i \in [1,t_{\mathrm{T}}], \nu \in \widetilde{\Gamma}_{\mathrm{ac}}^{\mathrm{TF}}$: $\delta_{\mathrm{ac}}^{\mathrm{TF}}(i,[\nu]^{\mathrm{int}}) = 1 \Leftrightarrow$ edge ${e^{\mathrm{TF}}}_{i,j}$ for some $j \in [1,t_{\mathrm{F}}]$ has adjacency-configuration ν;
- $\alpha^{\mathrm{CT}}(k), \alpha^{\mathrm{TC}}(k) \in [0,|\Lambda^{\mathrm{int}}|], k \in [1,k_{\mathrm{C}}]$: $\alpha(v)$ of the edge $({v^{\mathrm{C}}}_{\mathrm{tail}(k)}, v) \in E_{\mathrm{CT}}$ (resp., $(v, {v^{\mathrm{C}}}_{\mathrm{head}(k)}) \in E_{\mathrm{TC}}$) if any;
- $\alpha^{\mathrm{CF}}(c) \in [0,|\Lambda^{\mathrm{int}}|], c \in [1,\widetilde{t_{\mathrm{C}}}]$: $\alpha(v)$ of the edge $({v^{\mathrm{C}}}_{c}, v) \in E_{\mathrm{CF}}$ if any;
- $\alpha^{\mathrm{TF}}(i) \in [0,|\Lambda^{\mathrm{int}}|], i \in [1,t_{\mathrm{T}}]$: $\alpha(v)$ of the edge $({v^{\mathrm{T}}}_{i}, v) \in E_{\mathrm{TF}}$ if any;
- $\Delta_{\mathrm{ac}}^{\mathrm{C}+}(i), \Delta_{\mathrm{ac}}^{\mathrm{C}-}(i), \in [0,|\Lambda^{\mathrm{int}}|], i \in [\widetilde{k_{\mathrm{C}}+1}, m_{\mathrm{C}}]$, $\Delta_{\mathrm{ac}}^{\mathrm{T}+}(i), \Delta_{\mathrm{ac}}^{\mathrm{T}-}(i) \in [0,|\Lambda^{\mathrm{int}}|], i \in [2,t_{\mathrm{T}}]$, $\Delta_{\mathrm{ac}}^{\mathrm{F}+}(i), \Delta_{\mathrm{ac}}^{\mathrm{F}-}(i) \in [0,|\Lambda^{\mathrm{int}}|], i \in [2,t_{\mathrm{F}}]$: $\Delta_{\mathrm{ac}}^{\mathrm{X}+}(i) = \Delta_{\mathrm{ac}}^{\mathrm{X}-}(i) = 0$ (resp., $\Delta_{\mathrm{ac}}^{\mathrm{X}+}(i) = \alpha(u)$ and $\Delta_{\mathrm{ac}}^{\mathrm{X}-}(i) = \alpha(v)$) $\Leftrightarrow$ edge ${e^{\mathrm{X}}}_{i} = (u,v) \in E_{\mathrm{X}}$ is used in G (resp., ${e^{\mathrm{X}}}_{i} \notin E(G)$);
- $\Delta_{\mathrm{ac}}^{\mathrm{CT}+}(k), \Delta_{\mathrm{ac}}^{\mathrm{CT}-}(k) \in [0,|\Lambda^{\mathrm{int}}|], k \in [1,k_{\mathrm{C}}] = I_{(\geq 2)} \cup I_{(\geq 1)}$: $\Delta_{\mathrm{ac}}^{\mathrm{CT}+}(k) = \Delta_{\mathrm{ac}}^{\mathrm{CT}-}(k) = 0$ (resp., $\Delta_{\mathrm{ac}}^{\mathrm{CT}+}(k) = \alpha(u)$ and $\Delta_{\mathrm{ac}}^{\mathrm{CT}-}(k) = \alpha(v)$) $\Leftrightarrow$ edge ${e^{\mathrm{CT}}}_{\mathrm{tail}(k),j} = (u,v) \in E_{\mathrm{CT}}$ for some $j \in [1,t_{\mathrm{T}}]$ is used in G (resp., otherwise);
- $\Delta_{\mathrm{ac}}^{\mathrm{TC}+}(k), \Delta_{\mathrm{ac}}^{\mathrm{TC}-}(k) \in [0,|\Lambda^{\mathrm{int}}|], k \in [1,k_{\mathrm{C}}] = I_{(\geq 2)} \cup I_{(\geq 1)}$: Analogous with $\Delta_{\mathrm{ac}}^{\mathrm{CT}+}(k)$ and $\Delta_{\mathrm{ac}}^{\mathrm{CT}-}(k)$;
- $\Delta_{\mathrm{ac}}^{\mathrm{CF}+}(c) \in [0,|\Lambda^{\mathrm{int}}|], \Delta_{\mathrm{ac}}^{\mathrm{CF}-}(c) \in [0,|\Lambda^{\mathrm{int}}|], c \in [1,\widetilde{t_{\mathrm{C}}}]$: $\Delta_{\mathrm{ac}}^{\mathrm{CF}+}(c) = \Delta_{\mathrm{ac}}^{\mathrm{CF}-}(c) = 0$ (resp., $\Delta_{\mathrm{ac}}^{\mathrm{CF}+}(c) = \alpha(u)$ and $\Delta_{\mathrm{ac}}^{\mathrm{CF}-}(c) = \alpha(v)$) $\Leftrightarrow$ edge ${e^{\mathrm{CF}}}_{c,i} = (u,v) \in E_{\mathrm{CF}}$ for some $i \in [1,t_{\mathrm{F}}]$ is used in G (resp., otherwise);
- $\Delta_{\mathrm{ac}}^{\mathrm{TF}+}(i) \in [0,|\Lambda^{\mathrm{int}}|], \Delta_{\mathrm{ac}}^{\mathrm{TF}-}(i) \in [0,|\Lambda^{\mathrm{int}}|], i \in [1,t_{\mathrm{T}}]$: Analogous with $\Delta_{\mathrm{ac}}^{\mathrm{CF}+}(c)$ and $\Delta_{\mathrm{ac}}^{\mathrm{CF}-}(c)$;

constraints:

$$
\begin{aligned}
\mathrm{ac}_{\mathrm{C}}([\nu]^{\mathrm{int}}) &= 0, & \nu &\in \Gamma_{\mathrm{ac}}^{\mathrm{int}} \setminus \widetilde{\Gamma}_{\mathrm{ac}}^{\mathrm{C}}, \\
\mathrm{ac}_{\mathrm{T}}([\nu]^{\mathrm{int}}) &= 0, & \nu &\in \Gamma_{\mathrm{ac}}^{\mathrm{int}} \setminus \widetilde{\Gamma}_{\mathrm{ac}}^{\mathrm{T}}, \\
\mathrm{ac}_{\mathrm{F}}([\nu]^{\mathrm{int}}) &= 0, & \nu &\in \Gamma_{\mathrm{ac}}^{\mathrm{int}} \setminus \widetilde{\Gamma}_{\mathrm{ac}}^{\mathrm{F}}, \\
\mathrm{ac}_{\mathrm{CT}}([\nu]^{\mathrm{int}}) &= 0, & \nu &\in \Gamma_{\mathrm{ac}}^{\mathrm{int}} \setminus \widetilde{\Gamma}_{\mathrm{ac}}^{\mathrm{CT}}, \\
\mathrm{ac}_{\mathrm{TC}}([\nu]^{\mathrm{int}}) &= 0, & \nu &\in \Gamma_{\mathrm{ac}}^{\mathrm{int}} \setminus \widetilde{\Gamma}_{\mathrm{ac}}^{\mathrm{TC}}, \\
\mathrm{ac}_{\mathrm{CF}}([\nu]^{\mathrm{int}}) &= 0, & \nu &\in \Gamma_{\mathrm{ac}}^{\mathrm{int}} \setminus \widetilde{\Gamma}_{\mathrm{ac}}^{\mathrm{CF}}, \\
\mathrm{ac}_{\mathrm{TF}}([\nu]^{\mathrm{int}}) &= 0, & \nu &\in \Gamma_{\mathrm{ac}}^{\mathrm{int}} \setminus \widetilde{\Gamma}_{\mathrm{ac}}^{\mathrm{TF}},
\end{aligned}
\tag{62}
$$

$$\begin{aligned}
\sum_{(\mathsf{a},\mathsf{b},m)=\nu\in\Gamma^{\rm int}_{\rm ac}} \mathrm{ac}_{\rm C}([\nu]^{\rm int}) &= \sum_{i\in[\widetilde{k_{\rm C}}+1,m_{\rm C}]} \delta^{\rm C}_{\beta}(i,m), && m\in[1,3],\\
\sum_{(\mathsf{a},\mathsf{b},m)=\nu\in\Gamma^{\rm int}_{\rm ac}} \mathrm{ac}_{\rm T}([\nu]^{\rm int}) &= \sum_{i\in[2,t_{\rm T}]} \delta^{\rm T}_{\beta}(i,m), && m\in[1,3],\\
\sum_{(\mathsf{a},\mathsf{b},m)=\nu\in\Gamma^{\rm int}_{\rm ac}} \mathrm{ac}_{\rm F}([\nu]^{\rm int}) &= \sum_{i\in[2,t_{\rm F}]} \delta^{\rm F}_{\beta}(i,m), && m\in[1,3],\\
\sum_{(\mathsf{a},\mathsf{b},m)=\nu\in\Gamma^{\rm int}_{\rm ac}} \mathrm{ac}_{\rm CT}([\nu]^{\rm int}) &= \sum_{k\in[1,k_{\rm C}]} \delta^{+}_{\beta}(k,m), && m\in[1,3],\\
\sum_{(\mathsf{a},\mathsf{b},m)=\nu\in\Gamma^{\rm int}_{\rm ac}} \mathrm{ac}_{\rm TC}([\nu]^{\rm int}) &= \sum_{k\in[1,k_{\rm C}]} \delta^{-}_{\beta}(k,m), && m\in[1,3],\\
\sum_{(\mathsf{a},\mathsf{b},m)=\nu\in\Gamma^{\rm int}_{\rm ac}} \mathrm{ac}_{\rm CF}([\nu]^{\rm int}) &= \sum_{c\in[1,\widetilde{t_{\rm C}}]} \delta^{\rm in}_{\beta}(c,m), && m\in[1,3],\\
\sum_{(\mathsf{a},\mathsf{b},m)=\nu\in\Gamma^{\rm int}_{\rm ac}} \mathrm{ac}_{\rm TF}([\nu]^{\rm int}) &= \sum_{c\in[\widetilde{t_{\rm C}}+1,c_{\rm F}]} \delta^{\rm in}_{\beta}(c,m), && m\in[1,3],
\end{aligned} \tag{63}$$

$$\begin{aligned}
\sum_{\nu=(\mathsf{a},\mathsf{b},m)\in\widetilde{\Gamma}^{\rm C}_{\rm ac}} m\cdot\delta^{\rm C}_{\rm ac}(i,[\nu]^{\rm int}) &= \beta^{\rm C}(i),\\
\Delta^{\rm C+}_{\rm ac}(i)+\sum_{\nu=(\mathsf{a},\mathsf{b},m)\in\widetilde{\Gamma}^{\rm C}_{\rm ac}} [\mathsf{a}]^{\rm int}\delta^{\rm C}_{\rm ac}(i,[\nu]^{\rm int}) &= \alpha^{\rm C}(\mathrm{tail}(i)),\\
\Delta^{\rm C-}_{\rm ac}(i)+\sum_{\nu=(\mathsf{a},\mathsf{b},m)\in\widetilde{\Gamma}^{\rm C}_{\rm ac}} [\mathsf{b}]^{\rm int}\delta^{\rm C}_{\rm ac}(i,[\nu]^{\rm int}) &= \alpha^{\rm C}(\mathrm{head}(i)),\\
\Delta^{\rm C+}_{\rm ac}(i)+\Delta^{\rm C-}_{\rm ac}(i) &\le 2|\Lambda^{\rm int}|(1-e^{\rm C}(i)), && i\in[\widetilde{k_{\rm C}}+1,m_{\rm C}],\\
\sum_{i\in[\widetilde{k_{\rm C}}+1,m_{\rm C}]} \delta^{\rm C}_{\rm ac}(i,[\nu]^{\rm int}) &= \mathrm{ac}_{\rm C}([\nu]^{\rm int}), && \nu\in\widetilde{\Gamma}^{\rm C}_{\rm ac},
\end{aligned} \tag{64}$$

$$\begin{aligned}
\sum_{\nu=(\mathsf{a},\mathsf{b},m)\in\widetilde{\Gamma}^{\rm T}_{\rm ac}} m\cdot\delta^{\rm T}_{\rm ac}(i,[\nu]^{\rm int}) &= \beta^{\rm T}(i),\\
\Delta^{\rm T+}_{\rm ac}(i)+\sum_{\nu=(\mathsf{a},\mathsf{b},m)\in\widetilde{\Gamma}^{\rm T}_{\rm ac}} [\mathsf{a}]^{\rm int}\delta^{\rm T}_{\rm ac}(i,[\nu]^{\rm int}) &= \alpha^{\rm T}(i-1),\\
\Delta^{\rm T-}_{\rm ac}(i)+\sum_{\nu=(\mathsf{a},\mathsf{b},m)\in\widetilde{\Gamma}^{\rm T}_{\rm ac}} [\mathsf{b}]^{\rm int}\delta^{\rm T}_{\rm ac}(i,[\nu]^{\rm int}) &= \alpha^{\rm T}(i),\\
\Delta^{\rm T+}_{\rm ac}(i)+\Delta^{\rm T-}_{\rm ac}(i) &\le 2|\Lambda^{\rm int}|(1-e^{\rm T}(i)), && i\in[2,t_{\rm T}],\\
\sum_{i\in[2,t_{\rm T}]} \delta^{\rm T}_{\rm ac}(i,[\nu]^{\rm int}) &= \mathrm{ac}_{\rm T}([\nu]^{\rm int}), && \nu\in\widetilde{\Gamma}^{\rm T}_{\rm ac},
\end{aligned} \tag{65}$$

$$\begin{aligned}
&\sum_{\nu=(\mathtt{a},\mathtt{b},m)\in\widetilde{\Gamma}^{\mathrm{F}}_{\mathrm{ac}}} m\cdot\delta^{\mathrm{F}}_{\mathrm{ac}}(i,[\nu]^{\mathrm{int}})=\beta^{\mathrm{F}}(i),\\
&\Delta^{\mathrm{F}+}_{\mathrm{ac}}(i)+\sum_{\nu=(\mathtt{a},\mathtt{b},m)\in\widetilde{\Gamma}^{\mathrm{F}}_{\mathrm{ac}}}[\mathtt{a}]^{\mathrm{int}}\delta^{\mathrm{F}}_{\mathrm{ac}}(i,[\nu]^{\mathrm{int}})=\alpha^{\mathrm{F}}(i-1),\\
&\Delta^{\mathrm{F}-}_{\mathrm{ac}}(i)+\sum_{\nu=(\mathtt{a},\mathtt{b},m)\in\widetilde{\Gamma}^{\mathrm{F}}_{\mathrm{ac}}}[\mathtt{b}]^{\mathrm{int}}\delta^{\mathrm{F}}_{\mathrm{ac}}(i,[\nu]^{\mathrm{int}})=\alpha^{\mathrm{F}}(i),\\
&\Delta^{\mathrm{F}+}_{\mathrm{ac}}(i)+\Delta^{\mathrm{F}-}_{\mathrm{ac}}(i)\le 2|\Lambda^{\mathrm{ex}}|(1-e^{\mathrm{F}}(i)), && i\in[2,t_{\mathrm{F}}],\\
&\sum_{i\in[2,t_{\mathrm{F}}]}\delta^{\mathrm{F}}_{\mathrm{ac}}(i,[\nu]^{\mathrm{int}})=\mathrm{ac}_{\mathrm{F}}([\nu]^{\mathrm{int}}), && \nu\in\widetilde{\Gamma}^{\mathrm{F}}_{\mathrm{ac}},
\end{aligned}\tag{66}$$

$$\begin{aligned}
&\alpha^{\mathrm{T}}(i)+|\Lambda^{\mathrm{int}}|(1-\chi^{\mathrm{T}}(i,k)+e^{\mathrm{T}}(i))\ge\alpha^{\mathrm{CT}}(k),\\
&\alpha^{\mathrm{CT}}(k)\ge\alpha^{\mathrm{T}}(i)-|\Lambda^{\mathrm{int}}|(1-\chi^{\mathrm{T}}(i,k)+e^{\mathrm{T}}(i)), && i\in[1,t_{\mathrm{T}}],\\
&\sum_{\nu=(\mathtt{a},\mathtt{b},m)\in\widetilde{\Gamma}^{\mathrm{CT}}_{\mathrm{ac}}} m\cdot\delta^{\mathrm{CT}}_{\mathrm{ac}}(k,[\nu]^{\mathrm{int}})=\beta^{+}(k),\\
&\Delta^{\mathrm{CT}+}_{\mathrm{ac}}(k)+\sum_{\nu=(\mathtt{a},\mathtt{b},m)\in\widetilde{\Gamma}^{\mathrm{CT}}_{\mathrm{ac}}}[\mathtt{a}]^{\mathrm{int}}\delta^{\mathrm{CT}}_{\mathrm{ac}}(k,[\nu]^{\mathrm{int}})=\alpha^{\mathrm{C}}(\mathrm{tail}(k)),\\
&\Delta^{\mathrm{CT}-}_{\mathrm{ac}}(k)+\sum_{\nu=(\mathtt{a},\mathtt{b},m)\in\widetilde{\Gamma}^{\mathrm{CT}}_{\mathrm{ac}}}[\mathtt{b}]^{\mathrm{int}}\delta^{\mathrm{CT}}_{\mathrm{ac}}(k,[\nu]^{\mathrm{int}})=\alpha^{\mathrm{CT}}(k),\\
&\Delta^{\mathrm{CT}+}_{\mathrm{ac}}(k)+\Delta^{\mathrm{CT}-}_{\mathrm{ac}}(k)\le 2|\Lambda^{\mathrm{int}}|(1-\delta^{\mathrm{T}}_{\chi}(k)), && k\in[1,k_{\mathrm{C}}],\\
&\sum_{k\in[1,k_{\mathrm{C}}]}\delta^{\mathrm{CT}}_{\mathrm{ac}}(k,[\nu]^{\mathrm{int}})=\mathrm{ac}_{\mathrm{CT}}([\nu]^{\mathrm{int}}), && \nu\in\widetilde{\Gamma}^{\mathrm{CT}}_{\mathrm{ac}},
\end{aligned}\tag{67}$$

$$\begin{aligned}
&\alpha^{\mathrm{T}}(i)+|\Lambda^{\mathrm{int}}|(1-\chi^{\mathrm{T}}(i,k)+e^{\mathrm{T}}(i+1))\ge\alpha^{\mathrm{TC}}(k),\\
&\alpha^{\mathrm{TC}}(k)\ge\alpha^{\mathrm{T}}(i)-|\Lambda^{\mathrm{int}}|(1-\chi^{\mathrm{T}}(i,k)+e^{\mathrm{T}}(i+1)), && i\in[1,t_{\mathrm{T}}],\\
&\sum_{\nu=(\mathtt{a},\mathtt{b},m)\in\widetilde{\Gamma}^{\mathrm{TC}}_{\mathrm{ac}}} m\cdot\delta^{\mathrm{TC}}_{\mathrm{ac}}(k,[\nu]^{\mathrm{int}})=\beta^{-}(k),\\
&\Delta^{\mathrm{TC}+}_{\mathrm{ac}}(k)+\sum_{\nu=(\mathtt{a},\mathtt{b},m)\in\widetilde{\Gamma}^{\mathrm{TC}}_{\mathrm{ac}}}[\mathtt{a}]^{\mathrm{int}}\delta^{\mathrm{TC}}_{\mathrm{ac}}(k,[\nu]^{\mathrm{int}})=\alpha^{\mathrm{TC}}(k),\\
&\Delta^{\mathrm{TC}-}_{\mathrm{ac}}(k)+\sum_{\nu=(\mathtt{a},\mathtt{b},m)\in\widetilde{\Gamma}^{\mathrm{TC}}_{\mathrm{ac}}}[\mathtt{b}]^{\mathrm{int}}\delta^{\mathrm{TC}}_{\mathrm{ac}}(k,[\nu]^{\mathrm{int}})=\alpha^{\mathrm{C}}(\mathrm{head}(k)),\\
&\Delta^{\mathrm{TC}+}_{\mathrm{ac}}(k)+\Delta^{\mathrm{TC}-}_{\mathrm{ac}}(k)\le 2|\Lambda^{\mathrm{int}}|(1-\delta^{\mathrm{T}}_{\chi}(k)), && k\in[1,k_{\mathrm{C}}],\\
&\sum_{k\in[1,k_{\mathrm{C}}]}\delta^{\mathrm{TC}}_{\mathrm{ac}}(k,[\nu]^{\mathrm{int}})=\mathrm{ac}_{\mathrm{TC}}([\nu]^{\mathrm{int}}), && \nu\in\widetilde{\Gamma}^{\mathrm{TC}}_{\mathrm{ac}},
\end{aligned}\tag{68}$$

$$\begin{aligned}
\alpha^{\mathrm{F}}(i) + |\Lambda^{\mathrm{int}}|(1-\chi^{\mathrm{F}}(i,c)+e^{\mathrm{F}}(i)) \geq \alpha^{\mathrm{CF}}(c),& \\
\alpha^{\mathrm{CF}}(c) \geq \alpha^{\mathrm{F}}(i) - |\Lambda^{\mathrm{int}}|(1-\chi^{\mathrm{F}}(i,c)+e^{\mathrm{F}}(i)),& \qquad i \in [1,t_{\mathrm{F}}], \\
\sum_{\nu=(\mathtt{a},\mathtt{b},m)\in\widetilde{\Gamma}^{\mathrm{CF}}_{\mathrm{ac}}} m\cdot\delta^{\mathrm{CF}}_{\mathrm{ac}}(c,[\nu]^{\mathrm{int}}) = \beta^{\mathrm{in}}(c),& \\
\Delta^{\mathrm{CF}+}_{\mathrm{ac}}(c) + \sum_{\nu=(\mathtt{a},\mathtt{b},m)\in\widetilde{\Gamma}^{\mathrm{CF}}_{\mathrm{ac}}} [\mathtt{a}]^{\mathrm{int}}\delta^{\mathrm{CF}}_{\mathrm{ac}}(c,[\nu]^{\mathrm{int}}) = \alpha^{\mathrm{C}}(\mathrm{head}(c)),& \\
\Delta^{\mathrm{CF}-}_{\mathrm{ac}}(c) + \sum_{\nu=(\mathtt{a},\mathtt{b},m)\in\widetilde{\Gamma}^{\mathrm{CF}}_{\mathrm{ac}}} [\mathtt{b}]^{\mathrm{int}}\delta^{\mathrm{CF}}_{\mathrm{ac}}(c,[\nu]^{\mathrm{int}}) = \alpha^{\mathrm{CF}}(c),& \\
\Delta^{\mathrm{CF}+}_{\mathrm{ac}}(c) + \Delta^{\mathrm{CF}-}_{\mathrm{ac}}(c) \leq 2\max\{|\Lambda^{\mathrm{int}}|,|\Lambda^{\mathrm{int}}|\}(1-\delta^{\mathrm{F}}_{\chi}(c)),& \qquad c \in [1,\widetilde{t_{\mathrm{C}}}], \\
\sum_{c\in[1,\widetilde{t_{\mathrm{C}}}]} \delta^{\mathrm{CF}}_{\mathrm{ac}}(c,[\nu]^{\mathrm{int}}) = \mathrm{ac}_{\mathrm{CF}}([\nu]^{\mathrm{int}}),& \qquad \nu\in\widetilde{\Gamma}^{\mathrm{CF}}_{\mathrm{ac}},
\end{aligned} \tag{69}$$

$$\begin{aligned}
\alpha^{\mathrm{F}}(j) + |\Lambda^{\mathrm{int}}|(1-\chi^{\mathrm{F}}(j,i+\widetilde{t_{\mathrm{C}}})+e^{\mathrm{F}}(j)) \geq \alpha^{\mathrm{TF}}(i),& \\
\alpha^{\mathrm{TF}}(i) \geq \alpha^{\mathrm{F}}(j) - |\Lambda^{\mathrm{int}}|(1-\chi^{\mathrm{F}}(j,i+\widetilde{t_{\mathrm{C}}})+e^{\mathrm{F}}(j)),& \qquad j \in [1,t_{\mathrm{F}}], \\
\sum_{\nu=(\mathtt{a},\mathtt{b},m)\in\widetilde{\Gamma}^{\mathrm{TF}}_{\mathrm{ac}}} m\cdot\delta^{\mathrm{TF}}_{\mathrm{ac}}(i,[\nu]^{\mathrm{int}}) = \beta^{\mathrm{in}}(i+\widetilde{t_{\mathrm{C}}}),& \\
\Delta^{\mathrm{TF}+}_{\mathrm{ac}}(i) + \sum_{\nu=(\mathtt{a},\mathtt{b},m)\in\widetilde{\Gamma}^{\mathrm{TF}}_{\mathrm{ac}}} [\mathtt{a}]^{\mathrm{int}}\delta^{\mathrm{TF}}_{\mathrm{ac}}(i,[\nu]^{\mathrm{int}}) = \alpha^{\mathrm{T}}(i),& \\
\Delta^{\mathrm{TF}-}_{\mathrm{ac}}(i) + \sum_{\nu=(\mathtt{a},\mathtt{b},m)\in\widetilde{\Gamma}^{\mathrm{TF}}_{\mathrm{ac}}} [\mathtt{b}]^{\mathrm{int}}\delta^{\mathrm{TF}}_{\mathrm{ac}}(i,[\nu]^{\mathrm{int}}) = \alpha^{\mathrm{TF}}(i),& \\
\Delta^{\mathrm{TF}+}_{\mathrm{ac}}(i) + \Delta^{\mathrm{TF}-}_{\mathrm{ac}}(i) \leq 2\max\{|\Lambda^{\mathrm{int}}|,|\Lambda^{\mathrm{int}}|\}(1-\delta^{\mathrm{F}}_{\chi}(i+\widetilde{t_{\mathrm{C}}})),& \qquad i \in [1,t_{\mathrm{T}}], \\
\sum_{i\in[1,t_{\mathrm{T}}]} \delta^{\mathrm{TF}}_{\mathrm{ac}}(i,[\nu]^{\mathrm{int}}) = \mathrm{ac}_{\mathrm{TF}}([\nu]^{\mathrm{int}}),& \qquad \nu\in\widetilde{\Gamma}^{\mathrm{TF}}_{\mathrm{ac}},
\end{aligned} \tag{70}$$

$$\begin{aligned}
\sum_{\mathrm{X}\in\{\mathrm{C,T,F,CT,TC,CF,TF}\}} (\mathrm{ac}_{\mathrm{X}}([\nu]^{\mathrm{int}}) + \mathrm{ac}_{\mathrm{X}}([\overline{\nu}]^{\mathrm{int}})) = \mathrm{ac}^{\mathrm{int}}([\nu]^{\mathrm{int}}),& \qquad \nu\in\Gamma^{\mathrm{int}}_{\mathrm{ac},<}, \\
\sum_{\mathrm{X}\in\{\mathrm{C,T,F,CT,TC,CF,TF}\}} \mathrm{ac}_{\mathrm{X}}([\nu]^{\mathrm{int}}) = \mathrm{ac}^{\mathrm{int}}([\nu]^{\mathrm{int}}),& \qquad \nu\in\Gamma^{\mathrm{int}}_{\mathrm{ac},=}.
\end{aligned} \tag{71}$$

3.9 Descriptor for the Number of Chemical Symbols

We include constraints for computing the frequency of each chemical symbol in Λ_{dg}. Let $\mathrm{cs}(v)$ denote the chemical symbol of a vertex v in a chemical graph G to be inferred; i.e., $\mathrm{cs}(v) = \mu = \mathtt{a}d \in \Lambda_{\mathrm{dg}}$ such that $\alpha(v) = \mathtt{a}$ and $\deg_G(v) = d$.

constants:

- A set $\Lambda^{\mathrm{int}}_{\mathrm{dg}}$ of chemical symbols;
- Prepare a coding of each of the two sets $\Lambda^{\mathrm{int}}_{\mathrm{dg}}$ and let $[\mu]^{\mathrm{int}}$ denote the coded integer of an element $\mu \in \Lambda^{\mathrm{int}}_{\mathrm{dg}}$;

- Choose subsets $\widetilde{\Lambda}^{\mathrm{C}}_{\mathrm{dg}}, \widetilde{\Lambda}^{\mathrm{T}}_{\mathrm{dg}}, \widetilde{\Lambda}^{\mathrm{F}}_{\mathrm{dg}} \subseteq \Lambda^{\mathrm{int}}_{\mathrm{dg}}$: To compute the frequency of chemical symbols exactly, set $\widetilde{\Lambda}^{\mathrm{C}}_{\mathrm{dg}} := \widetilde{\Lambda}^{\mathrm{T}}_{\mathrm{dg}} := \widetilde{\Lambda}^{\mathrm{F}}_{\mathrm{dg}} := \Lambda^{\mathrm{int}}_{\mathrm{dg}}$;

variables:

- $\mathrm{ns}^{\mathrm{int}}([\mu]^{\mathrm{int}}) \in [0, \mathrm{n}^{\mathrm{int}}_{\mathrm{UB}}]$, $\mu \in \Lambda^{\mathrm{int}}_{\mathrm{dg}}$: the number of interior-vertices v with $\mathrm{cs}(v) = \mu$;
- $\delta^{\mathrm{X}}_{\mathrm{ns}}(i, [\mu]^{\mathrm{int}}) \in [0, 1]$, $i \in [1, t_{\mathrm{X}}], \mu \in \Lambda^{\mathrm{int}}_{\mathrm{dg}}$, $\mathrm{X} \in \{\mathrm{C}, \mathrm{T}, \mathrm{F}\}$;

constraints:

$$\sum_{\mu \in \widetilde{\Lambda}^{\mathrm{X}}_{\mathrm{dg}} \cup \{\epsilon\}} \delta^{\mathrm{X}}_{\mathrm{ns}}(i, [\mu]^{\mathrm{int}}) = 1, \qquad \sum_{\mu = \mathtt{a}d \in \widetilde{\Lambda}^{\mathrm{X}}_{\mathrm{dg}}} [\mathtt{a}]^{\mathrm{int}} \cdot \delta^{\mathrm{X}}_{\mathrm{ns}}(i, [\mu]^{\mathrm{int}}) = \alpha^{\mathrm{X}}(i),$$
$$\sum_{\mu = \mathtt{a}d \in \widetilde{\Lambda}^{\mathrm{X}}_{\mathrm{dg}}} d \cdot \delta^{\mathrm{X}}_{\mathrm{ns}}(i, [\mu]^{\mathrm{int}}) = \deg^{\mathrm{X}}(i),$$
$$i \in [1, t_{\mathrm{X}}], \mathrm{X} \in \{\mathrm{C}, \mathrm{T}, \mathrm{F}\}, \tag{72}$$

$$\sum_{i \in [1, t_{\mathrm{C}}]} \delta^{\mathrm{C}}_{\mathrm{ns}}(i, [\mu]^{\mathrm{int}}) + \sum_{i \in [1, t_{\mathrm{T}}]} \delta^{\mathrm{T}}_{\mathrm{ns}}(i, [\mu]^{\mathrm{int}}) + \sum_{i \in [1, t_{\mathrm{F}}]} \delta^{\mathrm{F}}_{\mathrm{ns}}(i, [\mu]^{\mathrm{int}}) = \mathrm{ns}^{\mathrm{int}}([\mu]^{\mathrm{int}}), \qquad \mu \in \Lambda^{\mathrm{int}}_{\mathrm{dg}}. \tag{73}$$

3.10 Descriptor for the Number of Edge-configurations

We include constraints to compute the frequency of each edge-configuration in an inferred chemical graph G.

constants:

- A set Γ^{int} of edge-configurations $\gamma = (\mu, \xi, m)$ with $\mu \leq \xi$;
- Let $\Gamma^{\mathrm{int}}_{<} = \{(\mu, \xi, m) \in \Gamma^{\mathrm{int}} \mid \mu < \xi\}$, $\Gamma^{\mathrm{int}}_{=} = \{(\mu, \xi, m) \in \Gamma^{\mathrm{int}} \mid \mu = \xi\}$ and $\Gamma^{\mathrm{int}}_{>} = \{(\xi, \mu, m) \mid (\mu, \xi, m) \in \Gamma^{\mathrm{int}}_{<}\}$;
- Prepare a coding of the set $\Gamma^{\mathrm{int}} \cup \Gamma^{\mathrm{int}}_{>}$ and let $[\gamma]^{\mathrm{int}}$ denote the coded integer of an element γ in $\Gamma^{\mathrm{int}} \cup \Gamma^{\mathrm{int}}_{>}$;
- Choose subsets $\widetilde{\Gamma}^{\mathrm{C}}_{\mathrm{ec}}, \widetilde{\Gamma}^{\mathrm{T}}_{\mathrm{ec}}, \widetilde{\Gamma}^{\mathrm{CT}}_{\mathrm{ec}}, \widetilde{\Gamma}^{\mathrm{TC}}_{\mathrm{ec}}, \widetilde{\Gamma}^{\mathrm{F}}_{\mathrm{ec}}, \widetilde{\Gamma}^{\mathrm{CF}}_{\mathrm{ec}}, \widetilde{\Gamma}^{\mathrm{TF}}_{\mathrm{ec}} \subseteq \Gamma^{\mathrm{int}} \cup \Gamma^{\mathrm{int}}_{>}$; To compute the frequency of edge-configurations exactly, set $\widetilde{\Gamma}^{\mathrm{C}}_{\mathrm{ec}} := \widetilde{\Gamma}^{\mathrm{T}}_{\mathrm{ec}} := \widetilde{\Gamma}^{\mathrm{CT}}_{\mathrm{ec}} := \widetilde{\Gamma}^{\mathrm{TC}}_{\mathrm{ec}} := \widetilde{\Gamma}^{\mathrm{F}}_{\mathrm{ec}} := \widetilde{\Gamma}^{\mathrm{CF}}_{\mathrm{ec}} := \widetilde{\Gamma}^{\mathrm{TF}}_{\mathrm{ec}} := \Gamma^{\mathrm{int}} \cup \Gamma^{\mathrm{int}}_{>}$;
- $\mathrm{ec}^{\mathrm{int}}_{\mathrm{LB}}(\gamma), \mathrm{ec}^{\mathrm{int}}_{\mathrm{UB}}(\gamma) \in [0, 2\mathrm{n}^{\mathrm{int}}_{\mathrm{UB}}], \gamma = (\mu, \xi, m) \in \Gamma^{\mathrm{int}}$: lower and upper bounds on the number of interior-edges $e = uv$ with $\mathrm{cs}(u) = \mu$, $\mathrm{cs}(v) = \xi$ and $\beta(e) = m$;

variables:

- $\mathrm{ec}^{\mathrm{int}}([\gamma]^{\mathrm{int}}) \in [\mathrm{ec}^{\mathrm{int}}_{\mathrm{LB}}(\gamma), \mathrm{ec}^{\mathrm{int}}_{\mathrm{UB}}(\gamma)], \gamma \in \Gamma^{\mathrm{int}}$: the number of interior-edges with edge-configuration γ;
- $\mathrm{ec}_{\mathrm{C}}([\gamma]^{\mathrm{int}}) \in [0, m_{\mathrm{C}}], \gamma \in \widetilde{\Gamma}^{\mathrm{C}}_{\mathrm{ec}}$, $\mathrm{ec}_{\mathrm{T}}([\gamma]^{\mathrm{int}}) \in [0, t_{\mathrm{T}}], \gamma \in \widetilde{\Gamma}^{\mathrm{T}}_{\mathrm{ec}}$, $\mathrm{ec}_{\mathrm{F}}([\gamma]^{\mathrm{int}}) \in [0, t_{\mathrm{F}}], \gamma \in \widetilde{\Gamma}^{\mathrm{F}}_{\mathrm{ec}}$: the number of edges $e^{\mathrm{C}} \in E_{\mathrm{C}}$ (resp., edges $e^{\mathrm{T}} \in E_{\mathrm{T}}$ and edges $e^{\mathrm{F}} \in E_{\mathrm{F}}$) with edge-configuration γ;
- $\mathrm{ec}_{\mathrm{CT}}([\gamma]^{\mathrm{int}}) \in [0, \min\{k_{\mathrm{C}}, t_{\mathrm{T}}\}], \gamma \in \widetilde{\Gamma}^{\mathrm{CT}}_{\mathrm{ec}}$, $\mathrm{ec}_{\mathrm{TC}}([\gamma]^{\mathrm{int}}) \in [0, \min\{k_{\mathrm{C}}, t_{\mathrm{T}}\}], \gamma \in \widetilde{\Gamma}^{\mathrm{CT}}_{\mathrm{ec}}$, $\mathrm{ec}_{\mathrm{CF}}([\gamma]^{\mathrm{int}}) \in [0, \widetilde{t_{\mathrm{C}}}], \gamma \in \widetilde{\Gamma}^{\mathrm{CF}}_{\mathrm{ec}}$, $\mathrm{ec}_{\mathrm{TF}}([\gamma]^{\mathrm{int}}) \in [0, t_{\mathrm{T}}], \gamma \in \widetilde{\Gamma}^{\mathrm{TF}}_{\mathrm{ec}}$: the number of edges $e^{\mathrm{CT}} \in E_{\mathrm{CT}}$ (resp., edges $e^{\mathrm{TC}} \in E_{\mathrm{TC}}$ and edges $e^{\mathrm{CF}} \in E_{\mathrm{CF}}$ and $e^{\mathrm{TF}} \in E_{\mathrm{TF}}$) with edge-configuration γ;

- $\delta_{\rm ec}^{\rm C}(i,[\gamma]^{\rm int}) \in [0,1], i \in [\widetilde{k_{\rm C}}+1, m_{\rm C}] = I_{(\geq 1)} \cup I_{(0/1)} \cup I_{(=1)}, \gamma \in \widetilde{\Gamma}_{\rm ec}^{\rm C}$, $\delta_{\rm ec}^{\rm T}(i,[\gamma]^{\rm int}) \in [0,1], i \in [2,t_{\rm T}], \gamma \in \widetilde{\Gamma}_{\rm ec}^{\rm T}$, $\delta_{\rm ec}^{\rm F}(i,[\gamma]^{\rm int}) \in [0,1], i \in [2,t_{\rm F}], \gamma \in \widetilde{\Gamma}_{\rm ec}^{\rm F}$: $\delta_{\rm ec}^{\rm X}(i,[\gamma]^{\rm t}) = 1 \Leftrightarrow$ edge ${e^{\rm X}}_i$ has edge-configuration γ;
- $\delta_{\rm ec,C}^{\rm CT}(k,[\gamma]^{\rm int}), \delta_{\rm ec,C}^{\rm TC}(k,[\gamma]^{\rm int}) \in [0,1], k \in [1,k_{\rm C}] = I_{(\geq 2)} \cup I_{(\geq 1)}, \gamma \in \widetilde{\Gamma}_{\rm ec}^{\rm CT}$: $\delta_{\rm ec,C}^{\rm CT}(k,[\gamma]^{\rm int}) = 1$ (resp., $\delta_{\rm ec,C}^{\rm TC}(k,[\gamma]^{\rm int}) = 1$) $\Leftrightarrow$ edge ${e^{\rm CT}}_{{\rm tail}(k),j}$ (resp., ${e^{\rm TC}}_{{\rm head}(k),j}$) for some $j \in [1,t_{\rm T}]$ has edge-configuration γ;
- $\delta_{\rm ec,C}^{\rm CF}(c,[\gamma]^{\rm int}) \in [0,1], c \in [1,\widetilde{t_{\rm C}}], \gamma \in \widetilde{\Gamma}_{\rm ec}^{\rm CF}$: $\delta_{\rm ec,C}^{\rm CF}(c,[\gamma]^{\rm int}) = 1 \Leftrightarrow$ edge ${e^{\rm CF}}_{c,i}$ for some $i \in [1,t_{\rm F}]$ has edge-configuration γ;
- $\delta_{\rm ec,T}^{\rm TF}(i,[\gamma]^{\rm int}) \in [0,1], i \in [1,t_{\rm T}], \gamma \in \widetilde{\Gamma}_{\rm ec}^{\rm TF}$: $\delta_{\rm ec,T}^{\rm TF}(i,[\gamma]^{\rm int}) = 1 \Leftrightarrow$ edge ${e^{\rm TF}}_{i,j}$ for some $j \in [1,t_{\rm F}]$ has edge-configuration γ;
- $\deg_{\rm T}^{\rm CT}(k), \deg_{\rm T}^{\rm TC}(k) \in [0,4], k \in [1,k_{\rm C}]$: $\deg_G(v)$ of an end-vertex $v \in V_{\rm T}$ of the edge $({v^{\rm C}}_{{\rm tail}(k)}, v) \in E_{\rm CT}$ (resp., $(v, {v^{\rm C}}_{{\rm head}(k)}) \in E_{\rm TC}$) if any;
- $\deg_{\rm F}^{\rm CF}(c) \in [0,4], c \in [1,\widetilde{t_{\rm C}}]$: $\deg_G(v)$ of an end-vertex $v \in V_{\rm F}$ of the edge $({v^{\rm C}}_c, v) \in E_{\rm CF}$ if any;
- $\deg_{\rm F}^{\rm TF}(i) \in [0,4], i \in [1,t_{\rm T}]$: $\deg_G(v)$ of an end-vertex $v \in V_{\rm F}$ of the edge $({v^{\rm T}}_i, v) \in E_{\rm TF}$ if any;
- $\Delta_{\rm ec}^{\rm C+}(i), \Delta_{\rm ec}^{\rm C-}(i), \in [0,4], i \in [\widetilde{k_{\rm C}}+1, m_{\rm C}]$, $\Delta_{\rm ec}^{\rm T+}(i), \Delta_{\rm ec}^{\rm T-}(i) \in [0,4], i \in [2,t_{\rm T}]$, $\Delta_{\rm ec}^{\rm F+}(i), \Delta_{\rm ec}^{\rm F-}(i) \in [0,4], i \in [2,t_{\rm F}]$: $\Delta_{\rm ec}^{\rm X+}(i) = \Delta_{\rm ec}^{\rm X-}(i) = 0$ (resp., $\Delta_{\rm ec}^{\rm X+}(i) = \deg_G(u)$ and $\Delta_{\rm ec}^{\rm X-}(i) = \deg_G(v)$) $\Leftrightarrow$ edge ${e^{\rm X}}_i = (u,v) \in E_{\rm X}$ is used in G (resp., ${e^{\rm X}}_i \notin E(G)$);
- $\Delta_{\rm ec}^{\rm CT+}(k), \Delta_{\rm ec}^{\rm CT-}(k) \in [0,4], k \in [1,k_{\rm C}] = I_{(\geq 2)} \cup I_{(\geq 1)}$: $\Delta_{\rm ec}^{\rm CT+}(k) = \Delta_{\rm ec}^{\rm CT-}(k) = 0$ (resp., $\Delta_{\rm ec}^{\rm CT+}(k) = \deg_G(u)$ and $\Delta_{\rm ec}^{\rm CT-}(k) = \deg_G(v)$) $\Leftrightarrow$ edge ${e^{\rm CT}}_{{\rm tail}(k),j} = (u,v) \in E_{\rm CT}$ for some $j \in [1,t_{\rm T}]$ is used in G (resp., otherwise);
- $\Delta_{\rm ec}^{\rm TC+}(k), \Delta_{\rm ec}^{\rm TC-}(k) \in [0,4], k \in [1,k_{\rm C}] = I_{(\geq 2)} \cup I_{(\geq 1)}$: Analogous with $\Delta_{\rm ec}^{\rm CT+}(k)$ and $\Delta_{\rm ec}^{\rm CT-}(k)$;
- $\Delta_{\rm ac}^{\rm CF+}(c), \Delta_{\rm ec}^{\rm CF-}(c) \in [0,4], c \in [1,\widetilde{t_{\rm C}}]$: $\Delta_{\rm ec}^{\rm CF+}(c) = \Delta_{\rm ec}^{\rm CF-}(c) = 0$ (resp., $\Delta_{\rm ec}^{\rm CF+}(c) = \deg_G(u)$ and $\Delta_{\rm ec}^{\rm CF-}(c) = \deg_G(v)$) $\Leftrightarrow$ edge ${e^{\rm CF}}_{c,j} = (u,v) \in E_{\rm CF}$ for some $j \in [1,t_{\rm F}]$ is used in G (resp., otherwise);
- $\Delta_{\rm ec}^{\rm TF+}(i), \Delta_{\rm ec}^{\rm TF-}(i) \in [0,4], i \in [1,t_{\rm T}]$: Analogous with $\Delta_{\rm ec}^{\rm CF+}(c)$ and $\Delta_{\rm ec}^{\rm CF-}(c)$;

constraints:

$$\begin{aligned}
{\rm ec}_{\rm C}([\gamma]^{\rm int}) &= 0, & \gamma &\in \Gamma^{\rm int} \setminus \widetilde{\Gamma}_{\rm ec}^{\rm C}, \\
{\rm ec}_{\rm T}([\gamma]^{\rm int}) &= 0, & \gamma &\in \Gamma^{\rm int} \setminus \widetilde{\Gamma}_{\rm ec}^{\rm T}, \\
{\rm ec}_{\rm F}([\gamma]^{\rm int}) &= 0, & \gamma &\in \Gamma^{\rm int} \setminus \widetilde{\Gamma}_{\rm ec}^{\rm F}, \\
{\rm ec}_{\rm CT}([\gamma]^{\rm int}) &= 0, & \gamma &\in \Gamma^{\rm int} \setminus \widetilde{\Gamma}_{\rm ec}^{\rm CT}, \\
{\rm ec}_{\rm TC}([\gamma]^{\rm int}) &= 0, & \gamma &\in \Gamma^{\rm int} \setminus \widetilde{\Gamma}_{\rm ec}^{\rm TC}, \\
{\rm ec}_{\rm CF}([\gamma]^{\rm int}) &= 0, & \gamma &\in \Gamma^{\rm int} \setminus \widetilde{\Gamma}_{\rm ec}^{\rm CF}, \\
{\rm ec}_{\rm TF}([\gamma]^{\rm int}) &= 0, & \gamma &\in \Gamma^{\rm int} \setminus \widetilde{\Gamma}_{\rm ec}^{\rm TF},
\end{aligned} \tag{74}$$

$$\begin{aligned}
\sum_{(\mu,\mu',m)=\gamma\in\Gamma^{\rm int}} \mathrm{ec}_{\rm C}([\gamma]^{\rm int}) &= \sum_{i\in[\widetilde{k_{\rm C}}+1,m_{\rm C}]} \delta^{\rm C}_{\beta}(i,m), && m\in[1,3],\\
\sum_{(\mu,\mu',m)=\gamma\in\Gamma^{\rm int}} \mathrm{ec}_{\rm T}([\gamma]^{\rm int}) &= \sum_{i\in[2,t_{\rm T}]} \delta^{\rm T}_{\beta}(i,m), && m\in[1,3],\\
\sum_{(\mu,\mu',m)=\gamma\in\Gamma^{\rm int}} \mathrm{ec}_{\rm F}([\gamma]^{\rm int}) &= \sum_{i\in[2,t_{\rm F}]} \delta^{\rm F}_{\beta}(i,m), && m\in[1,3],\\
\sum_{(\mu,\mu',m)=\gamma\in\Gamma^{\rm int}} \mathrm{ec}_{\rm CT}([\gamma]^{\rm int}) &= \sum_{k\in[1,k_{\rm C}]} \delta^{+}_{\beta}(k,m), && m\in[1,3],\\
\sum_{(\mu,\mu',m)=\gamma\in\Gamma^{\rm int}} \mathrm{ec}_{\rm TC}([\gamma]^{\rm int}) &= \sum_{k\in[1,k_{\rm C}]} \delta^{-}_{\beta}(k,m), && m\in[1,3],\\
\sum_{(\mu,\mu',m)=\gamma\in\Gamma^{\rm int}} \mathrm{ec}_{\rm CF}([\gamma]^{\rm int}) &= \sum_{c\in[1,\widetilde{t_{\rm C}}]} \delta^{\rm in}_{\beta}(c,m), && m\in[1,3],\\
\sum_{(\mu,\mu',m)=\gamma\in\Gamma^{\rm int}} \mathrm{ec}_{\rm TF}([\gamma]^{\rm int}) &= \sum_{c\in[\widetilde{t_{\rm C}}+1,c_{\rm F}]} \delta^{\rm in}_{\beta}(c,m), && m\in[1,3],
\end{aligned}\tag{75}$$

$$\begin{aligned}
\sum_{\gamma=(\mathtt{a}d,\mathtt{b}d',m)\in\widetilde{\Gamma}^{\rm C}_{\rm ec}} [(\mathtt{a},\mathtt{b},m)]^{\rm int}\cdot\delta^{\rm C}_{\rm ec}(i,[\gamma]^{\rm int}) &= \sum_{\nu\in\widetilde{\Gamma}^{\rm C}_{\rm ac}} [\nu]^{\rm int}\cdot\delta^{\rm C}_{\rm ac}(i,[\nu]^{\rm int}),\\
\Delta^{\rm C+}_{\rm ec}(i) + \sum_{\gamma=(\mathtt{a}d,\xi,m)\in\widetilde{\Gamma}^{\rm C}_{\rm ec}} d\cdot\delta^{\rm C}_{\rm ec}(i,[\gamma]^{\rm int}) &= \deg^{\rm C}(\mathrm{tail}(i)),\\
\Delta^{\rm C-}_{\rm ec}(i) + \sum_{\gamma=(\mu,\mathtt{b}d,m)\in\widetilde{\Gamma}^{\rm C}_{\rm ec}} d\cdot\delta^{\rm C}_{\rm ec}(i,[\gamma]^{\rm int}) &= \deg^{\rm C}(\mathrm{head}(i)),\\
\Delta^{\rm C+}_{\rm ec}(i)+\Delta^{\rm C-}_{\rm ec}(i) &\le 8(1-e^{\rm C}(i)), && i\in[\widetilde{k_{\rm C}}+1,m_{\rm C}],\\
\sum_{i\in[\widetilde{k_{\rm C}}+1,m_{\rm C}]} \delta^{\rm C}_{\rm ec}(i,[\gamma]^{\rm int}) &= \mathrm{ec}_{\rm C}([\gamma]^{\rm int}), && \gamma\in\widetilde{\Gamma}^{\rm C}_{\rm ec},
\end{aligned}\tag{76}$$

$$\begin{aligned}
\sum_{\gamma=(\mathtt{a}d,\mathtt{b}d',m)\in\widetilde{\Gamma}^{\rm T}_{\rm ec}} [(\mathtt{a},\mathtt{b},m)]^{\rm int}\cdot\delta^{\rm T}_{\rm ec}(i,[\gamma]^{\rm int}) &= \sum_{\nu\in\widetilde{\Gamma}^{\rm T}_{\rm ac}} [\nu]^{\rm int}\cdot\delta^{\rm T}_{\rm ac}(i,[\nu]^{\rm int}),\\
\Delta^{\rm T+}_{\rm ec}(i) + \sum_{\gamma=(\mathtt{a}d,\xi,m)\in\widetilde{\Gamma}^{\rm T}_{\rm ec}} d\cdot\delta^{\rm T}_{\rm ec}(i,[\gamma]^{\rm int}) &= \deg^{\rm T}(i-1),\\
\Delta^{\rm T-}_{\rm ec}(i) + \sum_{\gamma=(\mu,\mathtt{b}d,m)\in\widetilde{\Gamma}^{\rm T}_{\rm ec}} d\cdot\delta^{\rm T}_{\rm ec}(i,[\gamma]^{\rm int}) &= \deg^{\rm T}(i),\\
\Delta^{\rm T+}_{\rm ec}(i)+\Delta^{\rm T-}_{\rm ec}(i) &\le 8(1-e^{\rm T}(i)), && i\in[2,t_{\rm T}],\\
\sum_{i\in[2,t_{\rm T}]} \delta^{\rm T}_{\rm ec}(i,[\gamma]^{\rm int}) &= \mathrm{ec}_{\rm T}([\gamma]^{\rm int}), && \gamma\in\widetilde{\Gamma}^{\rm T}_{\rm ec},
\end{aligned}\tag{77}$$

$$\sum_{\gamma=(\mathtt{a}d,\mathtt{b}d',m)\in\widetilde{\Gamma}^{\rm F}_{\rm ec}} [(\mathtt{a},\mathtt{b},m)]^{\rm int}\cdot\delta^{\rm F}_{\rm ec}(i,[\gamma]^{\rm int}) = \sum_{\nu\in\widetilde{\Gamma}^{\rm F}_{\rm ac}} [\nu]^{\rm int}\cdot\delta^{\rm F}_{\rm ac}(i,[\nu]^{\rm int}),$$
$$\Delta^{\rm F+}_{\rm ec}(i) + \sum_{\gamma=(\mathtt{a}d,\xi,m)\in\widetilde{\Gamma}^{\rm F}_{\rm ec}} d\cdot\delta^{\rm F}_{\rm ec}(i,[\gamma]^{\rm int}) = \deg^{\rm F}(i-1),$$
$$\Delta^{\rm F-}_{\rm ec}(i) + \sum_{\gamma=(\mu,\mathtt{b}d,m)\in\widetilde{\Gamma}^{\rm F}_{\rm ec}} d\cdot\delta^{\rm F}_{\rm ec}(i,[\gamma]^{\rm int}) = \deg^{\rm F}(i),$$
$$\Delta^{\rm F+}_{\rm ec}(i) + \Delta^{\rm F-}_{\rm ec}(i) \le 8(1-e^{\rm F}(i)), \qquad i\in[2,t_{\rm F}],$$
$$\sum_{i\in[2,t_{\rm F}]} \delta^{\rm F}_{\rm ec}(i,[\gamma]^{\rm int}) = {\rm ec}_{\rm F}([\gamma]^{\rm int}), \qquad \gamma\in\widetilde{\Gamma}^{\rm F}_{\rm ec}, \qquad (78)$$

$$\deg^{\rm T}(i) + 4(1-\chi^{\rm T}(i,k)+e^{\rm T}(i)) \ge \deg^{\rm CT}_{\rm T}(k),$$
$$\deg^{\rm CT}_{\rm T}(k) \ge \deg^{\rm T}(i) - 4(1-\chi^{\rm T}(i,k)+e^{\rm T}(i)), \qquad i\in[1,t_{\rm T}],$$
$$\sum_{\gamma=(\mathtt{a}d,\mathtt{b}d',m)\in\widetilde{\Gamma}^{\rm CT}_{\rm ec}} [(\mathtt{a},\mathtt{b},m)]^{\rm int}\cdot\delta^{\rm CT}_{\rm ec,C}(k,[\gamma]^{\rm int}) = \sum_{\nu\in\widetilde{\Gamma}^{\rm CT}_{\rm ac}} [\nu]^{\rm int}\cdot\delta^{\rm CT}_{\rm ac}(k,[\nu]^{\rm int}),$$
$$\Delta^{\rm CT+}_{\rm ec}(k) + \sum_{\gamma=(\mathtt{a}d,\xi,m)\in\widetilde{\Gamma}^{\rm CT}_{\rm ec}} d\cdot\delta^{\rm CT}_{\rm ec,C}(k,[\gamma]^{\rm int}) = \deg^{\rm C}({\rm tail}(k)),$$
$$\Delta^{\rm CT-}_{\rm ec}(k) + \sum_{\gamma=(\mu,\mathtt{b}d,m)\in\widetilde{\Gamma}^{\rm CT}_{\rm ec}} d\cdot\delta^{\rm CT}_{\rm ec,C}(k,[\gamma]^{\rm int}) = \deg^{\rm CT}_{\rm T}(k),$$
$$\Delta^{\rm CT+}_{\rm ec}(k) + \Delta^{\rm CT-}_{\rm ec}(k) \le 8(1-\delta^{\rm T}_{\chi}(k)), \qquad k\in[1,k_{\rm C}],$$
$$\sum_{k\in[1,k_{\rm C}]} \delta^{\rm CT}_{\rm ec,C}(k,[\gamma]^{\rm int}) = {\rm ec}_{\rm CT}([\gamma]^{\rm int}), \qquad \gamma\in\widetilde{\Gamma}^{\rm CT}_{\rm ec}, \qquad (79)$$

$$\deg^{\rm T}(i) + 4(1-\chi^{\rm T}(i,k)+e^{\rm T}(i+1)) \ge \deg^{\rm TC}_{\rm T}(k),$$
$$\deg^{\rm TC}_{\rm T}(k) \ge \deg^{\rm T}(i) - 4(1-\chi^{\rm T}(i,k)+e^{\rm T}(i+1)), \qquad i\in[1,t_{\rm T}],$$
$$\sum_{\gamma=(\mathtt{a}d,\mathtt{b}d',m)\in\widetilde{\Gamma}^{\rm TC}_{\rm ec}} [(\mathtt{a},\mathtt{b},m)]^{\rm int}\cdot\delta^{\rm TC}_{\rm ec,C}(k,[\gamma]^{\rm int}) = \sum_{\nu\in\widetilde{\Gamma}^{\rm TC}_{\rm ac}} [\nu]^{\rm int}\cdot\delta^{\rm TC}_{\rm ac}(k,[\nu]^{\rm int}),$$
$$\Delta^{\rm TC+}_{\rm ec}(k) + \sum_{\gamma=(\mathtt{a}d,\xi,m)\in\widetilde{\Gamma}^{\rm TC}_{\rm ec}} d\cdot\delta^{\rm TC}_{\rm ec,C}(k,[\gamma]^{\rm int}) = \deg^{\rm TC}_{\rm T}(k),$$
$$\Delta^{\rm TC-}_{\rm ec}(k) + \sum_{\gamma=(\mu,\mathtt{b}d,m)\in\widetilde{\Gamma}^{\rm TC}_{\rm ec}} d\cdot\delta^{\rm TC}_{\rm ec,C}(k,[\gamma]^{\rm int}) = \deg^{\rm C}({\rm head}(k)),$$
$$\Delta^{\rm TC+}_{\rm ec}(k) + \Delta^{\rm TC-}_{\rm ec}(k) \le 8(1-\delta^{\rm T}_{\chi}(k)), \qquad k\in[1,k_{\rm C}],$$
$$\sum_{k\in[1,k_{\rm C}]} \delta^{\rm TC}_{\rm ec,C}(k,[\gamma]^{\rm int}) = {\rm ec}_{\rm TC}([\gamma]^{\rm int}), \qquad \gamma\in\widetilde{\Gamma}^{\rm TC}_{\rm ec}, \qquad (80)$$

$$\begin{aligned}
\deg^{\mathrm{F}}(i) + 4(1-\chi^{\mathrm{F}}(i,c)+e^{\mathrm{F}}(i)) &\geq \deg^{\mathrm{CF}}_{\mathrm{F}}(c), \\
\deg^{\mathrm{CF}}_{\mathrm{F}}(c) &\geq \deg^{\mathrm{F}}(i) - 4(1-\chi^{\mathrm{F}}(i,c)+e^{\mathrm{F}}(i)), && i\in[1,t_{\mathrm{F}}], \\
\sum_{\gamma=(\mathtt{a}d,\mathtt{b}d',m)\in\widetilde{\Gamma}^{\mathrm{CF}}_{\mathrm{ec}}} [(\mathtt{a},\mathtt{b},m)]^{\mathrm{int}}\cdot\delta^{\mathrm{CF}}_{\mathrm{ec,C}}(c,[\gamma]^{\mathrm{int}}) &= \sum_{\nu\in\widetilde{\Gamma}^{\mathrm{CF}}_{\mathrm{ac}}}[\nu]^{\mathrm{int}}\cdot\delta^{\mathrm{CF}}_{\mathrm{ac}}(c,[\nu]^{\mathrm{int}}), \\
\Delta^{\mathrm{CF}+}_{\mathrm{ec}}(c) + \sum_{\gamma=(\mathtt{a}d,\xi,m)\in\widetilde{\Gamma}^{\mathrm{CF}}_{\mathrm{ec}}} d\cdot\delta^{\mathrm{CF}}_{\mathrm{ec,C}}(c,[\gamma]^{\mathrm{int}}) &= \deg^{\mathrm{C}}(c), \\
\Delta^{\mathrm{CF}-}_{\mathrm{ec}}(c) + \sum_{\gamma=(\mu,\mathtt{b}d,m)\in\widetilde{\Gamma}^{\mathrm{CF}}_{\mathrm{ec}}} d\cdot\delta^{\mathrm{CF}}_{\mathrm{ec,C}}(c,[\gamma]^{\mathrm{int}}) &= \deg^{\mathrm{CF}}_{\mathrm{F}}(c), \\
\Delta^{\mathrm{CF}+}_{\mathrm{ec}}(c) + \Delta^{\mathrm{CF}-}_{\mathrm{ec}}(c) &\leq 8(1-\delta^{\mathrm{F}}_{\chi}(c)), && c\in[1,\widetilde{t_{\mathrm{C}}}], \\
\sum_{c\in[1,\widetilde{t_{\mathrm{C}}}]}\delta^{\mathrm{CF}}_{\mathrm{ec,C}}(c,[\gamma]^{\mathrm{int}}) &= \mathrm{ec}_{\mathrm{CF}}([\gamma]^{\mathrm{int}}), && \gamma\in\widetilde{\Gamma}^{\mathrm{CF}}_{\mathrm{ec}}, \qquad (81)
\end{aligned}$$

$$\begin{aligned}
\deg^{\mathrm{F}}(j) + 4(1-\chi^{\mathrm{F}}(j,i+\widetilde{t_{\mathrm{C}}})+e^{\mathrm{F}}(j)) &\geq \deg^{\mathrm{TF}}_{\mathrm{F}}(i), \\
\deg^{\mathrm{TF}}_{\mathrm{F}}(i) &\geq \deg^{\mathrm{F}}(j) - 4(1-\chi^{\mathrm{F}}(j,i+\widetilde{t_{\mathrm{C}}})+e^{\mathrm{F}}(j)), && j\in[1,t_{\mathrm{F}}], \\
\sum_{\gamma=(\mathtt{a}d,\mathtt{b}d',m)\in\widetilde{\Gamma}^{\mathrm{TF}}_{\mathrm{ec}}} [(\mathtt{a},\mathtt{b},m)]^{\mathrm{int}}\cdot\delta^{\mathrm{TF}}_{\mathrm{ec,T}}(i,[\gamma]^{\mathrm{int}}) &= \sum_{\nu\in\widetilde{\Gamma}^{\mathrm{TF}}_{\mathrm{ac}}}[\nu]^{\mathrm{int}}\cdot\delta^{\mathrm{TF}}_{\mathrm{ac}}(i,[\nu]^{\mathrm{int}}), \\
\Delta^{\mathrm{TF}+}_{\mathrm{ec}}(i) + \sum_{\gamma=(\mathtt{a}d,\xi,m)\in\widetilde{\Gamma}^{\mathrm{TF}}_{\mathrm{ec}}} d\cdot\delta^{\mathrm{TF}}_{\mathrm{ec,T}}(i,[\gamma]^{\mathrm{int}}) &= \deg^{\mathrm{T}}(i), \\
\Delta^{\mathrm{TF}-}_{\mathrm{ec}}(i) + \sum_{\gamma=(\mu,\mathtt{b}d,m)\in\widetilde{\Gamma}^{\mathrm{TF}}_{\mathrm{ec}}} d\cdot\delta^{\mathrm{TF}}_{\mathrm{ec,T}}(i,[\gamma]^{\mathrm{int}}) &= \deg^{\mathrm{TF}}_{\mathrm{F}}(i), \\
\Delta^{\mathrm{TF}+}_{\mathrm{ec}}(i) + \Delta^{\mathrm{TF}-}_{\mathrm{ec}}(i) &\leq 8(1-\delta^{\mathrm{F}}_{\chi}(i+\widetilde{t_{\mathrm{C}}})), && i\in[1,t_{\mathrm{T}}], \\
\sum_{i\in[1,t_{\mathrm{T}}]}\delta^{\mathrm{TF}}_{\mathrm{ec,T}}(i,[\gamma]^{\mathrm{int}}) &= \mathrm{ec}_{\mathrm{TF}}([\gamma]^{\mathrm{int}}), && \gamma\in\widetilde{\Gamma}^{\mathrm{TF}}_{\mathrm{ec}}, \qquad (82)
\end{aligned}$$

$$\begin{aligned}
\sum_{\mathrm{X}\in\{\mathrm{C,T,F,CT,TC,CF,TF}\}}(\mathrm{ec}_{\mathrm{X}}([\gamma]^{\mathrm{int}})+\mathrm{ec}_{\mathrm{X}}([\overline{\gamma}]^{\mathrm{int}})) &= \mathrm{ec}^{\mathrm{int}}([\gamma]^{\mathrm{int}}), && \gamma\in\Gamma^{\mathrm{int}}_{<}, \\
\sum_{\mathrm{X}\in\{\mathrm{C,T,F,CT,TC,CF,TF}\}}\mathrm{ec}_{\mathrm{X}}([\gamma]^{\mathrm{int}}) &= \mathrm{ec}^{\mathrm{int}}([\gamma]^{\mathrm{int}}), && \gamma\in\Gamma^{\mathrm{int}}_{=}. \qquad (83)
\end{aligned}$$

3.11 Descriptor for the Number of of Fringe-configurations

We include constraints to compute the frequency of each fringe-configuration in an inferred chemical graph G.

variables:

$\mathrm{fc}([\psi])\in[0,t_{\mathrm{C}}+t_{\mathrm{T}}+t_{\mathrm{F}}], \psi\in\mathcal{F}^*$: the frequency of a chemical rooted tree ψ in the set of ρ-fringe-trees in G;

constraints:

$$\sum_{i\in[1,t_{\rm X}],{\rm X}\in\{{\rm C,T,F}\}} \delta_{\rm fr}^{\rm X}(i,[\psi]) = {\rm fc}([\psi]), \qquad \psi \in \mathcal{F}^*. \qquad (84)$$

3.12 Constraints for Normalization of Feature Vectors

By introducing a tolerance $\varepsilon > 0$ in the conversion between integers and reals, we include the following constraints for normalizing of a feature vector $f(G) = (x_1, x_2, \ldots, x_K)$:

$$\frac{(1-\varepsilon)(x_i - \min({\rm dcp}_i; D_\pi))}{\max({\rm dcp}_i; D_\pi) - \min({\rm dcp}_i; D_\pi)} \leq \widehat{x}_i \leq \frac{(1+\varepsilon)(x_i - \min({\rm dcp}_i; D_\pi))}{\max({\rm dcp}_i; D_\pi) - \min({\rm dcp}_i; D_\pi)}, \ i \in [1, K]. \qquad (85)$$

An example of a tolerance is $\varepsilon = 0.01$.

References

[1] R. Ito, N. A. Azam, C. Wang, A. Shurbevski, H. Nagamochi, T. Akutsu, A novel method for the inverse QSAR/QSPR to monocyclic chemical compounds based on artificial neural networks and integer programming, *BIOCOMP2020*, Las Vegas, Nevada, USA, 27-30 July 2020.

[2] F. Zhang, J. Zhu, R. Chiewvanichakorn, A. Shurbevski, H. Nagamochi, T. Akutsu, A new integer linear programming formulation to the inverse QSAR/QSPR for acyclic chemical compounds using skeleton trees, *The 33rd International Conference on Industrial, Engineering and Other Applications of Applied Intelligent Systems*, September 22-25, 2020 Kitakyushu, Japan, Springer LNCS 12144, pp. 433–444.

[3] N. A. Azam, J. Zhu, Y. Sun, Y. Shi, A. Shurbevski, L. Zhao, H. Nagamochi, T. Akutsu, A novel method for inference of acyclic chemical compounds with bounded branch-height based on artificial neural networks and integer programming, arXiv:2009.09646

[4] T. Akutsu, H. Nagamochi, A novel method for inference of chemical compounds with prescribed topological substructures based on integer programming, arXiv: 2010.09203, 2020.
